# Topography‐driven microclimate gradients shape forest structure, diversity, and composition in a temperate refugial forest

**DOI:** 10.1002/pei3.10153

**Published:** 2024-06-11

**Authors:** Bailey H. McNichol, Ran Wang, Amanda Hefner, Chris Helzer, Sean M. McMahon, Sabrina E. Russo

**Affiliations:** ^1^ School of Biological Sciences University of Nebraska–Lincoln Lincoln Nebraska USA; ^2^ School of Natural Resources University of Nebraska–Lincoln Lincoln Nebraska USA; ^3^ The Nature Conservancy Omaha Nebraska USA; ^4^ Smithsonian Institution Forest Global Earth Observatory Smithsonian Environmental Research Center Edgewater Maryland USA; ^5^ Center for Plant Science Innovation University of Nebraska–Lincoln Lincoln Nebraska USA

**Keywords:** climate‐change refugia, ecotone, forest biome, microclimate, refugial forest, topographic gradient, water availability

## Abstract

Macroclimate drives vegetation distributions, but fine‐scale topographic variation can generate microclimate refugia for plant persistence in unsuitable areas. However, we lack quantitative descriptions of topography‐driven microclimatic variation and how it shapes forest structure, diversity, and composition. We hypothesized that topographic variation and the presence of the forest overstory cause spatiotemporal microclimate variation affecting tree performance, causing forest structure, diversity, and composition to vary with topography and microclimate, and topography and the overstory to buffer microclimate. In a 20.2‐ha inventory plot in the North American Great Plains, we censused woody stems ≥1 cm in diameter and collected detailed topographic and microclimatic data. Across 59‐m of elevation, microclimate covaried with topography to create a sharp desiccation gradient, and topography and the overstory buffered understory microclimate. The magnitude of microclimatic variation mirrored that of regional‐scale variation: with increasing elevation, there was a decrease in soil moisture corresponding to the difference across ~2.1° of longitude along the east‐to‐west aridity gradient and an increase in air temperature corresponding to the difference across ~2.7° of latitude along the north‐to‐south gradient. More complex forest structure and higher diversity occurred in moister, less‐exposed habitats, and species occupied distinct topographic niches. Our study demonstrates how topographic and microclimatic gradients structure forests in putative climate‐change refugia, by revealing ecological processes enabling populations to be maintained during periods of unfavorable macroclimate.

## Graphical abstract

We hypothesized that topographic variation and the forest overstory cause spatiotemporal microclimate variation affecting tree performance, causing forest structure, diversity, and composition to vary with topography and microclimate, and topography and the overstory to buffer microclimate. We found that microclimate covaried strongly with topography, and topography and the overstory buffered microclimate, facilitating a more complex forest structure and higher diversity in moister, less‐exposed habitats. Our study demonstrates how topographic and microclimatic gradients structure forests in putative climate‐change refugia, by revealing ecological processes enabling populations to be maintained during periods of unfavorable macroclimate.

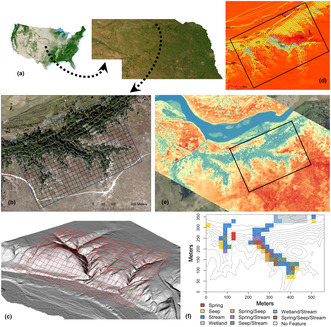

## 1 INTRODUCTION

Understanding and predicting the effects of global climate change on vegetation structure, diversity, and composition is an important focus of ecological research. This is particularly true for forests, as they are critical providers of ecosystem services and regulators of climate from local to global scales (Anderson‐Teixeira et al., 2013; FAO & UNEP, 2020; Lawrence & Vandecar, 2015). Changes in climate regimes at large spatial scales are anticipated to shift the distribution of suitable environments for forests (Davis & Shaw, 2001; Hampe & Jump, 2011), likely causing some plant populations within a species' geographic range to become poorly matched to local conditions (McNichol & Russo, 2023). However, many forest plant species' ranges are projected to shift to higher latitudes or altitudes to track their climatic niche (Feeley et al., 2011; Skov & Svenning, 2004; Vellend et al., 2021). Some populations may persist within climate‐change refugia, areas of limited spatial extent that are relatively buffered from a changing macroclimate owing to local topographic, hydrologic, and hydrogeologic conditions (Cartwright et al., 2020; Morelli et al., 2016; Stralberg et al., 2020; Wilkin et al., 2016). Historically, climate‐change refugia have maintained components of regional diversity over long timescales (*i.e.*, millennia) as the macroclimate has changed (Ackerly et al., 2020; Tzedakis et al., 2002), occurring in physiographic settings (*e.g.*, northern aspects, sheltered valleys) that support climatic conditions that were once widespread regionally (Birks & Willis, 2008; Dobrowski, 2011). Climate‐change refugia are, therefore, likely to be critical in buffering sensitive species from current and future changes in macroclimate (McLaughlin et al., 2017; Stark et al., 2022; Stralberg et al., 2020).

While global climate models have established that for most regions, both average and variation in climate are changing (IPCC, 2019), we have limited information on how large‐scale climatic shifts will translate into changes in the conditions that trees experience at small scales (hereafter, “microclimate,” or climate at the individual to sub‐stand scale) (De Frenne & Verheyen, 2016; Hofmeister et al., 2019; Pool et al., 1918). Microclimate directly influences the recruitment and persistence of woody plant species, and the presence of the forest overstory can further buffer understory conditions, contributing to a cooler, moister, less exposed microclimate (Davis et al., 2019; De Frenne et al., 2019; Villegas et al., 2010). Topographic variation can also drive shifts in forest structure, diversity, and composition along local gradients (Jucker et al., 2018; Marca‐Zevallos et al., 2022; Russo et al., 2005). Therefore, the structure of the forest canopy, coupled with fine‐scale heterogeneity in topography, can play a crucial role in buffering microclimate conditions (Estevo et al., 2022; Lawrence et al., 2021; Zellweger et al., 2020), maintaining climate‐change refugia as macroclimate changes (Dobrowski, 2011; Landuyt et al., 2019; Rita et al., 2021). We define microclimate buffering in the context of the northern hemisphere temperate growing season (approximately May to October) to include decreased air and soil temperatures and increased soil moisture and relative humidity that can occur due to the presence of the forest overstory (De Frenne et al., 2021), and due to underlying topographic features (*e.g.*, elevational depressions or valleys, sheltered slopes, north‐facing aspects) and hydrologic features (*e.g.*, presence of groundwater springs and seeps) (Cartwright et al., 2020; Morelli et al., 2016). Understanding both the magnitude of variation in and the responses of individuals and populations to local microclimate conditions is crucial, as these responses, in aggregate, drive forest change (Blonder et al., 2018; Harwood et al., 2014).

Describing topography‐driven microclimate buffering and its potential to produce climate‐change refugia (Dobrowski et al., 2021; Stralberg et al., 2020; Stralberg, Carroll, & Nielsen, 2020) is crucial for understanding and predicting how plants may shift their distributions in response to climate change. However, much of this work has been conducted in alpine systems (Foster & D'Amato, 2015; Scherrer & Körner, 2010) and has focused on variation in temperature, although numerous other environmental variables (*e.g.*, soil temperature and moisture, relative humidity, vapor pressure deficit) can also strongly affect plant growth, survival, and reproduction (Bauman et al., 2022; Breshears et al., 2013; Eamus et al., 2013). These other climatic variables are infrequently measured at fine spatial scales (Blonder et al., 2018; Davis et al., 2019), limiting our understanding of the multiple dimensions of microclimate that are important for creating forest refugia.

**FIGURE 1:**
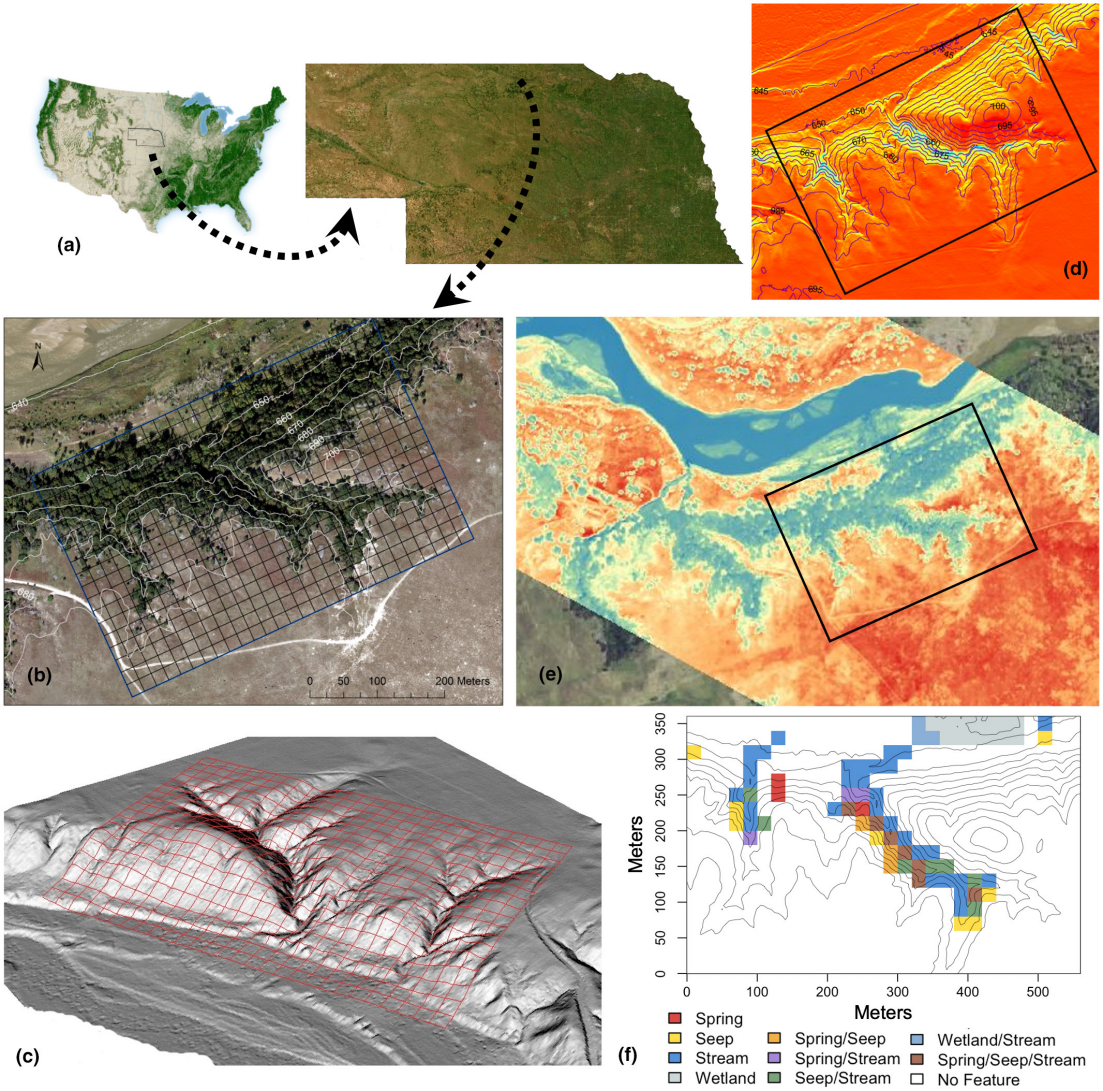
Location, topographic gradients, and hydrological features of the 20.2 ha Niobrara forest dynamics plot sampling refugial forest in the Niobrara River Valley of Nebraska in the Great Plains, USA. (a) Location of the Niobrara plot, with respect to a map of forest cover in the continental United States (taken from the U.S. Forest Service's online Forest Atlas of the United States) and a 15‐m resolution aerial map of Nebraska (Image source: Esri Inc, 2022); (b) a high‐resolution RGBN image obtained on 18 September 2019, with an UltraCam Eagle camera (Vexcel Imaging), showing the boundary of the Niobrara plot and 20c20 m quadrats with 10‐m elevation contours; (c) digital elevation model (DEM) used to estimate the five topographic variables (elevation, solar radiation, slope, eastness, and northness), with the Niobrara plot and quadrat boundaries overlaid (U.S. Geological Survey, 2017); (d) a map of cumulative annual solar radiation [Wh/m^2^; red‐yellow‐blue color ramp indicates high to low radiation showing gradients in exposure within the Niobrara plot with respect to elevation contours, based on the DEM shown in (c)]; (e) a thermal image (blue colors depict cooler, and red colors depict hotter, relative temperatures) of the region with the Niobrara plot boundary in black (image obtained on 13 July 2022); (f) a map of the quadrats in the Niobrara plot containing hydrological and hydrogeological features (as indicated in legend) originating from spring‐fed canyon streams (springs, seeps, streams) and the water table (wetland), overlaid with 5‐m elevation contour lines.

In this study, we evaluated the extent to which topographically driven variation in microclimate shapes the structure, diversity, and composition of a mature forest in a historical forest refugium in the Great Plains of North America, where the semi‐arid macroclimate constrains the predominant biome to grassland. In the Niobrara River Valley, Nebraska, USA, forests occur in cooler, moister north‐facing canyons draining to the Niobrara River (Kaul et al., 1988) (Figure 1). While the forest canopy itself further ameliorates conditions in these canyons, the existence of this forest in the first place owes to the topographic, hydrologic, and hydrogeologic conditions that created climate‐change refugia that are thought to have supported remnant tree species populations since the Last Glacial Maximum (Bessey, 1905; Kaul et al., 1988) (Supporting Information Figure S1). The populations of most tree species in the Niobrara forests are not only at the edges of their habitat and climatic ranges but also at their geographic range edges and represent species from predominantly western, boreal, and eastern distributions, as well as species more widespread in the Great Plains and Midwest (Kaul et al., 1988; Tolstead, 1942b). How topography structures microclimate and shapes the distribution of diversity within these refugia may, therefore, parallel the role of climate in shaping species' geographic distributions.

**FIGURE 2:**
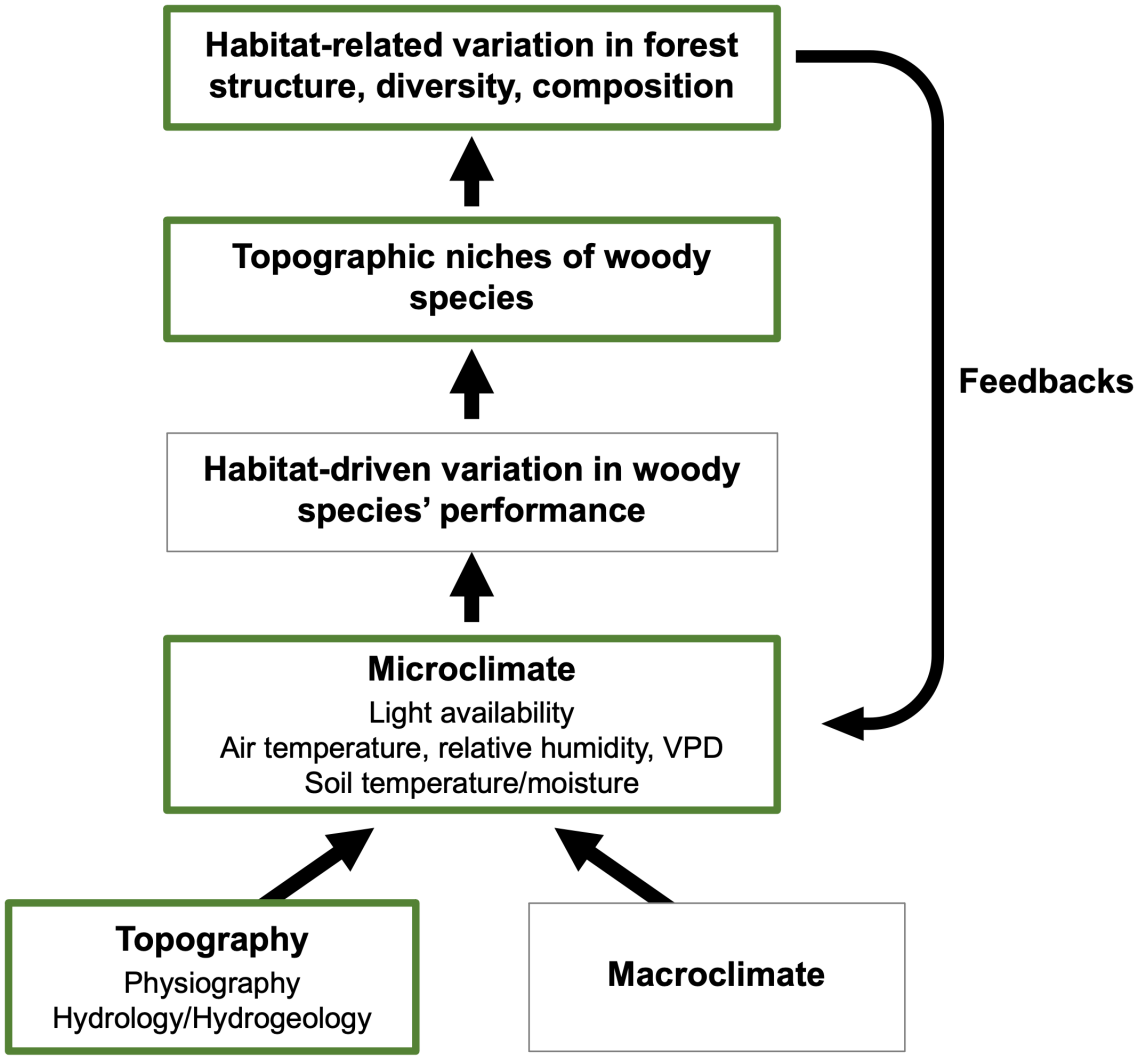
Conceptual model illustrating how topographically driven microclimatic gradients shape patterns of forest structure, diversity, and composition. Macroclimate and variation in topography – which encompasses physiography, hydrological, and hydrogeological features – cause spatial and temporal variation in microclimatic variables known to influence the performance of woody species, shaping species' topographic niches, and driving variation in forest structure, diversity, and composition. Habitat‐related variation in forest structure, diversity, and composition contribute to feedbacks influencing microclimatic variation, with downstream consequences for species' performance across life stages. Attributes in boxes outlined in thicker, green lines were directly evaluated in our study, and topographically defined habitats are shown in Figure 5A.

We hypothesized that variation in topography and the presence of the forest overstory cause spatial and temporal variation in microclimatic conditions known to affect the performance of woody species (Figure 2). If so, then forest structure, diversity, and composition should vary strongly with topography and microclimate, and topographic variation and the overstory should mediate microclimate buffering in the understory. We tested this hypothesis in a 20.2‐ha forest plot encompassing the Niobrara forests by coupling fully georeferenced tree inventory data, topographic data on elevation, slope, aspect, and cumulative annual solar radiation from a digital elevation model (DEM), and two years of locally sampled microclimate data (understory light availability, air temperature, and relative humidity, vapor pressure deficit, and soil temperature and moisture). Our hypothesis leads to the following predictions: (P1) There should be correlated variation in topography and microclimate, and both the forest overstory and topography should have buffering effects on understory microclimate conditions. (P2) Woody species should occupy distinct topographic niches that correspond to their geographic ranges, causing (P3) forest structure, diversity, and composition to vary with topography and (P4) with microclimate.

## 2 MATERIALS AND METHODS

### 2.1 Study site and census methods

The Niobrara Forest Inventory Plot (hereafter, Niobrara plot; 42°46′48.83″ N, 100°01′15.56″ W; Figure 1) is part of the Smithsonian Forest Global Earth Observatory (ForestGEO) network of forest plots (Davies et al., 2021). The 20.2‐ha Niobrara plot was established in 2019 within The Nature Conservancy's Niobrara Valley Preserve (Johnstown, Nebraska, USA) (Figure 1a,b). From 2000 to 2015, the average cumulative annual precipitation on the Niobrara plot was 55.6 cm, the mean annual temperature was 9.6°C and minimum and maximum daily temperatures were − 11.3 and 32°C (PRISM Climate Group, 2020). This climate regime is within the temperate grassland biome, near the boundary with the woodland/shrubland biome (Chapin III et al., 2011; Whittaker, 1975). Soils are entisols, including loamy fine sands and excessively drained sand, and are characterized by little development of soil profile horizons (Soil Survey Staff, USDA NRCS, 2015, 2019). Forests in the canyons of the south side of the Niobrara River valley are bordered to the south by Sandhills grassland, creating a dramatic forest‐grassland transition zone (Kaul et al., 1988) (Figure 1b) with defined thermal environments visible at large scales (Figure 1e). The Niobrara plot encompasses a 59‐m elevational gradient (644–703 m.a.s.l.) from the Niobrara River floodplain to the forest‐grassland transition zone (Figure 1b,c). As the soils of the Sandhills are >90% sand (Whitcomb, 1989), they are extremely well‐drained, and the steep slopes and complex topography expose groundwater seeps supporting spring‐fed streams in the canyons (Hearty, 1978; Tolstead, 1942a) (Figure 1f, S2).

The Niobrara plot (560 × 360 m) is divided into a grid of 504 20 × 20 m quadrats (Figure 1b,c) permanently marked by georeferenced posts. Following standardized ForestGEO methods (Condit, 1998), all woody stems (trees, shrubs, lianas) ≥1 cm in trunk diameter at breast height (1.3 m, DBH) were permanently tagged (including multiple stems on the same individual), identified to species, measured for DBH, and georeferenced. In the first census, 339 of the 504 quadrats (67%) had ≥1 individual, for 8299 total individuals of 27 species (Tables S1, S2).

### 2.2 Estimation of DEM‐derived topographic variables

Using a 1‐m resolution DEM derived from remotely sensed Light Detection and Ranging (LiDAR) data (U.S. Geological Survey, 2017), we estimated five topographic variables in the Niobrara plot, elevation (m), slope (%), cumulative annual solar radiation (Wh/m^2^), and two variables representing aspect, northness and eastness (Figure 1b–d). For each variable, we averaged 1‐m scale values to obtain quadrat‐level means and estimated values for each tagged individual by matching coordinates. We define topography to include physiography (*i.e.*, slope, aspect, elevation, solar radiation) and the associated variation in hydrological and hydrogeological features (*e.g.*, groundwater‐to‐surface water flow) that physiographic variation causes, which together influence variation in microclimate (Figure 2).

### 2.3 Monitoring of microclimate along topographic gradients

We quantified variation in microclimate from just before to just after the 2021 and 2022 Northern Hemisphere growing seasons (approximately April to November) at ten monitoring stations deployed along the topographic gradients in the Niobrara plot. Stations 1–9 were located in areas with tree cover, and Station 10 was located in grassland, just past the forest‐grassland transition (Figure S2). At each station, understory light availability (photosynthetic photon flux density; PPFD, μmol/m^2^/s;) was measured with 1–2 quantum sensors, and air temperature (°C) and relative humidity (RH, %) were measured at ~1 m above the ground. Surface soil temperature (°C) was measured at a depth of 10 cm, and soil moisture (volumetric water content; VWC, %) was measured with 1–2 time‐domain reflectometry sensors to a depth of 30 cm. At the station in the grassland (Station 10), a tipping bucket was deployed to measure rainfall (cm). Values were temporarily logged at fine timescales, and 1‐ and 5‐min averages were recorded for PPFD and the other variables, respectively. We derived vapor pressure deficit (VPD, kPa) from air temperature and relative humidity using Tetens' formula. The microclimate stations measured conditions at fine timescales but at only ten locations. To quantify variation in surface soil moisture with greater spatial coverage, manual point measurements of VWC (%) were taken using a hand‐held time‐domain reflectometer in 73 quadrats (canyon bottoms = 15, upland forest = 14, upper canyon = 14, floodplain = 15, ecotone = 15; see Materials and methods: *Statistical analysis* for habitat definitions), with three replicate measurements per quadrat. Measurements were taken on a single rain‐free day, on 6 days between May and October 2021.

### 2.4 Statistical analysis

Analyses were conducted in R software version 4.0.2 (R Core Team, 2022), using the “allodb”, “broom,” “chron,” “dplyr,” “ecodist,” “fgeo,” “lubridate,” “mgcv,” “missMDA,” “multcomp,” “MuMIn,” “nlme,” “padr,” “pander,” “purrr,” “quantreg,” “stats,” and “vegan” packages (Goslee & Urban, 2007; Hothorn et al., 2008; Grolemund & Wickham, 2011; Wood, 2011; Josse & Husson, 2016; Lepore et al., 2019; Henry & Wickham, 2020; Bartón, 2022; Daróczi & Tsegelskyi, 2022; Gonzalez‐Akre et al., 2022; James & Hornik, 2022; Koenker, 2022; Oksanen et al., 2022; Pinheiro et al., 2022; R Core Team, 2022; Robinson et al., 2022; Thoen, 2022; Wickham et al., 2022). Statistical significance was assessed at the α = .05 level.

*Derivation of habitat types*. To evaluate P1 and P2, we defined five categorical topographic habitats at the 20 × 20 m scale based on the five topographic variables using principal components analysis (PCA). The first principal component (PC1; 42.7% of variation) was correlated with slope, solar radiation, and elevation, and PC2 (22.6% of variation) was correlated with aspect (Figure S3a; Table S3). Habitats were defined using cutoffs for quadrat scores of PC1 and PC2 (Methods S1; Figure 5A). In the PCA‐defined ecotone habitat, we assigned a sixth habitat, grassland, for all quadrats with zero woody stems with DBH ≥1 cm (Figure 5A).

*P1 There should be correlated variation in topography and microclimate, and the forest overstory and topography should have buffering effects on understory microclimate*. Analyses involving microclimate were conducted separately on the 2021 and 2022 data. Daily means of microclimate variables were calculated for each station, from which monthly means and coefficients of variation (standard deviation/mean × 100; CV) were calculated to estimate the mean microclimate and microclimate variability at each station. We ran separate PCAs on monthly microclimate means and CVs from each station and used permutational multivariate analyses of variance (perMANOVA) to assess the effects of habitat and the habitat‐month interaction on the between‐station Gower dissimilarities in microclimate means and CVs. To assess if microclimatic variation is driven by continuous topographic variation, we extracted the first three PCs from the topographic PCA used to define habitats and conducted perMANOVA to assess the effects of topographic PC1, PC2, PC3, and month on dissimilarities in microclimate means and CVs.

To assess variation in soil VWC between habitats and across the 2021 growing season, we fit a linear mixed‐effects model on the manually measured VWC data, with habitat and sampling timepoint as fixed effects and quadrat as a random effect. We assessed goodness of fit using pseudo‐*R* ^*2*^ values (*pR* ^2^) to quantify variance explained by fixed effects alone (marginal *pR* ^2^) and by both fixed and random effects (conditional *pR* ^2^). We used post hoc multiple comparisons to assess differences in VWC between habitats and time points, following statistically significant omnibus tests. To explore topographic constraints on variation in soil moisture, we fit quantile regressions with manually measured VWC as the response variable and the additive effects of sampling timepoint and one of either elevation, slope, or solar radiation as predictors in three separate models. Quantile regressions were implemented using the median and 95th quantile, and we assessed model goodness of fit using *pR* ^2^ values.

To assess the effect of forest overstory and topography on microclimate buffering, we calculated daily differences in average air temperature, soil temperature, VWC, RH, and VPD between each of the nine microclimate stations in the forest understory (Stations 1–9) versus the station in grassland (Station 10) and averaged differences within months (April–November). We calculated the total basal area in a 5‐m radius of each station and determined station elevations and northness from the DEM. We evaluated above‐ and belowground microclimate buffering using differences in air temperature, RH, and VPD, and soil temperature and VWC, respectively. We ran separate perMANOVAs for each year to test the effects of overstory (basal area) and topography (elevation or northness, which were included in separate models due to the modest number of stations) on microclimate buffering, where the response variable was a matrix of monthly mean above‐ or belowground microclimate differences between forested stations and the grassland station. To assess whether there was significant microclimate buffering within forested habitats, we ran paired *t*‐tests between mean monthly microclimate conditions at each forested station versus the grassland station.

*P2 Woody species should exhibit distinct topographic niches*. We evaluated whether species occupy distinct elevation niches based on whether their mean niche position and niche breadth differed from those expected by chance alone, accounting for the distribution of available elevations in forested parts of the Niobrara plot. We used conditional torus translation (Abiem et al., 2020) to translate trees of each species within each quadrat to new forested quadrats, maintaining the number of quadrats occupied by the species and preserving the effects of dispersal limitation by maintaining species' abundances and the spatial arrangement of individuals within the quadrat. The translation is conditional because trees could not be translated to grassland quadrats with no woody stems. For each species with ≥3 individuals in the Niobrara plot, we generated 999 translations. For the observed and translated distributions, the elevation of each tree was determined (see Materials and methods: *Estimation of DEM‐derived topographic variables*), and the conditional probability of a tree occurring at each elevation was estimated (Itoh et al., 2010). We tested whether species had distinct niche positions and narrower niches given the available elevations in the Niobrara plot than expected by chance using two‐tailed and one‐tailed tests based on the rank of species observed versus translated mean and standard deviation elevations, respectively.

*P3 Topography should correlate with forest structure, diversity, and composition*. To quantify metrics of forest structure and diversity, we calculated tree density (number of individuals/ha), total stem basal area (m^2^/ha), stem aboveground biomass (AGB; Mg/ha), species richness (number of species), and Shannon's diversity in each quadrat using the 339 treed quadrats. We used two modeling approaches to quantify topography‐driven variation in forest structure and diversity, both using quadrat as the unit of replication and applying Box‐Cox transformations to response variables to meet assumptions of normality. First, we used linear models with habitat type as a categorical fixed‐effect predictor. After a significant omnibus test, we implemented post‐hoc multiple comparisons between habitat types. Second, we used generalized additive models (GAMs) with the topographic variables as continuous predictors to assess their main and interactive effects on forest structure and diversity. To account for spatial autocorrelation, we included two‐dimensional thin plate splines of the quadrat coordinates, fitting models with a Gaussian error distribution and identity link function. There was no evidence of multicollinearity between topographic variables (variance inflation factors in the global model <2.5). We used model selection to find the most‐supported model among 59 candidate GAMs fitted for each response variable, based on Akaike's Information Criterion (AIC) and relative likelihoods of models using Akaike weights (Akaike, 1973). No three‐way interactions were significant, so the most complex (global) model included all two‐way interactions between the five topographic variables, and the simplest (null) model included the intercept only.

We quantified variation in tree composition with respect to categorical habitats and continuous topographic variables. We used a perMANOVA and analysis of similarity to assess the effect of habitat on tree species composition and used nonmetric multidimensional scaling (NMDS) to visualize variation among habitats. To test whether habitats differed in their variance in composition, we calculated the within‐habitat multivariate dispersion and compared this between habitats using post‐hoc multiple comparisons. To assess the effects of topography on composition, we conducted multiple regression with matrices (MRM) of the Cao dissimilarities as a function of Gower dissimilarities of topographic variables between quadrats. To partition variance explained by each variable, we estimated adjusted *R* ^2^ values from a full linear model with dissimilarities in all topographic variables as predictors and models excluding each of the variables, then subtracted their *R* ^2^ values from that of the full model (Swenson, 2014).

*P4 Forest structure, diversity, and composition should correlate with microclimate variation*. We defined a 10‐m radius around each of the nine forested microclimate stations and quantified forest structure (the number of trees, basal area, AGB) and richness (number of species) in each area and estimated Cao dissimilarities in composition between pairs of areas. For 2021 and 2022 separately, we calculated station‐level microclimate means and CVs from monthly VPD and soil temperature and VWC. Due to a limited number of stations, we included VPD, but not air RH nor temperature, in analyses, as VPD is strongly correlated with RH (*r* = −.99 in 2021; *r* = −.98 in 2022) and air temperature (*r* = .88 in 2021; *r* = .95 in 2022). We used separate linear models to determine the effects of the microclimate means and CVs on forest structure and richness. We used separate perMANOVAs to assess the effects of microclimate means and CVs on dissimilarities in species composition.

## 3 RESULTS

### 3.1 P1 Strong microclimatic variation between habitats and along topographic gradients, and significant understory microclimate buffering attributable to topography and the forest overstory

Microclimate and microclimate variability differed strongly between habitats, especially across the forest‐grassland transition zone (Figures S4, S5, S6; Tables S4, S5), which is visible in the thermal image (Figure 1e). PPFD, air and soil temperatures, and VPD were the highest in the grassland and considerably lower in the forested habitats, especially the canyon bottoms and upper canyon, whereas the reverse was true for air RH and soil VWC. PPFD and air and soil temperatures in the floodplain habitat were higher than in the other forested habitats, as the floodplain has a more diffuse and shorter forest canopy, but due to its proximity to the river, it had high air RH and soil VWC, and low VPD. Variation in mean microclimate was strongly influenced by topography, with significant effects of topographic PC1, PC2, and PC3, as well as month (Table S5). Even though seasonality explained more variation in microclimate (*R* ^2^ >.69 in both years), as would be expected, topography still explained almost another 10% of the variation in microclimate (Table S5).

**FIGURE 3:**
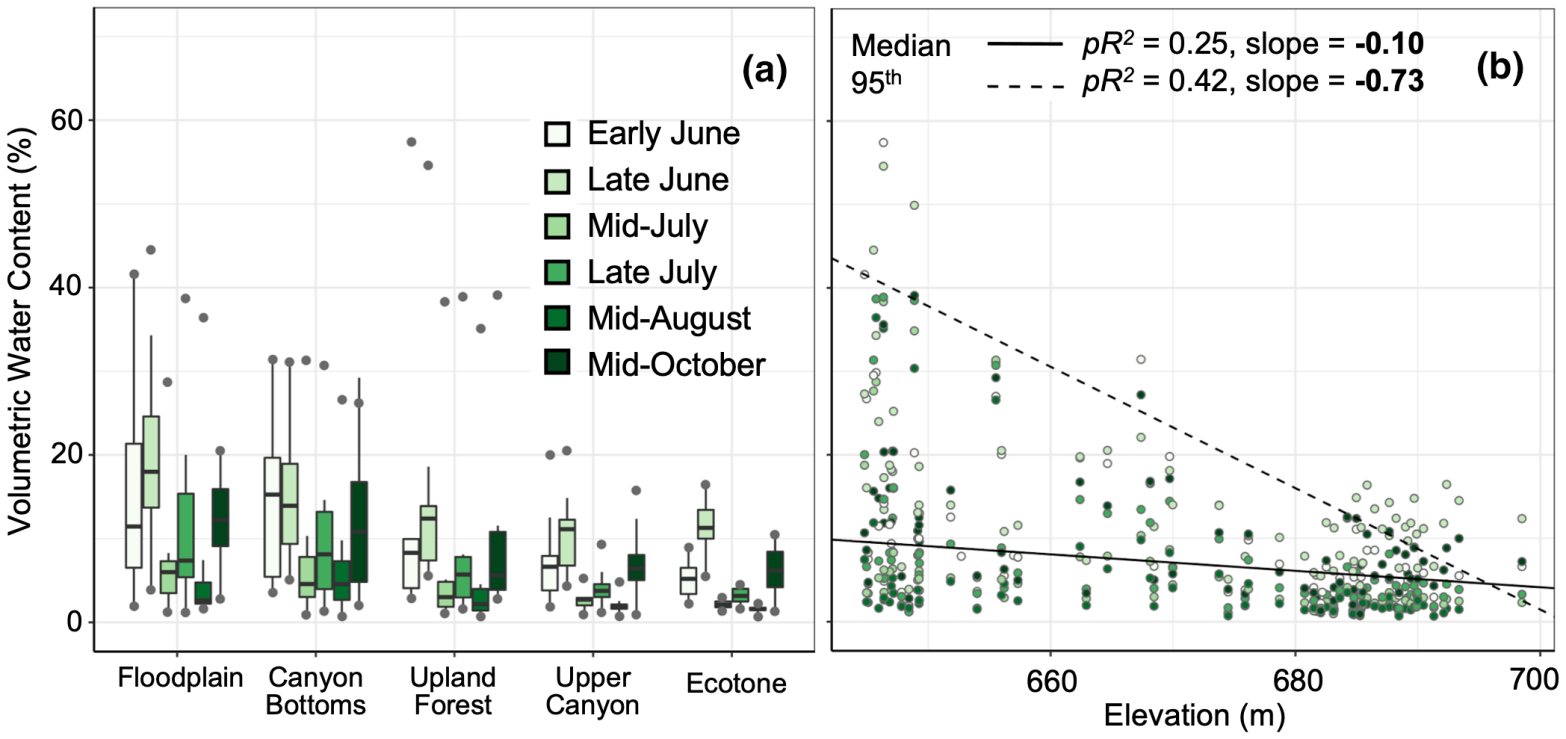
Variation in surface soil moisture during the 2021 growing season with respect to habitats and elevation. (a) Variation in soil volumetric water content (VWC) at six timepoints in five habitats, ordered by increasing elevation. (b) Scatterplot of VWC versus elevation. In (a), boxes indicate the median (center line) and first and third quartiles; lower and upper whiskers indicate the 1st and 3rd quartiles ±1.5 times the interquartile range; points indicate minimum and maximum VWC. VWC differed significantly between most timepoints in 2021 (*p ≤* .05), except for between early June and mid‐July, early June and mid‐October, mid‐July and mid‐August, and late July and mid‐October. In (b), lines are predicted values for the median (solid line) and 95th quantile (dashed line) relationships from quantile regression models. *pR* ^*2*^ is pseudo‐*R* ^*2*^; both slopes were significantly different from zero.

Between‐habitat variation in the manually measured surface soil VWC in 2021 paralleled microclimate station measurements in 2021 and 2022, creating a strong water availability gradient from the floodplain to the ecotone (Figures 3, S7). Surface soil VWC (manually measured) varied significantly among habitats (*F*_4,71_ = 2.9, *p* = .03) and time points (*F*_5,326_ = 86.7, *p* < .001; Figure 3a), but there was no significant interaction between habitat and timepoint (*F*_20,326_ = 1.3, *p* = .20). Habitat and timepoint explained 24% of the variation in VWC (marginal *pR* ^2^), but smaller‐scale differences within habitats also explained considerable variation (conditional *pR* ^2^ = .87), possibly explaining why in pairwise comparisons, only the floodplain and ecotone habitats differed significantly (*p* = .01) (Figure 3a). Soil VWC varied strongly with topography, declining with increasing elevation, slope, and solar radiation (Figures 3b, S7). For elevation, the decline was steeper for near maximum than median soil moisture (95th quantile, *pR* ^*2*^ = .42, *F*_6,420_ = 60.7, *p* < .001); median (*pR* ^*2*^ = .25, *F*_6,420_ = 54.7, *p* < .001). While lower elevations exhibited a range of VWC, higher elevations had a much narrower range of lower VWC values (Figure 3b).

Both above‐ and belowground microclimate conditions were considerably buffered in forested habitats in 2021 and 2022, with significantly lower air temperatures and lower (less negative) VPD, higher air RH, lower soil temperatures, and higher VWC in all forested habitats compared to grassland (Table S6). Buffering effects were stronger on north and east‐facing aspects than on south and west‐facing aspects and in the canyon bottoms (Table S6). Microclimate buffering was mediated by the presence of the forest overstory and topography (Table S7). Overstory basal area and both elevation and northness had significant effects on average aboveground monthly microclimate conditions in the Niobrara plot across two growing seasons (all *p ≤* .05), and each of these factors explained 4–19% of the variation in the difference in aboveground microclimate both years (Table S7). Overstory basal area, elevation, and northness also influenced belowground microclimate buffering in both years, but the effects of elevation, and of basal area in the model including northness, were not significant in 2022 (*p* > .05), and these explained less of the variation in the difference in belowground microclimate (3–10%) compared to aboveground conditions (Table S7).

### 3.2 P2 Woody species occupied distinct topographic niches corresponding to geographic distributions

**FIGURE 4:**
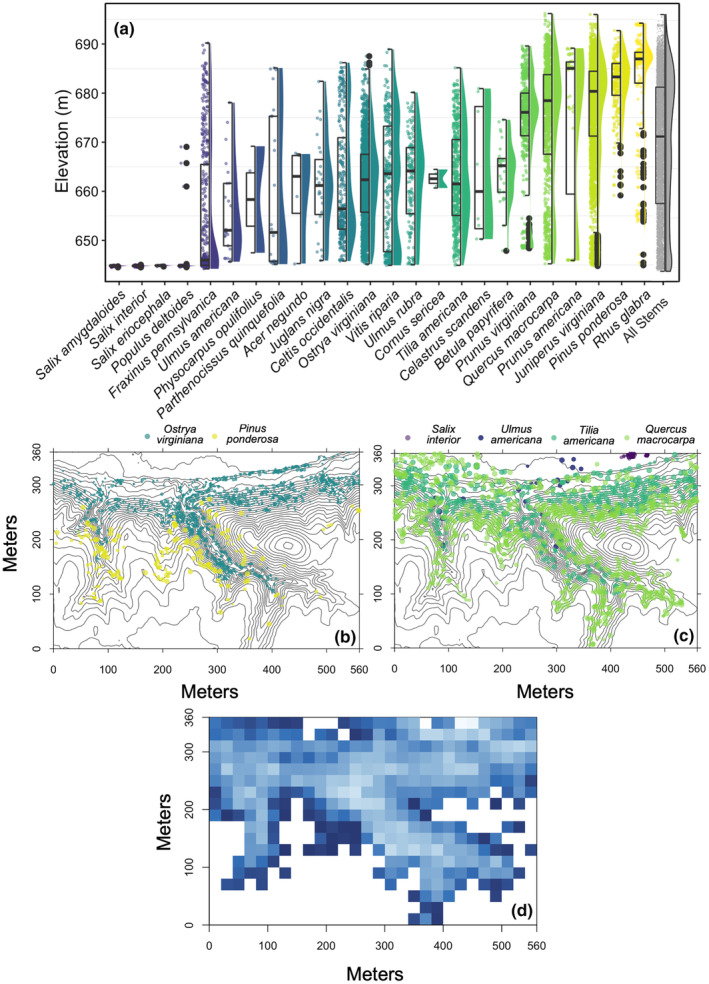
Topographic niches of woody species and variation in species composition in the Niobrara plot. (a) Combined box and violin plots showing the elevation distributions for woody species with ≥2 individuals in the plot, ordered by increasing median elevation. Boxes indicate the median (center line) and 1st and 3rd quartiles; lower and upper whiskers are 1st and 3rd quartiles ±1.5 times the interquartile range; points are outliers. (b, c) Topographic maps of the Niobrara plot with 5‐m elevation contour lines, showing example species distributions (color‐coded by median elevation) for (b) *Ostrya virginiana* and *Pinus ponderosa*, and (c) *Salix interior, Ulmus americana*, *Tilia americana*, and *Quercus macrocarpa*. See Table S8  for statistical tests of niche position and breadth. (d) Variation in species composition in forested quadrats, with similarly shaded blue squares having more similar species composition, and white squares representing quadrats with no woody stems ≥1 cm diameter at breast height. The blue color ramp is scaled to the scores from the first axis of a non‐metric multidimensional scaling (NMDS) using a Cao dissimilarity matrix of between‐quadrat differences in tree species composition at the 20 × 20 m quadrat scale. The NMDS was performed with three dimensions (*k* = 3) and a maximum of 999 iterations, and 0.143 was the lowest stress value (non‐metric *R* ^2^ = .98; linear *R* ^2^ = .87).

Topography strongly shaped woody species' distributions, and most species occupied well‐defined elevation niches (Figure 4; Table S8). Among 22 species with sufficient sample size to test, 18 (82%) exhibited significantly lower and/or narrower elevation niches than expected by chance. Eleven species (50%) had both lower and narrower elevation niches, including all willow (*Salix*) species found in the floodplain and species with geographic distributions concentrated in more mesic, easterly portions of North America (e.g., *Fraxinus pennsylvanica*, *Juglans nigra*, *Ostrya virginiana*). The elevation niches of five species (23%) were only narrower than expected by chance, including *Betula papyrifera*, with a geographic distribution concentrated in cooler, moister, more northerly regions, and *Pinus ponderosa*, with a geographic distribution concentrated in drier, more westerly regions, as well as species widely distributed in the Great Plains and Midwest (*Juniperus virginiana*, *Prunus virginiana*, *Quercus macrocarpa*). Two (18%) other widely distributed Great Plains species (*Populus deltoides*, *Parthenocissus quinquefolia*) exhibited lower, but not narrower, niches than expected by chance.

### 3.3 P3 Significant variation in forest structure, diversity, and composition among habitats and with topography

**FIGURE 5:**
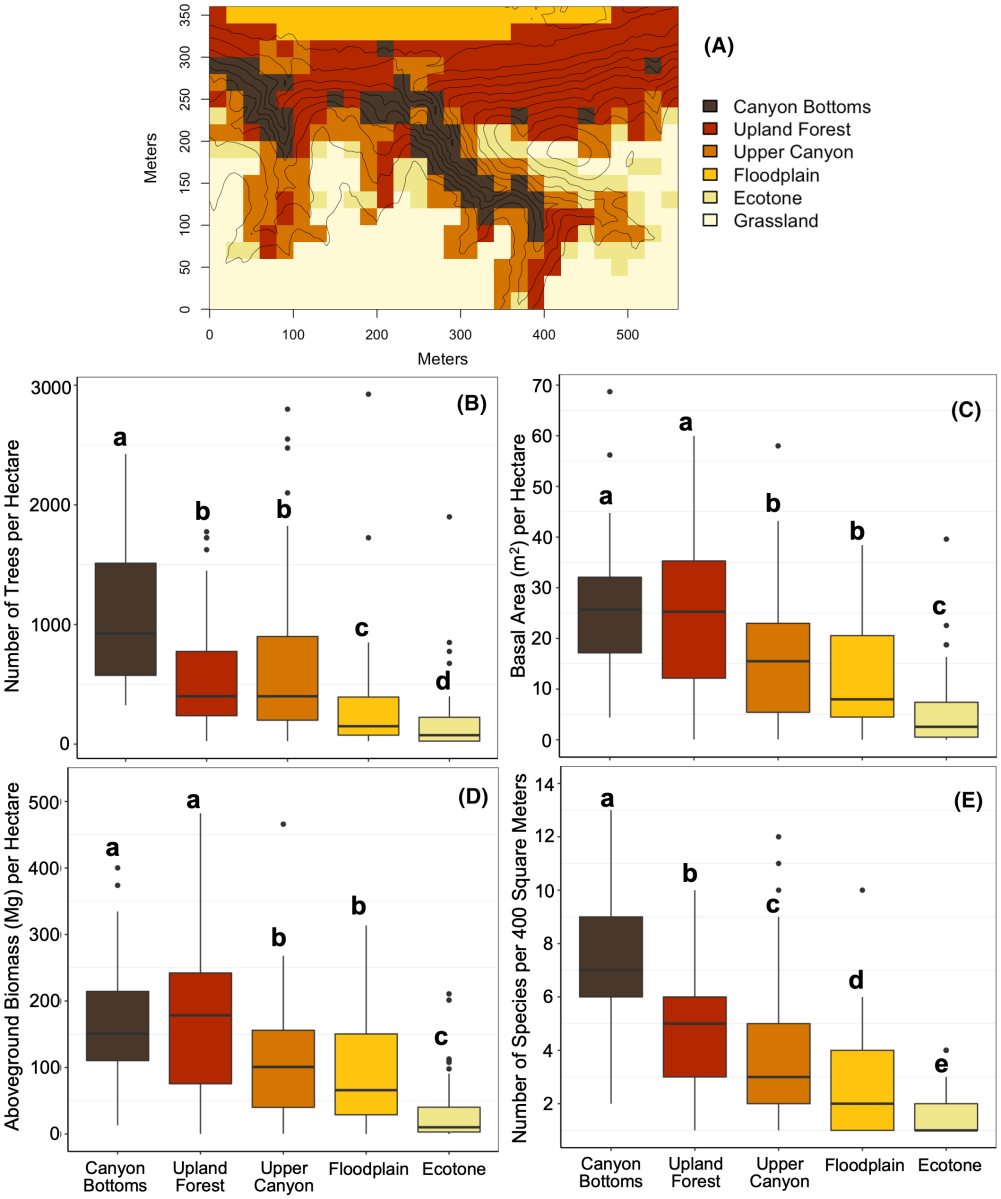
Topographic habitats and variation in forest structure and diversity among five habitats in the Niobrara plot. (a) a map of the 20 × 20 m quadrats in the Niobrara plot, color‐coded by habitat as indicated in the legend, and overlaid with 5‐m elevation contour lines. (b‐e) Boxplots showing (b) tree density per hectare, (c) basal area per hectare, (d) aboveground biomass per hectare, and (e) species richness per quadrat (400 m^2^), with habitats ordered by increasing light intensity and exposure, and grassland excluded because there are no woody stems in this habitat. Letters above the boxes indicate significant pairwise differences after correction for multiple comparisons using Tukey's Honestly Significant Differences. Boxes indicate the median (center line) and 1st and 3rd quartiles; lower and upper whiskers indicate the 1st and 3rd quartiles ±1.5 times the interquartile range; points indicate outliers. One quadrat with very high tree density was excluded from (b) for visualization; statistical analyses with and without this quadrat were consistent.

Forest structure, diversity, and composition varied among habitats (Figure 5, Table S9). Tree density, total basal area, and total AGB were significantly higher in the moister, lower‐light habitats at lower and middle elevations (canyon bottoms, upland forest, upper canyon) compared to the more exposed, higher‐light, hotter habitats (floodplain and ecotone) (Figure 5B–D). Species richness was significantly higher in habitats with more complex forest structures that had cooler, moister microclimates (canyon bottoms) as compared to higher‐light, hotter habitats (floodplain and ecotone; all habitat pairs significantly different) (Figure 5E). Shannon's diversity indices ranged from 0 (only one species present) to 2.07 per quadrat (per 400 m^2^), with higher diversity in the moister, cooler canyon bottoms than the exposed ecotone (Table S9). Species composition varied significantly among habitats (*F*_4,334_ = 16.1, *R* ^*2*^ = .16, *p* = .001), and there was greater within‐ than between‐habitat similarity in composition (*R*‐value = .27, *p* = .001). Habitats also differed in variability in composition (*F*_4,334_ = 8.2, *p* < .001).

**FIGURE 6:**
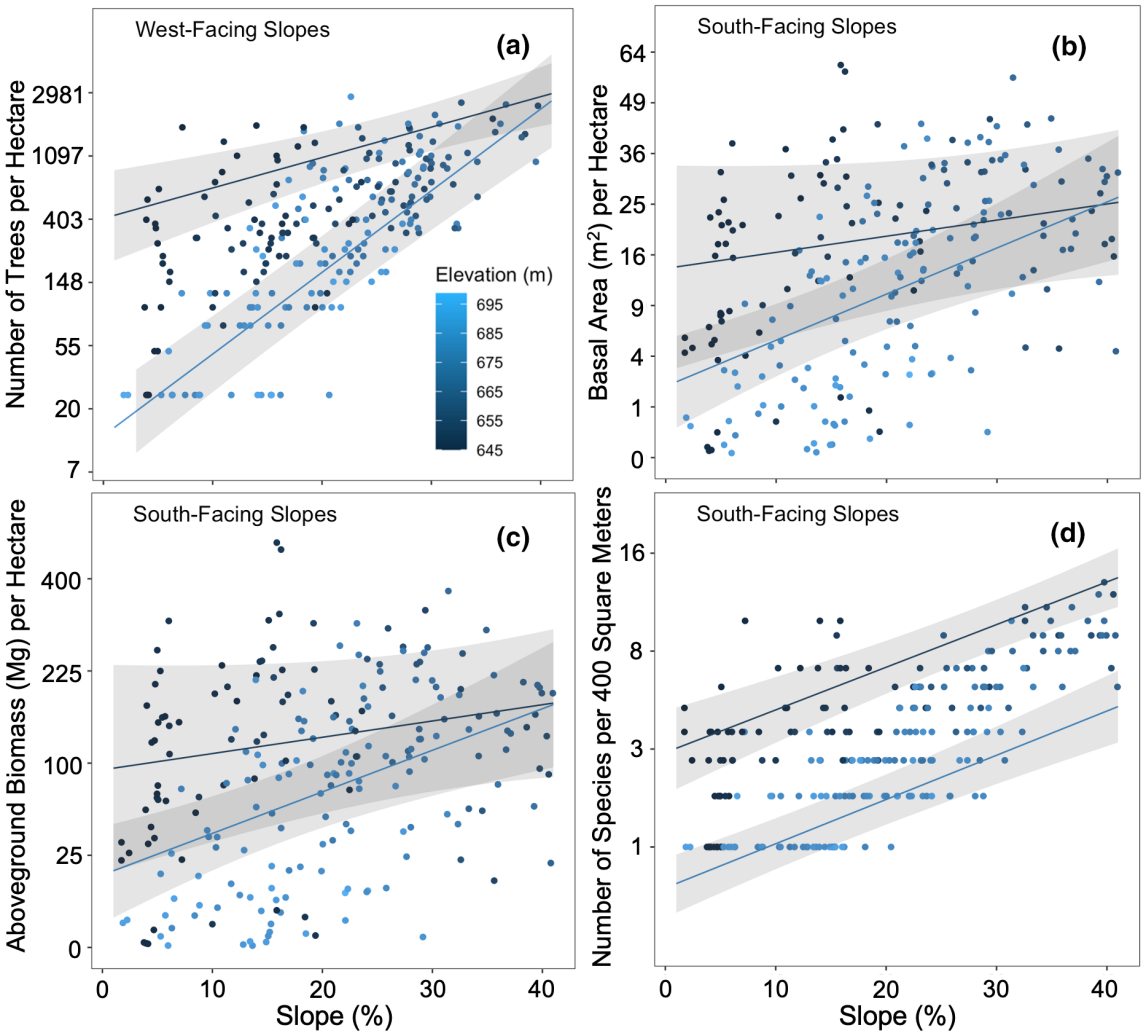
Continuous variation in forest structure and diversity with respect to topographic gradients in the Niobrara plot. Scatterplots (points) with predictions (lines and 95% confidence ribbons) based on the best candidate generalized additive models (Table S8, S10) showing variation in (a) tree density per hectare, (b) basal area per hectare, (c) aboveground biomass per hectare, and (d) species richness per quadrat (400 m^2^) versus slope (a–d). All response variables have been back‐transformed. Predictions were generated by setting elevation equal to the first and third quartiles (655 and 685 m), indicated by dark and light blue fitted lines, and specifying a (a) west‐facing or (b–d) south‐facing aspect. Each point represents a quadrat, color‐coded by elevation (in‐figure legend), corresponding to quadrats with (a) west‐facing or (b–d) south‐facing aspects.

Forest structure, diversity, and composition varied significantly with respect to continuous topographic gradients (Figures 4, 6; Tables S10, S11). Tree density increased with steepness, and this effect was stronger at lower than higher elevations and for more westerly than easterly aspects (Figure 6a). Basal area and AGB also increased with steepness, and this effect was stronger at lower elevations and for southerly than northerly aspects (Figure 6b,c). Species richness was higher in steeper but lower elevation areas, and for steepness, this effect was stronger for more northerly than southerly aspects (Figure 6d). Species composition varied significantly with topography (Figure 4d) (*R* ^2^ = .16, *p* = .001), but topographic variables differed in statistical significance and variation explained, with elevation explaining more variation (*R* ^2^ = .08, *p* = .001) than slope (*R* ^2^ = .03, *p* = .001), solar radiation (*R* ^2^ = .002, *p* = .03), and aspect (*R* ^2^ < = .001, *p* > .05).

### 3.4 P4 Significant variation in forest structure, diversity, and composition with microclimate

Forest structure, diversity, and composition depended on mean microclimate and its variability (Tables S12, S13). Tree density did not vary significantly with mean microclimate in either year (Table S12). Basal area and AGB increased significantly with increasing (less negative) VPD (i.e., were higher in less dry areas) but did not vary significantly with soil moisture nor temperature, even though models including soil moisture and temperature explained 40–75% of the variation (Table S12). Species richness varied with mean microclimate (62–70% variance explained), but only VPD was a significant factor, negatively affecting richness. Microclimate variability influenced tree density, basal area, AGB, and species richness in both years (41–47%, 0–53%, 4–69%, and 24–53% of variance explained, respectively), but effects were significant only for soil temperature, and only in 2021, for all response variables except tree density, for which no predictors were significant (Table S12).

Species composition varied significantly with microclimate and its variability (Table S13). Mean VPD significantly affected composition, explaining 25% of its variation in both years. Mean soil temperature and VWC were not significant, although together they explained 28–35% of the variation. Variability in VPD and soil temperature significantly affected composition in 2021 but not in 2022, explaining 12–30% of the variation, whereas VWC variability explained less variation (4–10%) and was not significant in either year (Table S13).

## 4 DISCUSSION

Macroclimate conditions determine the predominant biomes and vegetation distributions at large spatial scales, but finer‐scale variation in physiography can generate microclimate refugia in which local populations of species that would otherwise be ecologically filtered out of a region persist (Cartwright et al., 2020; Keppel et al., 2012; Morelli et al., 2016). In this study, topographically driven microclimate refugia embedded in a sea of semi‐arid grassland harbor forests with woody species typically found in more mesic, forested biogeographic regions of North America. Refugial populations such as these may prove to be important seed sources as the migration of trees and other forest‐dependent species follows their climatic niches (Feeley et al., 2011; Sharma et al., 2022). Moreover, because populations in refugial forests persist in a macroclimate that is at the edge of what is tolerable for many tree species, they may contain ecotypes with adaptations that could be beneficial in warmer, drier climates (Aitken et al., 2008; Valladares et al., 2014). Climate‐change refugia, such as those in our study, are therefore likely to play an increasingly important role in the conservation of biodiversity, as they have during previous periods of global climate change (Bennett et al., 1991; Keppel et al., 2012; Petit et al., 2003). Topography‐driven microclimate conditions have enabled forests to persist in these refugia across palaeoecological timescales to the present (Kaul et al., 1988). However, whether these forests will continue to serve as climate‐change refugia with suitable habitat for species currently at range edges will depend on the magnitude of future climate change in the Great Plains, where macroclimate conditions are projected to be warmer and drier (Conant et al., 2018), and conditions suitable for the persistence of temperate forest and Great Plains grasslands are expanding northward (Rehfeldt et al., 2012).

Emphasis has been placed on how a better understanding of microclimate variation can help predict responses of vegetation to climate change (De Frenne et al., 2021), but much of this work has been done in alpine systems with large elevation changes (Foster & D'Amato, 2015; Opedal et al., 2015; Rae et al., 2006). Our study demonstrated that even modest changes in topography, with its consequences for hydrology and hydrogeology, dramatically affected microclimate. The 1.9°C mean air temperature difference along the 59‐m gradient from the floodplain to the forest‐grassland transition zone is equivalent to the mean annual temperature change across approximately 2.7 degrees of latitude in North America in this region (La Sorte et al., 2014). This temperature difference along the short elevational gradient can be attributed to cooler, moister conditions in the canyons due to the presence of groundwater springs and seeps, rather than the adiabatic lapse rate. Based on remote‐sensing based estimates of soil moisture averaged across the 2021 growing season (NASA GSFC, 2020), the 8.6% mean difference in soil moisture across the elevational gradient in the Niobrara plot corresponds to the soil moisture difference at this latitude across approximately 2.1 degrees of longitude to the east (wetter) along the east‐west aridity gradient in this region. In a seasonal context, the magnitudes of these topography‐related microclimatic differences are equivalent to the change in daily regional air temperature between late June and the hottest day of the growing season in late July (based on daily averages from 1981 to 2010; U.S. Climate Data, 2022), and in soil moisture from early May to mid‐June (NASA GSFC, 2020). Thus, the magnitude of topographically driven microclimate variation can be on the order of macroclimate variation at far larger spatial and temporal scales.

Topographically driven microclimatic variation strongly affected the structure, diversity, and composition of the refugial forests in our study. Woody species exhibited well‐defined elevation niches, causing high species turnover and differences in forest composition between habitats with distinct topography and microclimate. Species' local topographic niches showed some correspondence with their geographic distributions, as has been found in a vegetation refugium in the Coast Ranges of northern California, USA (Ackerly et al., 2020). In our study, species with geographic ranges concentrated in more easterly and boreal regions of North America had narrower elevation niches corresponding to cooler moister conditions, compared with species that are more widespread in the hotter, drier regions of central and western North America. As a result, more structurally complex, diverse stands with predominantly broad‐leaved mesic forest species (*e.g.*, *Tilia americana*, *Ulmus rubra*) were found on steeper slopes in less exposed, cooler, moister habitats within the refugial forests. Conversely, less structurally complex, less diverse stands with more desiccation‐tolerant species *(e.g.*, *Q. macrocarpa*, *P. ponderosa*) were found in more exposed, hotter edges of the refugium near the forest‐grassland transition zone. While microclimate variables cumulatively explained considerable variation in forest structure, diversity, and composition, lower VPD and reduced variability in soil temperature emerged as significant factors promoting diversity and affecting species composition in this refugial forest. Many studies predicting changes in the geographic ranges of plant species based on climate suitability use climate variables gridded at scales an average of 1000 times larger than the individual plants, resulting in predictions of future range distributions that are likely to be mismatched from microclimatic conditions plants are experiencing (Dobrowski, 2011; Iverson et al., 2008; Potter et al., 2013). Even when microclimate is considered, often only temperature is measured at fine spatial scales, overlooking the key role of hydrologic processes and variation in water availability (McLaughlin et al., 2017). Our study demonstrates that topographic and climatic data at fine spatial scales are essential for identifying and characterizing climate‐change refugia where forests may persist and that consideration of multiple dimensions of climate is essential to understanding the drivers of diversity distribution within refugial forests.

### 4.1 Microclimate structures climate‐change refugia and refugial forest communities

Local microclimate conditions can differ considerably from the macroclimate (Lembrechts & Nijs, 2020), and this spatial heterogeneity in environmental conditions differentially affects species' abilities to recruit and survive (Blonder et al., 2018; Potter et al., 2013), with many woody species persisting only within climate‐change refugia in regions that are becoming warmer and drier. We found that the considerably cooler, moister, lower‐exposure conditions in the canyon bottoms and upland forest compared to adjacent grassland were strongly related to topography‐driven variation in microclimate. Although we only measured microclimatic variation in the understory, the presence of the forest overstory also contributes to this variation (De Frenne et al., 2021; Villegas et al., 2010), which we found in the Niobrara forests, with basal area significantly buffering understory microclimate. For saplings, the effects of climate warming are buffered by the overstory reducing understory temperatures and exposure (De Frenne et al., 2019; Scherrer & Körner, 2010). In the Niobrara forests, the lower light availability, cooler temperatures, lower VPD, and higher soil moisture in the understories of the forested habitats, especially those at mid‐elevations and on north and east‐facing aspects, are further evidence of such buffering. Soil moisture gradients are expected to be shaped by topography, yet few studies have quantified variation in soil moisture at fine spatiotemporal scales (Kupers et al., 2019; Ma et al., 2010; Russo et al., 2010), as we did here. Our monitoring revealed that temporal variation within habitats can be as high as soil moisture variation between habitats, possibly owing to the well‐drained soils of our study site. Soil moisture exhibited great variability at lower elevations but was consistently lower at higher elevations along the forest‐grassland transition zone. The narrow, lower‐elevation canyon bottoms were more buffered from temporal variation due to surface and groundwater flow, which moderated soil moisture and temperature, allowing the persistence of species characteristic of these mesic refugial forests (Kaul et al., 1988; Tolstead, 1942a). The current microclimate of the forests along the south side of the Niobrara River arises due to the combined effects of topography, hydrogeology, and buffering by the overstory. However, their origin and persistence, despite the increasing aridity of the macroclimate over the last ~9000 years, are primarily due to the ameliorating effects of topography and hydrogeology and are secondarily reinforced by the buffering effects of the overstory. The contrast with the north side of the Niobrara River is instructive. Its south‐facing aspect and lack of springs and seeps cause forests to be sparser and dominated by drought‐adapted species (*e.g.*, *P. ponderosa*, *Q. macrocarpa*), which are found predominantly at the forest‐grassland transition zone on the south side of the river (Kaul et al., 1988).

We expected covariation in patterns of stem density and basal area along topographic and microclimatic gradients in the Niobrara plot, and we found both to be higher in mesic, lower‐exposure areas, paralleling findings from multiple forest types (Álvarez‐Dávila et al., 2017; Smith et al., 2017). We observed that west‐facing aspects promoted higher stem density, which has been shown in other North American deciduous forests (Fekedulegn et al., 2003), whereas south‐facing aspects facilitated higher basal area, which was found in a forest with a similar macroclimate (Måren et al., 2015). Both basal area and AGB were higher in the canyon bottoms and upland forest areas that had lower mean VPD (less negative deficits), higher mean soil moisture, and greater variability in soil temperature, which suggests that the reduced dryness and presence of springs and seeps may have promoted increased stem growth and productivity. The highest AGB occurred on more moderate upland forest slopes compared to the canyon bottoms, likely driven by the high wood density of *Q. macrocarpa* (Miles & Smith, 2009), which is more abundant there (Table S1). Our results underscore the roles of elevation and aspect in creating desiccation gradients that limit woody productivity (Adams et al., 2014; Helcoski et al., 2019) and drive the rapid transition from forest refugium to grassland.

### 4.2 Topography and microclimate shape forest diversity and composition and tree species niches

Forest diversity and composition corresponded strongly to topographic and microclimatic gradients in the Niobrara forests, with differences in the breadth and position of species' topographic niches occurring due to differences in resource requirements. Tree species differ in their sensitivity to water limitation and the degree to which their growth is buffered by access to groundwater (Chitra‐Tarak et al., 2018; Costa et al., 2022; Dawson & Ehleringer, 1991), affecting species long‐term growth responses to aridity (Aus Der Au et al., 2018). In the Niobrara forests, rainfall has been hypothesized to be less critical for the survival of woody species compared to groundwater access (Kaul et al., 1988; Szilagyi et al., 2003). In a nearby tree plantation, seasonal differences were observed in soil water uptake depth by *J. virginiana* and *P. ponderosa*, with both species using deeper water during the driest part of the growing season (Eggemeyer et al., 2008). However, the deciduous woody species in the Niobrara forests are likely to be considerably less drought‐tolerant than these conifers (Abrams et al., 1998), which raises questions about future changes in forest composition driven by shifts in habitat and geographic distributions of species at their range edges in this region (Iverson et al., 2019). This is particularly true considering the increasing macroclimate aridity, the fact that more precipitation is falling in a few, large events, and potential reductions in tree access to groundwater as conversion to cropland increases regional demand for groundwater‐derived agricultural irrigation (Conant et al., 2018; Lark et al., 2020; McGuire, 2011).

Since more diverse forests provide a broader suite of ecosystem services and have increased resilience to climate extremes (Isbell et al., 2015; Reu et al., 2022), it is essential to understand how diversity is influenced by microclimate conditions. We observed more diverse stands on lower‐elevation, steeper slopes in the moister canyon bottoms and upland forest, in areas with lower mean VPD. Increased water availability and heterogeneous light conditions likely increased richness by facilitating higher co‐occurrence of species with differing requirements (Álvarez‐Dávila et al., 2017; Peterson & Reich, 2008). Our finding that species exhibit strong niche associations, with most species occupying significantly lower and/or narrower elevation niches than expected, suggests that the moister, lower‐exposure conditions in the canyon bottoms and upland forest promote higher co‐occurrence and therefore richness in these areas. Our evidence of niche partitioning parallels other studies showing that tree species can have distinct topographic niches despite little elevation change (Harms et al., 2001; Pulla et al., 2017; Zuleta et al., 2018). While strong differences between local topographic distributions of species from diverse geographic provenances may signal future changes in the diversity and composition of this climate‐change refugium and the forest‐grassland transition zone, data on growth, survival, and recruitment is required to evaluate this hypothesis.

## 5 CONCLUSIONS

Our study provides novel insights into drivers of variation in forest structure, diversity, and composition in a biogeographically important refugial forest in the North American Great Plains. Topography and the associated variation in hydrogeology and canopy cover strongly influenced microclimatic variation across small spatial scales, creating microclimate buffering within the regional semi‐arid macroclimate and supporting forests in the midst of the predominant grassland biome. The topography‐driven microclimate variation corresponded with changes in forest composition, promoting co‐existence of species with distinct topographic niches linked to their diverse geographic provenances and physiological tolerances, and driving the transition to grassland at higher elevations. As in past eras of dramatic climate change, we speculate that climate‐change refugia, such as those in the Niobrara, will be critical in maintaining biodiversity by allowing forests to persist. The refugia may ameliorate tree stress and mortality associated with larger‐scale macroclimatic changes (Suggitt et al., 2018), increasing resilience by diverting these systems from climate change‐driven tipping points that precipitate complete forest loss (Tepley et al., 2017). It is uncertain whether the Niobrara climate‐change refugia, though critical in allowing populations of woody species at their geographic range extremes to persist in the past and present, will remain refugia for these species under future macroclimate change in the Great Plains, given the uncertainty in the magnitude of change that is predicted (Conant et al., 2018). However, the transition from more mesic conditions at lower elevations to more xeric conditions at higher elevations encapsulates at a local scale the anticipated trajectory of climate change in the Niobrara region. Thus, investigating dynamics in refugial forests at biome boundaries may provide early indicators for how species in the core parts of forested biomes will respond to direct and indirect effects of climate change, particularly in climate‐change refugia sheltering forest‐dependent species at edges of their habitat, geographic, and biome ranges.

## CONFLICT OF INTEREST STATEMENT

The authors have no conflicts of interest to declare.

## Supporting information


**Data S1:** Supporting information.

## Data Availability

The Niobrara forest inventory plot data and microclimate data will be archived and made available at: https://forestgeo.si.edu/explore‐data/niobrara‐termsconditionsrequest‐forms.
