# Supplementary material for: Topography‐driven microclimate gradients shape forest structure, diversity, and composition in a temperate refugial forest
Source: Plant Environ Interact. 2024 Jun 11;5(3):e10153. doi: 10.1002/pei3.10153 (PMC11166229; doi:10.1002/pei3.10153)
Supplement: Supplementary file 1 — Data S1: Supporting information. [file PEI3-5-e10153-s001.docx]

**Supporting Information**

**Topography-driven microclimate gradients shape forest structure, diversity, and composition in a temperate refugial forest**

Bailey H. McNichol^1*^, Ran Wang^2^, Amanda Hefner^3^, Chris Helzer^3^, Sean M. McMahon^4^, and Sabrina E. Russo^1,5^

^1^School of Biological Sciences, University of Nebraska–Lincoln, Lincoln, Nebraska, USA

^2^School of Natural Resources, University of Nebraska–Lincoln, Lincoln, Nebraska, USA

^3^The Nature Conservancy, Omaha, Nebraska, USA

^4^Smithsonian Institution Forest Global Earth Observatory, Smithsonian Environmental Research Center, Edgewater, Maryland, USA

^5^Center for Plant Science Innovation, University of Nebraska–Lincoln, Lincoln, Nebraska, USA

*Corresponding author. Email: [mcnich38@msu.edu](mailto:mcnich38@msu.edu). Current Affiliation: Department of Forestry, Michigan State University.

**Methods S1**

**Materials and methods**

*Study site and census methods*

The Niobrara Forest Dynamics Plot (hereafter, Niobrara plot; 42°46’48.83” N, 100°01’15.56” W; Fig. 1) is part of the Smithsonian Forest Global Earth Observatory (ForestGEO) network of forest dynamics plots (Davies *et al.*, 2021), which quantify forest diversity and dynamics worldwide. The 20.2 ha (560 m x 360 m) Niobrara plot was established in 2019 using standard ForestGEO methods (Condit, 1998) within the Nature Conservancy’s Niobrara Valley Preserve (Johnstown, Nebraska, USA) in the North American Great Plains (Fig. 1a,b).

From 2000-2015, the average cumulative annual precipitation on the Niobrara plot was 55.6 cm, most of which occurred during the warmest part (May-August) of the growing season (April-October). The average annual temperature was 9.6 ºC, and average minimum and maximum daily temperatures were -11.3 and 32 ºC, respectively (PRISM Climate Group, 2020). This climate regime lies within the temperate grassland biome, near the boundary with the woodland/shrubland biome (Whittaker, 1975; Chapin *et al.*, 2011). Soils in the plot are entisols, which are characterized by little development of soil profile horizons and are commonly found at sites of recently deposited materials, or of parent materials resistant to weathering (*e.g.*, sand) (Soil Survey Staff, USDA NRCS, 2015). Inavale and Almeria loamy fine sands occur along the floodplain (entisols, 0-2% slopes), McKelvie-Fishberry-Rock outcrop complex occur in the forest and along the ecotone (loamy entisols typical on valley sides, 11-60% slopes), and the Valentine-Simeon complex (excessively drained sandy entisols, 9-40% slopes) are found in the grassland (Soil Survey Staff, USDA NRCS, 2019).

The forests along the south side of the Niobrara River valley, which the Niobrara plot samples, are bordered by Sandhills grassland, creating a dramatic forest-grassland transition zone (Kaul *et al.*, 1988) (Fig. 1b) with well-defined thermal environments visible at large spatial scales (Fig. 1e). The Niobrara plot encompasses a 59-m elevational gradient (644-703 m.a.s.l.) from the Niobrara River floodplain to the transition zone at higher elevations (Fig. 1b,c). Forests occur in and along the canyons and slopes draining into the Niobrara River and are supported by groundwater seeps and springs that emerge along the canyon walls (Hearty 1978) (Fig. S2), contributing to a cooler, moister microclimate (Tolstead, 1942) (Fig. 1c,d,e). As a result of these conditions, the Niobrara forests serve as microclimate refugia; many plant species’ geographic ranges extend along the Niobrara valley, causing species with western, eastern, and boreal distributions to co-occur alongside centrally distributed and widespread species (Fig. S1, Table S1).

The Niobrara plot is subdivided into a grid of 504 20 × 20 m quadrats (Fig. 1b,c) permanently marked by posts georeferenced to within *ca.* 10 cm by a professional surveyor. In the first plot census (2018-2019), within each quadrat, all woody stems (trees, shrubs, and lianas) ≥1 cm in stem diameter at breast height (1.3 m, DBH) were individually tagged, identified to species, measured for DBH, mapped using a total station (Leica Flexline TS06 Plus, Leica Geosystems AG), and georeferenced based on surveyed posts. Secondary stems (*i.e.*, multiple stems on the same individual emerging from the main stem below 1.3 m and with DBH ≥1 cm) were also tagged for better estimation of basal area and aboveground biomass (AGB). In the first census, 339 of the 504 quadrats in the plot (67%) had at least one woody stem ≥1 cm, for a total of 8,299 individuals of 27 woody species, including 25 deciduous broadleaved species (three lianas, four shrubs, and 18 trees) and two evergreen coniferous tree species (Tables S1, S2).

*Estimation of DEM-derived topographic variables*

We obtained a 1-m resolution Digital Elevation Model (DEM) derived from remotely sensed Light Detection and Ranging (LiDAR) data for Brown County, Nebraska (U.S. Geological Survey, 2017; Fig. 1c). We used the DEM encompassing the Niobrara plot to estimate the following five topographic variables: elevation (m), slope (%), aspect (degrees), and the cumulative annual solar radiation (Wh/m^2^; Fig. 1b-d) following Fu & Rich (2002) using ESRI ArcMap version 10.8 (ESRI, 2020). Aspect, a circular variable that varies from 0-360 degrees, was decomposed into two linear variables representing “northness” and “eastness” by converting to radians, and taking the cosine to derive northness (where -1 is due south, 1 is due north, and 0 represents either east or west), and the sin to derive eastness (where -1 is due west, 1 is due east, and 0 represents north or south) (Roberts, 1986; Gillingham & Parker, 2008). Mean values of each topographic variable were obtained for each quadrat by averaging the 1-m scale values from the DEM encompassed within a quadrat. The 20-m scale is appropriate for linking variation in topography with forest structure, diversity, and composition because larger scales would encompass too much topographic variation, but smaller scales would not encompass enough woody stems for robust analyses. Values for each topographic variable for every woody stem in the plot were estimated by matching the coordinates of the stem to the nearest coordinate from the 1-m scale DEM. We define topography to include physiography (*i.e.,* slope, aspect, elevation, solar radiation) and the changes in hydrological and hydrogeological features (*e.g.,* groundwater-to-surface water flow) that physiographic variation causes, which together influence variation in microclimate (Fig. 2).

*Monitoring of microclimate conditions along topographic gradients*

We quantified variation in microclimate conditions throughout the 2021 and 2022 growing seasons using ten monitoring stations deployed along the topographic gradients in the Niobrara plot from April to November each year (full leaf-out occurred in late May; Stations 1-10 in Fig. S2). At each station, understory light availability (photosynthetic photon flux density; PPFD, $\mu$mol/m^2^/s;) was measured with 1-2 quantum sensors (Li-Cor LI-190R) and air temperature (ºC) and relative humidity (RH, %) were measured at approximately 1 m height above the ground (Campbell Scientific HMP35C, CS-215, and HygroVUE5). Soil temperature (ºC) was measured at a depth of approximately 10 cm (Campbell Scientific 105E-L and 108), and surface soil moisture (volumetric water content; VWC, %) was measured with 1-2 time-domain reflectometry sensors to a depth of 30 cm (Campbell Scientific CS616). Additionally, at the station in grassland, just past the forest-grassland transition (Station 10), a tipping bucket (Texas Electronics model TR-525I) was deployed to measure rainfall (cm). Mean values over fine timescales (every 1 min for light and every 5 min for other variables) were estimated based on temporarily stored measurements every 5 s and recorded on battery-powered data loggers (Campbell Scientific models CR-800 and CR-1000).

The microclimate stations measured conditions at fine time scales, but at only ten locations in the Niobrara plot (Fig. S2). To quantify variation in surface soil moisture with greater spatial coverage along the topographic gradients, in 2021, manual point measurements of VWC (%) were taken using a hand-held time-domain reflectometer (Campbell Scientific Hydrosense II) in 73 quadrats distributed across each habitat (canyon bottoms = 15, upland forest = 14, upper canyon = 14, floodplain = 15, ecotone = 15; see Materials and methods: *Statistical analysis* for habitat definitions). Measurements at approximately the same 3 locations per quadrat were taken on a rain-free day six times between May and October 2021.

*Statistical analysis*

All analyses were conducted in R software version 4.0.2 (R Core Team, 2022).

*Derivation of habitat types*

Following previous studies (*e.g.*, Valencia *et al.*, 2004; Kenfack *et al.*, 2014), we defined five categorical topographic habitats using the five topographic variables. To capture the covariation among these variables, we conducted principal components analysis (PCA) using scaled data as implemented in the *prcomp* function in the ‘stats’ package (R Core Team 2022). The first principal component (PC1; 42.7% of variation explained) was correlated with variation in slope, solar radiation, and elevation, and PC2 (22.6% of variation explained) was correlated with aspect (Fig. S3a; Table S3). Habitats were defined based on the quadrats’ scores for PC1 and PC2 using the following cutoffs (with habitats listed in order of increasing light intensity and exposure): 1) canyon bottoms: PC1 > 1.00, PC2 < 0.20; 2) upland forest: PC1 $\geq$-0.20, PC2 $\geq$ 0.20; 3) upper canyon: -0.98 $\leq$PC1 $\leq$ 1.00, PC2 < 0.20; 4) floodplain: -0.98 $\leq$PC1 < -0.20, -0.72 $\leq$PC2 $\leq$ 1.81; 5) ecotone: PC1 < -0.98, -1.40 $\leq$PC2 $\leq$1.62. Due to some areas of the plot having considerable overlap in PCs driven by different variables (*e.g.*, floodplain and ecotone quadrats are high-exposure environments, but occur at the lowest and highest elevations, respectively), we manually reassigned 74 quadrats (14.6%) that had been assigned to illogical habitats based on their positions along the topographic gradients. Within the PCA-defined ecotone habitat, we assigned a sixth habitat, grassland, for all quadrats with zero woody stems with diameter at breast height ≥ 1 cm. The distribution of quadrats assigned to each habitat across the Niobrara plot is shown in Fig. 5a (canyon bottoms: 2.04 ha; upland forest: 5.56 ha; upper canyon: 3.56 ha; floodplain: 1.48 ha; ecotone: 1.80 ha; grassland: 5.72 ha). Habitats varied significantly in topographic variables (Fig. S3a), indicating that our habitat definitions were reasonable, as supported by a permutational multivariate analysis of variance (perMANOVA) of the Bray-Curtis distances between quadrats in the scaled topographic variables as a function of habitat (*F*_4,334_ = 117.6, *R^2^* = 0.58, *p* = 0.001) implemented with the *adonis2* function in the ‘vegan’ package (Oksanen *et al*., 2022).

*P1 There should be correlated variation in topography and microclimate, and the forest overstory and topography should have buffering effects on understory microclimate*

All analyses involving locally sampled microclimate data were conducted separately using the 2021 and 2022 data. We derived the vapor pressure deficit (VPD, kPa) for each measurement day from the air temperature and relative humidity, using air temperature to calculate the dewpoint temperature, and then calculating VPD using Tetens’ formula (Monteith & Unsworth, 2008). Daily microclimate data collected at the ten stations (Table S4) were summarized across all measurement days based on means and standard deviations (SD) using the ‘chron’ (James and Hornik, 2020), ‘dplyr’ (Wickham et al., 2022), ‘lubridate’ (Grolemund & Wickham, 2011) and ‘padr’ packages (Thoen, 2022). To examine variation in microclimate between stations, between habitat types, and across the growing season, we calculated the monthly mean values and coefficients of variation (SD/mean*100; CV) for each variable at each station. For missing monthly values at eight stations in April and May 2021 for RH, air temp, and VPD due to delayed sensor deployment, and for missing values for soil temperature at Station 2 from August-November in 2022 due to sensor error, we imputed values using the *imputePCA* function (‘missMDA’ package; Josse & Husson, 2016). We ran separate PCAs on the scaled monthly microclimate means and CVs for each station, as well as corresponding permutational multivariate analyses of variance (perMANOVA) (Anderson *et al.*, 2006) assessing the effect of habitat and the habitat-month interaction on the means and CVs using the Gower distance metric (Gower, 1971). To assess the degree to which microclimatic variation is driven by topographic variation, we extracted the first three PCs from the PCA on topographic variables that was used to define categorical habitat types and conducted perMANOVA to assess the additive effects of topographic PC1, PC2, PC3, and month on the dissimilarity (Gower) between stations in each month in their microclimate means and CVs.

To assess variation in surface soil VWC (manually measured) between habitats and across the 2021 growing season, we fit a linear mixed effects model on the manually measured soil VWC data, with habitat and sampling timepoint as fixed effects and quadrat as a random effect, using the *lme* and *anova.lme* functions (‘nlme’ package; Pinheiro *et al*., 2022). To assess goodness of fit, we calculated pseudo-*R^2^* values (*pR*^2^) to quantify variance explained by the fixed effects alone (marginal *pR*^2^) and by both the fixed and random effects (conditional *pR*^2^) using the *r.squaredGLMM* function (‘MuMIn’ package; Bartón, 2022). To determine if there were differences in VWC between habitats and timepoints, we used post-hoc comparisons of means using the *glht* function with a single-step adjustment for multiple comparisons (‘multcomp’ package; Hothorn *et al*., 2008).

To explore topographic constraints on variation in soil moisture, we fit quantile regressions (Cade & Guo, 2000) with the manually measured VWC (2021 only) as the response variable and the additive effects of sampling date and one of either elevation, slope, or solar radiation as predictors in three separate models. Quantile regression weights observations depending on the quantile of the response variable and is useful when data show boundary-type relationships (Koenker & Bassett, 1978; Koenker & Hallock, 2001). The quantile regressions were implemented using the *rq* function with $\tau$ = 0.5 (median quantile) and $\tau$ = 0.95 (95^th^ quantile) using the Barrodale and Roberts algorithm (‘quantreg’ package; Koenker, 2022). To assess goodness of fit of these models, we calculated *pR*^2^ values (Koenker & Machado, 1999).

To assess effects of forest overstory and topography on microclimate buffering, we calculated daily differences in average air temperature, soil temperature, VWC, RH, and VPD between each of nine microclimate stations in the forest understory (Stations 1-9) versus the station in grassland (Station 10) and averaged differences within months (April-November). We calculated the total basal area in a 5-m radius of each station and determined station elevations and northness from the DEM. We evaluated above and belowground microclimate buffering using differences in air temperature, RH, and VPD, and in soil temperature and VWC, respectively. We ran separate perMANOVAs for each year to test the effects of overstory (basal area) and topography (elevation or northness, which were included in separate models due to the modest number of stations) on microclimate buffering, where the response variable was a matrix of monthly mean above- or belowground microclimate differences between forested stations and the grassland station. To assess whether there was significant microclimate buffering within forested habitats, we ran paired t-tests between mean monthly microclimate conditions at each forested station versus the grassland station (‘stats’ package; R Core Team 2022).

*P2 Woody species should exhibit distinct topographic niches*

We evaluated whether species occupy distinct elevation niches based on whether their mean niche position and niche breadth differed from those expected by chance alone, accounting for the distribution of available elevations in forested parts of the Niobrara plot. We used conditional torus translation (Abiem *et al.*, 2020) to translate trees of each species within each quadrat to new forested quadrats, maintaining the number of quadrats occupied by the species and preserving the effects of dispersal limitation by maintaining species’ abundances and the spatial arrangement of individuals within the quadrat. The translation is conditional because trees could not be translated to grassland quadrats with no woody stems. For each species with ≥3 individuals in the Niobrara plot, we generated 999 translations. For the observed and translated distributions, the elevation of each tree was determined as described above, and the conditional probability of a tree occurring at each elevation was estimated (Itoh *et al.*, 2010). We tested whether species had distinct niche positions and narrower niches given the available elevations in the Niobrara plot than expected by chance using two-tailed and one-tailed tests based on the rank of species observed versus translated mean and standard deviation elevations, respectively. To visualize the distribution of species’ elevational niches, we assessed the density of individuals and outliers beyond the first and third quartiles ±1.5 times the interquartile range for each species.

*P3 Topography should correlate with forest structure, diversity, and composition.*

To quantify metrics of forest structure and diversity, we calculated tree density, total basal area, total AGB, and species richness and diversity for trees in each quadrat using functions in the ‘fgeo’ (Lepore *et al*., 2019), ‘allodb’ (Gonzalez‐Akre *et al*., 2022), and ‘vegan’ (Oksanen *et al*., 2022) packages. Tree density was the number of individuals per quadrat. Several species often form individuals with multiple large stems, so calculations of total basal area and AGB included multiple stems (i.e., all stems of an individual). Total quadrat basal area (m^2^) was calculated from DBH (cm) as $\Sigma\pi\times$ (DBH)^2^/(4$\times$10,000 cm/m^2^). The ‘allodb’ package uses a database of species and genus-specific allometric equations and wood densities, combined with DBH measurements, to estimate the AGB of all stems of an individual in kg (Gonzalez‐Akre *et al*., 2022), which we converted to Mg and summed for trees in each quadrat to obtain the quadrat-level AGB. We scaled tree density, basal area, and AGB to a per ha basis. We calculated woody species richness (number of species per quadrat, or per 400 m^2^) and Shannon’s diversity as *H* = $-\Sigma p_{i}\times ln(p_{i})$, where $p_{i}$is the proportion of individuals in the *i*th species (Magurran, 2004). Analyses included quadrats with at least one woody individual with DBH ≥1 cm (*N*=339).

We used two modeling approaches to quantify topography-driven variation in forest structure and diversity. First, we used linear models with habitat type as a categorical fixed-effect predictor fit using ordinary least squares regression and quadrat as the unit of replication. Box Cox transformations were applied to response variables to meet assumptions of normality v (Venables & Ripley, 2002). When there was a significant omnibus test for habitat, pairwise differences between habitats were evaluated via post-hoc multiple comparisons using Tukey’s Honestly Significant Differences (HSD) with a 5% family-wise error rate, using the *TukeyHSD* function.

Second, we used generalized additive models (GAM) with the original topographic variables as continuous predictors to assess their main and interactive effects on tree structure and diversity, with quadrat as the unit of replication. GAMs are a non-parametric extension of linear regression that apply an additive smoothing function to the predictor variables and take the following form: $g\left( E(Y) \right)=b+s_{1}\left( X_{1} \right)+\ldots+s_{p}\left( X_{p} \right)$, where *E(Y)* is the response variable, *g(Y)* is the link function, *b* is the intercept, *s(X)* is the smoothing function of each of the predictor variables, and *X_p_* are the *p* continuous predictor variables (Hastie & Tibshirani, 1987). We used GAMs because they flexibly accommodate nonlinear relationships, while also avoiding model overfitting by penalizing the smoothing parameter (Yee & Mitchell, 1991). To account for spatial autocorrelation identified in exploratory analysis (assessed using *augment* in the ‘broom’ package; Robinson et al., 2022), we included a two-dimensional thin plate spline of the quadrat coordinates in each GAM (Hutchinson & Gessler, 1994). We fit GAMs with a Gaussian error distribution and identity link function using the *gam* function (‘mgcv’ package; Wood, 2011). Elevation was standardized by subtracting the minimum elevation from each value.

We used model selection to find the most-supported model among 59 candidate GAMs fitted for each response variable. No three-way interactions between topographic variables were significant, so the most complex model (global model) included all two-way interactions between the five topographic variables, and the simplest (null model) was the intercept only model. There was no evidence of multicollinearity between topographic variables (all variance inflation factors in the global model were < 2.5). Box Cox transformations were applied to meet assumptions of normality. We performed model selection using Akaike’s Information Criterion (AIC) and determined the relative likelihood of candidate models using Akaike weights (Akaike, 1973; Burnham & Anderson, 2002), using the *map* and *map_dbl* functions (‘purrr’ package; Henry & Wickham, 2020) and the *pander* function (‘pander’ package; Daróczi & Tsegelskyi, 2022) to compare models. We plotted model predictions for the most-supported model, setting predictors not plotted on the x-axis at either their first and third quartile values (elevation) or their minimum value (northness and eastness).

We quantified variation in tree composition with respect to both categorical habitat types and continuous topographic variables. We fit a perMANOVA using Cao dissimilarity (Cao *et al.*, 1997) between quadrat pairs as the response variable and habitat type as the predictor. To test whether there was greater compositional overlap within habitats, we used analysis of similarity on quadrat-level Cao dissimilarity using the *anosim* function in ‘vegan’. To test whether habitats differed in their variance in species composition, we calculated the within-habitat multivariate dispersion for each habitat using the *betadisper* function in ‘vegan’ and compared it between habitats using the *anova* function with post-hoc multiple comparisons (Tukey’s HSD).

To assess variation in community composition between quadrats along the topographic gradients, we used non-metric multidimensional scaling (NMDS) analyses on the between-quadrat dissimilarities (Cao) with three dimensions (k = 3) and a maximum of 999 iterations, using the *metaMDS* function in ‘vegan’. To test the influence of geographic distance on species composition, we conducted multiple regression with matrices (MRM) using the *MRM* function (‘ecodist’ package; Goslee & Urban, 2007) with Cao dissimilarity in composition as the response variable and geographic distances between each quadrat pair as the predictor. Since geographic distance only explained 2.0% of the variation, we excluded this variable from further analyses of composition. To assess the effects of topography on composition, we conducted MRM with the between-quadrat Gower dissimilarities of all topographic variables as predictors and the Cao dissimilarities in composition as the response variable. Topographic variables were scaled by dividing by the standard deviation. To partition variance explained by each topographic variable, we estimated adjusted *R*^2^ from a linear model with the Gower dissimilarities in all topographic variables as predictors (full model) and linear models excluding each one of the variables, and then subtracted their adjusted *R*^2^ values from that of the full model (Swenson, 2014).

*P4 Forest structure, diversity, and composition should be correlated with microclimate variation.*

Using data collected in 2021 and 2022 in separate models, we directly analyzed the relationship between variation in microclimate and forest structure and diversity using linear models, and between microclimate variation and species composition using perMANOVA. To quantify metrics of forest structure, diversity, and composition of trees near each of the nine forested microclimate stations (excluding Station 10 in grassland), we defined a 10-m radius around each station, ensuring the entire area was within the habitat type sampled by that station. Within this radius, we determined the total number of trees, basal area, AGB, and the number of species, and using a species matrix, estimated Cao dissimilarities in species composition between pairs of areas. We calculated station-level microclimate means and CVs (one value corresponding to each station) by averaging across monthly values for VPD and soil temperature and moisture (scaled by dividing by the SD). Due to the limited number of stations, we elected to include VPD, but not air temperature nor RH, in analyses, as VPD is derived from and hence captures variation in these two variables, as evidenced by the strong correlation of VPD with RH (*r* = -0.99 in 2021; *r* = -0.98 in 2022) and air temperature (*r* = 0.88 in 2021; *r* = 0.95 in 2022). We used separate linear models to determine the effects of the microclimate means and CVs on forest structure (tree density, basal area, and AGB) and richness. We used separate perMANOVAs to assess effects of microclimate means and CVs on dissimilarities in species composition.

**References**

**Abiem I, Arellano G, Kenfack D, Chapman H. 2020.** Afromontane Forest Diversity and the Role of Grassland-Forest Transition in Tree Species Distribution. *Diversity* **12**: 30.

**Akaike H. 1973.** Information theory and an extension of the maximum likelihood principle. *In*: Petrov BN, Csaki F, Eds., International Symposium on Information Theory: 267-281.

**Anderson MJ, Ellingsen KE, McArdle BH. 2006.** Multivariate dispersion as a measure of beta diversity. *Ecology Letters* **9**: 683–693.

**Bartón K. 2022.** *Package “MuMIn.”* V 1.47.0. https://cran.rproject.org/web/packages/ MuMIn/MuMIn.pdf

**Burnham KP, Anderson DR. 2002.** Model selection and multimodel inference: a practical information-theoretic approach. 2nd ed. New York, NY: Springer.

**Cade BS, Guo Q. 2000.** Estimating Effects of Constraints on Plant Performance with Regression Quantiles. *Oikos* **91**: 245–254.

**Cao Y, Williams WP, Bark AW. 1997.** Similarity measure bias in river benthic Aufwuchs community analysis. *Water Environment Research* **69**: 95–106.

**Chapin FS, III, Matson PA, Vitousek PM. 2011.** Principles of terrestrial ecosystem ecology. 2^nd^ edition. Springer. New York. 536 pp.

**Condit R. 1998.** *Tropical Forest Census Plots*. Berlin, Heidelberg: Springer Berlin Heidelberg.

**Daróczi G, Tsegelskyi R. 2021.** pander: An R 'pandoc' writer. R package version 0.6.5. https://CRAN.R-project.org/package=pander

**Davies SJ, Abiem I, Abu Salim K, Aguilar S, Allen D, Alonso A, Anderson-Teixeira K, Andrade A, Arellano G, Ashton PS, *et al.* 2021.** ForestGEO: Understanding forest diversity and dynamics through a global observatory network. *Biological Conservation* **253**: 108907.

**Esri Inc. 2022.** ArcGIS Pro (Version 2.5). Esri Inc.

**Fu P, Rich PM. 2002.** A geometric solar radiation model with applications in agriculture and forestry. *Computers and Electronics in Agriculture* **37**: 25–35.

**Gillingham MP, Parker KL. 2008.** Differential habitat selection by moose and elk in the Besa-Prophet area of Northern British Columbia. *Alces* **44**: 23.

**Goslee SC, Urban DL. 2007.** The ecodist package for dissimilarity-based analysis of ecological data. *Journal of Statistical Software* 22(7): 1-19.

**Gonzalez-Akre E, Piponiot C, Lepore M, Anderson-Teixeira K. 2022.** allodb: Tree biomass estimation at extra-tropical forest plots. <https://github.com/ropensci/allodb>.

**Gower JC. 1971.** A general coefficient of similarity and some of its properties. *Biometrics* **27**: 623-637.

**Grolemund G, Wickham H. 2011.** Dates and times made easy with lubridate. *Journal of Statistical Software* **40**: 1-25.

**Hastie T, Tibshirani R. 1987.** Generalized additive models: some applications. *Journal of the American Statistical Association* **82**: 371-386.

**Henry L, Wickham H. 2020.** purrr: Functional programming tools. R package version 0.3.4. https://CRAN.R-project.org/package=purrr

**Hothorn T, Bretz F, Westfall P. 2008.** Simultaneous inference in general parametric models. *Biometrical Journal* **50**: 346–363.

**Hutchinson MF, Gessler PE. 1994.** Splines – more than just a smooth interpolator. *Geoderma* **62**: 45-67.

**Itoh A, Ohkubo T, Nanami S, Tan S, Yamakura T. 2010.** Comparison of statistical tests for habitat associations in tropical forests: A case study of sympatric dipterocarp trees in a Bornean forest. *Forest Ecology and Management* **259**: 323–332.

**James D, Hornik K. 2022.** chron: Chronological objects which can handle dates and times. R package version 2.3-58. https://CRAN.R-project.org/package=chron

**Josse J, Husson F. 2016.** missMDA: A package for handling missing values in multivariate data analysis. *Journal of Statistical Software* **70**: 1-31.

**Kaul RB, Kantak GE, Churchill SP. 1988.** The Niobrara River Valley, a Postglacial Migration Corridor and Refugium of Forest Plants and Animals in the Grasslands of Central North America. *Botanical Review* **54**: 44–81.

**Kenfack D, Chuyong GB, Condit R, Russo SE, Thomas DW. 2014.** Demographic variation and habitat specialization of tree species in a diverse tropical forest of Cameroon. *Forest Ecosystems* **1**: 22.

**Koenker R, Bassett G. 1978.** Regression Quantiles. *Econometrica* **46**: 33.

**Koenker R, Hallock KF. 2001.** Quantile Regression. *Journal of Economic Perspectives* **15**: 153–156.

**Koenker R, Machado JAF. 1999.** Goodness of Fit and Related Inference Processes for Quantile Regression. *Journal of the American Statistical Association* **94**: 1296–1310.

**Koenker R. 2022.** quantreg: Quantile regression. R package version 5.94. <https://CRAN.Rproject.org/package=quantreg>

**Lepore M, Arellano G, Condit R, Davies S, Detto M, Gonzalez-Akre E, Hall P, Harms K, Herrmann V, Hogan A, *et al*. 2019.** fgeo: Analyze forest diversity and dynamics. R package version 1.1.4. https://CRAN.R-project.org/package=fgeo

**Magurran AE. 2004.** *Measuring Biological Diversity*. Malden, MA: Blackwell Publishing Ltd.

**Monteith JL, Unsworth MH. 2008.** Principles of Environmental Physics. 3^rd^ Edition. New York, NY: Academic Press.

**Oksanen J, Simpson GL, Blanchet FG, Kindt R, Legendre P, Minchin PR, O’Hara RB, Solymos P, Stevens MHH, Szoecs E, *et al*. 2022.** vegan: Community ecology package. R package version 2.6-2. https://CRAN.R-project.org/package=vegan.

**Pinheiro J, Bates D, R Core Team. 2022.** nlme: linear and nonlinear mixed effects models. R package version 3.1-157. https://CRAN.R-project.org/package=nlme>.

**PRISM Climate Group. 2020.** Oregon State University. https://prism.oregonstate.edu. Data accessed and downloaded via NSF #DEB-1353301.

**R Core Team. 2022.** R: A language and environment for statistical computing. R Foundation for Statistical Computing, Vienna, Austria. https://www.R-project.org/.

**Roberts DW. 1986.** Ordination on the basis of fuzzy set theory. *Vegetatio* **66**: 123–131.

**Robinson D, Hayes A, Couch S. 2022.** broom: Convert statistical objects into tidy tibbles. R package version 1.0.1. https://CRAN.R-project.org/package=broom

**Soil Survey Staff. 2015.** Illustrated guide to soil taxonomy, version 2. U.S. Department of Agriculture, Natural Resources Conservation Service, National Soil Survey Center, Lincoln, Nebraska. 681 pp.

**Soil Survey Staff. 2019.** Custom soil resource report for Brown County, Nebraska. U.S. Department of Agriculture, Natural Resources Conservation Service. Web.

**Swenson NG. 2014.** *Functional and Phylogenetic Ecology in R*. New York, NY: Springer.

**Thoen E. 2022.** padr: Quickly get datetime data ready for analysis. R package version 0.6.1. https://CRAN.R-project.org/package=padr

**Tolstead WL. 1942.** Vegetation of the Northern Part of Cherry County, Nebraska. *Ecological Monographs* **12**: 255–292.

**U.S. Geological Survey. 2017.** 1-meter Digital Elevation Models (DEMs) - USGS National Map 3DEP Downloadable Data Collection: U.S. Geological Survey.

**Valencia R, Foster RB, Villa G, Svenning J-C, Hernández C, Romoleroux K, Losos E, Magård E, Balslev H. 2004.** Tree species distributions and local habitat variation in the Amazon: large forest plot in eastern Ecuador. *Journal of Ecology* **92**: 214–229.

**Venables WN, Ripley BD. 2002.** Modern Applied Statistics with S. 4^th^ edition. New York, NY. Springer.

**Whittaker RH. 1975.** *Communities and ecosystems*. New York, NY: MacMillan Publishing.

**Wickham H, François R, Henry L, Müller K. 2022.** dplyr: A grammar of data manipulation. R package version 1.0.10. <https://CRAN.R-project.org/package=dplyr>

**Wood SN. 2011.** Fast stable restricted maximum likelihood and marginal likelihood estimation of semiparametric generalized linear models. *Journal of the Royal Statistical Society (B)* **73**: 3–36.

**Yee TW, Mitchell ND. 1991.** Generalized additive models in plant ecology. *Journal of Vegetation Science* **2**: 587–602.

Table S1. Taxonomy, growth form, and habitat associations for the 27 woody species reaching diameter at breast height (DBH) ≥1 cm in the 20.2 ha Niobrara plot in refugial forest on the south side of the Niobrara River, Nebraska, USA. Geographic range descriptions are qualitative, based on visual inspections of the distributions documented from aggregated journals and periodicals, monographs, and herbarium records by the Biota of North America Program. Stem density is the number of stems per hectare (ha) in the habitat of greatest stem density, and densities are standardized by habitat area (*i.e.*, divided by total ha of all quadrats in that habitat that have ≥1 individual; canyon bottoms: 2.04 ha; upland forest: 5.56 ha; upper canyon: 3.56 ha; floodplain: 1.16 ha; ecotone: 0.6 ha).

| **Scientific name (Family)** | **Growth form** | **Geographic range** | **Stem density (stems/ha)** | **Habitat of greatest stem density** |
| --- | --- | --- | --- | --- |
| *Acer negundo* L. (Sapindaceae) | Canopy tree | Widespread | 1.47 | Canyon bottoms |
| *Betula papyrifera* Marshall (Betulaceae) | Canopy tree | Boreal | 8.82 | Canyon bottoms |
| *Celastrus scandens* L. (Celastraceae) | Liana | Eastern | 2.94 | Canyon bottoms |
| *Celtis occidentalis* L. (Cannabaceae) | Canopy tree | Eastern | 22.66 | Upland forest |
| *Cornus sericea* L. (Cornaceae) | Shrub | Widespread | 0.98 | Canyon bottoms |
| *Fraxinus pennsylvanica* Marshall (Oleaceae) | Canopy tree | Widespread | 171.55 | Floodplain |
| *Juglans nigra* L. (Juglandaceae) | Canopy tree | Eastern | 3.42 | Upland forest |
| *Juniperus virginiana* L. (Cupressaceae) | Canopy tree | Eastern | 286.52 | Upper canyon |
| *Morus alba* L. (Moraceae) | Canopy tree | Non-native | 0.49 | Canyon bottoms |
| *Ostrya virginiana* (Mill.) K. Koch (Betulaceae) | Understory tree | Eastern | 142.63 | Upland forest |
| *Parthenocissus quinquefolia* L. Planch. (Vitaceae) | Liana | Eastern | 7.76 | Floodplain |
| *Physocarpus opulifolius* (L.) Maxim. (Rosaceae) | Shrub | Eastern | 0.49, 0.18 | Canyon bottoms & Upland forest |
| *Pinus ponderosa* Lawson & C. Lawson (Pinaceae) | Canopy tree | Western | 24.44 | Upper canyon |
| *Populus deltoides* W. Bartram ex Marshall (Salicaceae) | Canopy tree | Widespread | 158.62 | Floodplain |
| *Prunus americana* Marshall (Rosaceae) | Treelet | Widespread | 43.33 | Ecotone |
| *Prunus virginiana* L. (Rosaceae) | Treelet | Widespread | 106.86 | Canyon bottoms |
| *Quercus macrocarpa* Michx. (Fagaceae) | Canopy tree | Central | 69.42 | Upland forest |
| *Rhus aromatica* Aiton (Anacardiaceae) | Clonal shrub | Widespread | 0.49 | Canyon bottoms |
| *Rhus glabra* L. (Anacardiaceae) | Clonal shrub | Widespread | 30.90 | Upper canyon |
| *Salix amygdaloides* Andersson (Salicaceae) | Treelet | Widespread | 39.66 | Floodplain |
| *Salix eriocephala* Michx. (Salicaceae) | Treelet | Eastern | 10.34 | Floodplain |
| *Salix interior* Rowlee (Salicaceae) | Treelet | Central | 50.00 | Floodplain |
| *Tilia americana* L. (Malvaceae) | Canopy tree | Eastern | 51.08 | Upland forest |
| *Ulmus americana* L. (Ulmaceae) | Canopy tree | Eastern | 1.98 | Upland forest |
| *Ulmus laevis* Pall. (Ulmaceae) | Canopy tree | Non-native | 0.28 | Upper canyon |
| *Ulmus rubra* Muhl. (Ulmaceae) | Canopy tree | Eastern | 11.15 | Upland forest |
| *Vitis riparia* Michx. (Vitaceae) | Liana | Eastern | 43.14, 15.83 | Canyon bottoms & Upland forest |

Table S2. Abundance of the 27 woody species in five habitats in the Niobrara plot. Habitats are ordered by increasing exposure, the habitat with the highest combined abundance of adults and saplings per species is shown in bold, and the diameter at breast height (DBH, cm) range represented by individuals in each size class is shown for trees with DBH $\boldsymbol{\geq}$1 cm.

| **Species** | **Size class** | **Canyon bottoms** | **Upland forest** | **Upper canyon** | **Flood-plain** | **Ecotone** | **DBH range** |
| --- | --- | --- | --- | --- | --- | --- | --- |
| *Acer negundo* | Total | **3** | 0 | 0 | 1 | 0 | 1.0-35.6 |
|  | Adult | **2** | 0 | 0 | 0 | 0 | 11.9-35.6 |
|  | Sapling | **1** | 0 | 0 | 1 | 0 | 1.0-3.0 |
| *Betula papyrifera* | Total | **18** | 7 | 3 | 0 | 0 | 1.5-39.4 |
|  | Adult | **15** | 7 | 3 | 0 | 0 | 5.4-39.4 |
|  | Sapling | **3** | 0 | 0 | 0 | 0 | 1.5-5.0 |
| *Celastrus* | Total | **6** | 2 | 0 | 0 | 0 | 1.0-1.5 |
| *scandens* | Adult | **0** | 0 | 0 | 0 | 0 | NA |
|  | Sapling | **6** | 2 | 0 | 0 | 0 | 1.0-1.5 |
| *Celtis* | Total | 105 | **126** | 36 | 10 | 0 | 1.0-61.0 |
| *occidentalis* | Adult | 36 | **85** | 17 | 5 | 0 | 5.2-61.0 |
|  | Sapling | 69 | **41** | 19 | 5 | 0 | 1.0-4.9 |
| *Cornus sericea* | Total | **2** | 0 | 0 | 0 | 0 | 1.0-1.0 |
|  | Adult | **0** | 0 | 0 | 0 | 0 | NA |
|  | Sapling | **2** | 0 | 0 | 0 | 0 | 1.0-1.0 |
| *Fraxinus* | Total | 67 | 100 | 41 | **199** | 3 | 1.0-80.1 |
| *pennsylvanica* | Adult | 37 | 81 | 27 | **110** | 1 | 5.1-80.1 |
|  | Sapling | 30 | 19 | 14 | **89** | 2 | 1.0-4.9 |
| *Juglans nigra* | Total | 7 | **19** | 1 | 0 | 0 | 2.0-74.0 |
|  | Adult | 4 | **16** | 1 | 0 | 0 | 5.9-74.0 |
|  | Sapling | 3 | **3** | 0 | 0 | 0 | 2.0-3.8 |
| *Juniperus* | Total | 714 | 901 | **1020** | 7 | 188 | 1.0-68.6 |
| *virginiana* | Adult | 458 | 647 | **573** | 5 | 126 | 5.1-68.6 |
|  | Sapling | 256 | 254 | **447** | 2 | 62 | 1.0-5.0 |
| *Morus alba* | Total | **1** | 0 | 0 | 0 | 0 | 14.0* |
|  | Adult | **1** | 0 | 0 | 0 | 0 | 14.0* |
|  | Sapling | **0** | 0 | 0 | 0 | 0 | NA |
| *Ostrya* | Total | 576 | **793** | 192 | 8 | 0 | 1.0-17.6 |
| *virginiana* | Adult | 287 | **439** | 90 | 4 | 0 | 5.1-17.6 |
|  | Sapling | 289 | **354** | 102 | 4 | 0 | 1.0-5.0 |
| *Parthenocissus* | Total | 5 | 6 | 4 | **9** | 0 | 1.0-3.4 |
| *quinquefolia* | Adult | 0 | 0 | 0 | **0** | 0 | NA |
|  | Sapling | 5 | 6 | 4 | **9** | 0 | 1.0-3.4 |
| *Physocarpus* | Total | **1** | **1** | 0 | 0 | 0 | 1.3-1.6 |
| *opulifolius* | Adult | **0** | **0** | 0 | 0 | 0 | NA |
|  | Sapling | **1** | **1** | 0 | 0 | 0 | 1.3-1.6 |
| *Pinus ponderosa* | Total | 44 | 63 | **87** | 0 | 25 | 1.0-67.9 |
|  | Adult | 41 | 55 | **78** | 0 | 24 | 5.3-67.9 |
|  | Sapling | 3 | 8 | **9** | 0 | 1 | 1.0-5.0 |
| *Populus deltoides* | Total | 1 | 3 | 0 | **184** | 0 | 1.0-85.5 |
|  | Adult | 1 | 3 | 0 | **2** | 0 | 5.2-85.5 |
|  | Sapling | 0 | 0 | 0 | **182** | 0 | 1.0-3.8 |
| *Prunus* | Total | 0 | 1 | 8 | 12 | **26** | 1.0-4.8 |
| *americana* | Adult | 0 | 0 | 0 | 0 | **0** | NA |
|  | Sapling | 0 | 1 | 8 | 12 | **26** | 1.0-4.8 |
| *Prunus virginiana* | Total | **218** | 106 | 197 | 0 | 0 | 1.0-5.7 |
|  | Adult | **2** | 0 | 0 | 0 | 0 | 5.7-5.7 |
|  | Sapling | **216** | 106 | 197 | 0 | 0 | 1.0-5.0 |
| *Quercus* | Total | 142 | **386** | 305 | 44 | 62 | 1.1-67.3 |
| *macrocarpa* | Adult | 129 | **370** | 283 | 37 | 54 | 5.1-67.3 |
|  | Sapling | 13 | **16** | 22 | 7 | 8 | 1.1-4.9 |
| *Rhus aromatica* | Total | **1** | 0 | 0 | 0 | 0 | 1.1* |
|  | Adult | **0** | 0 | 0 | 0 | 0 | NA |
|  | Sapling | **1** | 0 | 0 | 0 | 0 | 1.1* |
| *Rhus glabra* | Total | 32 | 38 | **110** | 2 | 39 | 1.0-3.9 |
|  | Adult | 0 | 0 | **0** | 0 | 0 | NA |
|  | Sapling | 32 | 38 | **110** | 2 | 39 | 1.0-3.9 |
| *Salix* | Total | 0 | 0 | 0 | **46** | 0 | 1.0-2.6 |
| *amygdaloides* | Adult | 0 | 0 | 0 | **0** | 0 | NA |
|  | Sapling | 0 | 0 | 0 | **46** | 0 | 1.0-2.6 |
| *Salix eriocephala* | Total | 0 | 0 | 0 | **12** | 0 | 1.0-3.1 |
|  | Adult | 0 | 0 | 0 | **0** | 0 | NA |
|  | Sapling | 0 | 0 | 0 | **12** | 0 | 1.0-3.1 |
| *Salix interior* | Total | 0 | 0 | 0 | **58** | 0 | 1.0-1.7 |
|  | Adult | 0 | 0 | 0 | **0** | 0 | NA |
|  | Sapling | 0 | 0 | 0 | **58** | 0 | 1.0-1.7 |
| *Tilia americana* | Total | 121 | **284** | 41 | 16 | 1 | 1.0-92.7 |
|  | Adult | 91 | **264** | 35 | 14 | 1 | 5.2-92.7 |
|  | Sapling | 30 | **20** | 6 | 2 | 0 | 1.0-4.9 |
| *Ulmus americana* | Total | 6 | **11** | 3 | 5 | 0 | 1.2-47.3 |
|  | Adult | 6 | **7** | 3 | 4 | 0 | 7.1-47.3 |
|  | Sapling | 0 | **4** | 0 | 1 | 0 | 1.2-4.1 |
| *Ulmus laevis* | Total | 0 | 0 | **1** | 0 | 0 | 1.2* |
|  | Adult | 0 | 0 | **0** | 0 | 0 | NA |
|  | Sapling | 0 | 0 | **1** | 0 | 0 | 1.2* |
| *Ulmus rubra* | Total | 52 | **62** | 19 | 7 | 0 | 1-61.5 |
|  | Adult | 30 | **57** | 14 | 7 | 0 | 5.1-61.5 |
|  | Sapling | 22 | **5** | 5 | 0 | 0 | 1.0-4.6 |
| *Vitis riparia* | Total | **88** | **88** | 34 | 26 | 0 | 1.0-10.9 |
|  | Adult | **6** | **6** | 3 | 5 | 0 | 5.2-10.9 |
|  | Sapling | **82** | **82** | 31 | 21 | 0 | 1.0-5.0 |

*Indicates only one individual of that species with a diameter $\geq$1 cm (i.e., no DBH range).

Table S3. Loadings of five topographic variables onto the first five principal components (PCs) of a principal components analysis used to define five categorical habitats across the Niobrara plot. Aspect is decomposed into northness and eastness. Topographic variables were derived from a digital elevation model. Cutoffs of PC1 and PC2 were used to define the habitats, and values less and greater than |0.4| are shown in bold. See *Methods* and Fig. S3 for details.

| **Topographic variable** | **PC1** | **PC2** | **PC3** | **PC4** | **PC5** |
| --- | --- | --- | --- | --- | --- |
| **Solar radiation (Wh/m^2^)** | **-0.61732** | 0.24194 | -0.04688 | 0.06493 | **-0.74430** |
| **Elevation (m)** | -0.37240 | -0.12680 | **0.77426** | **-0.46247** | 0.17855 |
| **Slope (%)** | **0.56816** | -0.29222 | 0.09380 | **-0.45663** | **-0.61196** |
| **Eastness** | -0.14922 | **-0.82778** | 0.12518 | **0.51490** | -0.10828 |
| **Northness** | 0.36765 | 0.39342 | **0.61144** | **0.55523** | -0.16712 |

Table S4. Summary of habitat-related variation in microclimate in the Niobrara plot. Microclimate data for each habitat were collected at ten monitoring stations from April to November in 2021 and 2022: Canyon Bottoms, Stations 1 and 2; Upland Forest, Stations 3 and 4; Upper Canyon, Stations 5-8; Floodplain, Station 9; and Grassland, Station 10 (Fig. S2). Values are the within-habitat average of the within-station average of the daily means across all measurement days, except for photosynthetic photon flux density (PPFD), which is the average of the daily total PPFD across all measurement days. All values are shown ± standard deviation, and the average daily minimums and maximums for air temperature (air temp), relative humidity (RH), soil temperature (soil temp), and volumetric water content (VWC) are in parentheses. Vapor pressure deficit (VPD) was calculated from air temp and RH. In 2021, air temp and RH sensors were not deployed until 21 May for Stations 3, 7, and 9, and until 12 June for Stations 1, 4, 5, 6, and 8. In 2022, soil temperature data for Station 2 were excluded due to sensor error.

| **Year** | **Habitat** | **PPFD (mmol**  **/m^2^/d)** | **Air Temp (ºC)** | **RH (%)** | **VPD (kPa)** | **Soil Temp (ºC)** | **VWC (%)** |
| --- | --- | --- | --- | --- | --- | --- | --- |
| **2021** | **Canyon Bottoms** | 842.1 ± 325.2 | 14.8 ± 2.9 (-6.9-36.8) | 82.7 ± 8.3  (20.0-100) | 0.30 ± 0.2 | 14.4 ± 1.5  (3.0-21.7) | 30.3 ± 0.9 (26.9-34.3) |
| **2022** |  | 977.9 ± 453.9 | 14.6 ± 3.8 (-6.3-37.7) | 73.5 ± 10.0  (14.4-100) | 0.46 ± 0.2 | 13.2 ± 1.8  (2.9-21.1) | 26.7 ± 0.4 (22.8-31.8) |
| **2021** | **Upland Forest** | 370.6 ± 124.8 | 16.9 ± 3.0 (-7.1-38.7) | 71.8 ± 10.4  (0.0-100) | 0.61 ± 0.3 | 15.6 ± 1.9  (-0.03-25.3) | 22.3 ± 0.7 (19.6-26.6) |
| **2022** |  | 689.8 ± 292.3 | 16.1 ± 4.4 (-9.1-41.2) | 61.0 ± 11.9  (12.4-100) | 0.79 ± 0.4 | 14.3 ± 2.5  (-7.4-24.5) | 20.8 ± 0.8 (14.7-25.9) |
| **2021** | **Upper Canyon** | 644.9 ± 203.1 | 17.0 ± 3.3 (-7.7-42.9) | 72.0 ± 10.7  (13.7-100) | 0.62 ± 0.3 | 15.9 ± 1.7  (2.8-26.0) | 23.3 ± 0.4 (17.7-37.5) |
| **2022** |  | 906.0 ± 338.5 | 16.2 ± 4.5 (-9.4-45.6) | 63.5 ± 12.7  (9.9-100) | 0.76 ± 0.4 | 14.6 ± 2.3  (1.1-27.0) | 22.7 ± 0.4 (17.0-35.5) |
| **2021** | **Flood-plain** | 4337.7 ± 1181.8 | 16.4 ± 2.9 (-8.4-39.3) | 83.1 ± 6.2  (17.7-100) | 0.36 ± 0.2 | 15.8 ± 1.7  (3.2-23.6) | 26.7 ± 0.6 (20.7-34.8) |
| **2022** |  | 4985.4 ± 1296.2 | 15.3 ± 4.2 (-10.2-42.2) | 68.2 ± 12.7  (12.1-100) | 0.58 ± 0.3 | 12.5 ± 2.3  (0.1-21.1) | 25.8 ± 1.1 (14.7-33.9) |
| **2021** | **Grass-land** | 5270.2 ± 1474.3 | 16.4 ± 3.9 (-7.4-41.0) | 66.4 ± 11.6  (15.9-100) | 0.74 ± 0.4 | 20.9 ± 2.9  (2.1-37.1) | 20.1 ± 0.6 (18.2-22.2) |
| **2022** |  | 5661.5 ± 1631.4 | 16.5 ± 4.8 (-9.4-42.3) | 58.7 ± 14.6  (8.4-100) | 0.89 ± 0.5 | 22.1 ± 4.1  (1.9-37.0) | 19.7 ± 0.7 (17.7-23.6) |

Table S5. Summary statistics for permutational analyses of variance testing the effects of habitat type and continuous topographic variation on microclimate means and coefficients of variation (CVs) across the 2021 and 2022 growing seasons at the monitoring stations in the Niobrara plot. Month was nested within habitat to account for multiple months of sampling at each station and was included as an additive effect in continuous models. Interactions between variables are indicated by “*”; DF = degrees of freedom; significant effects at the $\boldsymbol{\alpha}$ = 0.05 level shown in bold.

|  | **Habitat Type** | | | | | |
| --- | --- | --- | --- | --- | --- | --- |
| **Year** | **Response Variable** | **Model Term** | ***F*-value** | **DF (Error, Total)** | ***R*^2^** | ***p*-value** |
| 2021 | Microclimate | Habitat | 52.656 | 4,79 | 0.39 | **0.001** |
|  | mean | Habitat*Month | 8.366 | 35,79 | 0.54 | **0.001** |
| 2022 | Microclimate | Habitat | 39.527 | 4,79 | 0.32 | **0.001** |
|  | mean | Habitat*Month | 8.222 | 35,79 | 0.59 | **0.001** |
| 2021 | Microclimate | Habitat | 11.161 | 4,79 | 0.11 | **0.001** |
|  | CV | Habitat*Month | 0.796 | 35,79 | 0.80 | **0.001** |
| 2022 | Microclimate | Habitat | 13.148 | 4,79 | 0.10 | **0.001** |
|  | CV | Habitat*Month | 12.936 | 35,79 | 0.83 | **0.001** |
|  | **Continuous Topographic Variation** | | | | | |
| **Year** | **Response Variable** | **Model Term** | ***F*-value** | **DF (Error, Total)** | ***R*^2^** | ***p*-value** |
| 2021 | Microclimate | Topographic PC1 | 34.790 | 1,79 | 0.09 | **0.001** |
|  | mean | Topographic PC2 | 16.837 | 1,79 | 0.04 | **0.001** |
|  |  | Topographic PC3 | 54.953 | 1,79 | 0.14 | **0.001** |
|  |  | Month | 29.737 | 7,79 | 0.55 | **0.001** |
| 2022 | Microclimate | Topographic PC1 | 29.489 | 1,79 | 0.08 | **0.001** |
|  | mean | Topographic PC2 | 9.816 | 1,79 | 0.03 | **0.001** |
|  |  | Topographic PC3 | 43.776 | 1,79 | 0.12 | **0.001** |
|  |  | Month | 29.684 | 7,79 | 0.58 | **0.001** |
| 2021 | Microclimate | Topographic PC1 | 3.826 | 1,79 | 0.01 | **0.028** |
|  | CV | Topographic PC2 | 6.554 | 1,79 | 0.02 | **0.002** |
|  |  | Topographic PC3 | 13.909 | 1,79 | 0.04 | **0.001** |
|  |  | Month | 34.646 | 7,79 | 0.72 | **0.001** |
| 2022 | Microclimate | Topographic PC1 | 9.520 | 1,79 | 0.03 | **0.001** |
|  | CV | Topographic PC2 | 1.843 | 1,79 | 0.01 | 0.125 |
|  |  | Topographic PC3 | 13.684 | 1,79 | 0.04 | **0.001** |
|  |  | Month | 31.266 | 7,79 | 0.70 | **0.001** |

Table S6. Means and standard deviations (SDs) of differences between nine forested microclimate monitoring stations versus one station in grassland representing above- and belowground understory microclimate buffering in the Niobrara plot in 2021 and 2022. Microclimate buffering was estimated based on the differences in the mean daily air temperature (air temp), relative humidity (RH), vapor pressure deficit (VPD), soil temperature (soil temp), and soil moisture content (volumetric water content, VWC) of each of the nine microclimate stations located in four forest understory habitats (Stations 1-9) minus Station 10 in grassland. These differences were averaged within months (April-November), and the overall mean station differences (± SD of difference) shown below were calculated on monthly station values. Aboveground (AG) microclimate buffering was assessed using differences in air temp, RH, and VPD, whereas belowground (BG) microclimate buffering was assessed using differences in soil temp and VWC. Significant buffering of forested habitats was evaluated using a paired t-test between mean monthly microclimate conditions at each forested station versus the grassland station, with significant differences at $\boldsymbol{\alpha}$ = 0.05 level shown in bold. See Fig. S2 for map of the microclimate monitoring stations.

|  |  |  | **AG microclimate buffering** | | | | **BG microclimate buffering** | |
| --- | --- | --- | --- | --- | --- | --- | --- | --- |
| **Habitat** | **Station Number** | **Year** | **Air Temp (ºC)** | **RH (%)** | **VPD (kPa)** | **Soil Temp (ºC)** | | **VWC (%)** |
| **Canyon** | 1 | 2021 | **-2.6 ± 0.9** | **15.2 ± 4.9** | **-0.5 ± 0.3** | **-7.3 ± 3.2** | | **9.3 ± 1.7** |
| **Bottoms** | 1 | 2022 | **-2.3 ± 1.0** | **12.9 ± 4.4** | **-0.4 ± 0.2** | **-9.0 ± 3.3** | | **5.4 ± 1.3** |
| **Canyon** | 2 | 2021 | **-2.3 ± 1.3** | **17.3 ± 5.3** | **-0.5 ± 0.3** | **-7.4 ± 4.0** | | **11.5 ± 0.8** |
| **Bottoms** | 2 | 2022 | **-2.0 ± 1.1** | **16.6 ± 4.9** | **-0.5 ± 0.2** | **-8.8 ± 3.6** | | **8.5 ± 1.2** |
| **Upland** | 3 | 2021 | -0.8 ± 0.4 | **5.2 ± 1.1** | **-0.2 ± 0.1** | **-6.6 ± 2.6** | | **2.4 ± 0.5** |
| **Forest** | 3 | 2022 | **-0.5 ± 0.5** | **3.9 ± 2.0** | **-0.1 ± 0.1** | **-8.0 ± 3.1** | | **1.1 ± 0.9** |
| **Upland** | 4 | 2021 | **-1.2 ± 0.4** | **4.5 ± 1.9** | **-0.2 ± 0.1** | **-6.5 ± 2.0** | | **2.6 ± 0.6** |
| **Forest** | 4 | 2022 | **-0.6 ± 0.4** | **1.3 ± 1.2** | **-0.1 ± 0.1** | **-7.7 ± 2.7** | | **1.1 ± 0.8** |
| **Upper** | 5 | 2021 | **-1.2 ± 0.4** | **4.6 ± 1.0** | **-0.2 ± 0.1** | **-7.3 ± 2.9** | | **13.4 ± 1.0** |
| **Canyon** | 5 | 2022 | **-0.7 ± 0.4** | **4.1 ± 1.5** | **-0.1 ± 0.1** | **-10.2 ± 3.1** | | **12.3 ± 0.9** |
| **Upper** | 6 | 2021 | **-1.5 ± 0.5** | **7.7 ± 2.3** | **-0.3 ± 0.1** | **-6.6 ± 2.8** | | **0.9 ± 1.0** |
| **Canyon** | 6 | 2022 | **-1.0 ± 0.6** | **6.1 ± 2.6** | **-0.2 ± 0.1** | **-7.4 ± 3.4** | | **1.3 ± 0.7** |
| **Upper** | 7 | 2021 | -0.9 ± 0.3 | **3.8 ± 1.6** | **-0.1 ± 0.1** | **-6.5 ± 2.5** | | **-1.6 ± 0.7** |
| **Canyon** | 7 | 2022 | -0.2 ± 0.4 | **3.4 ± 2.5** | **-0.1 ± 0.1** | **-7.4 ± 2.7** | | **-1.9 ± 0.7** |
| **Upper** | 8 | 2021 | **-0.9 ± 0.2** | **5.7 ± 0.9** | **-0.2 ± 0.1** | **-4.7 ± 2.2** | | 0.1 ± 0.6 |
| **Canyon** | 8 | 2022 | 0.15 ± 0.3 | **4.8 ± 1.3** | **-0.1 ± 0.1** | **-5.4 ± 2.9** | | 0.3 ± 0.6 |
| **Flood-** | 9 | 2021 | -1.3 ± 1.0 | **15.7 ± 7.5** | **-0.4 ± 0.3** | **-6.0 ± 2.6** | | **7.0 ± 2.0** |
| **plain** | 9 | 2022 | **-1.2 ± 0.9** | **9.5 ± 5.1** | **-0.3 ± 0.2** | **-9.6 ± 2.7** | | **6.0 ± 3.8** |

Table S7. Summary statistics for permutational analyses of variance testing the effects of the presence of the overstory (basal area) and topography (elevation and northness) on average above- and belowground understory microclimate buffering in 2021 and 2022 in the Niobrara plot. Microclimate buffering was estimated based on the differences of each of the nine microclimate stations located in the forest understory (Stations 1-9) minus Station 10 in grassland. Differences were calculated using the daily means of aboveground (AG: air temperature, relative humidity, vapor pressure deficit) and belowground (BG: soil temperature and soil moisture (volumetric water content) variables, and were averaged within months (April-November). Total basal area was calculated in a 5-m radius of each station, station elevation and northness were determined from a digital elevation model, and separate models were fit using elevation and northness due to the modest number of stations. DF = degrees of freedom; significant effects at $\boldsymbol{\alpha}$ = 0.05 level shown in bold.

| **Year** | **Response Variable** | **Model Term** | ***F*-value** | **DF (Error, Total)** | ***R*^2^** | ***p*-value** |
| --- | --- | --- | --- | --- | --- | --- |
| 2021 | AG microclimate | Basal area | 7.9 | 1,71 | 0.09 | **0.005** |
|  | buffering | Elevation | 9.6 | 1,71 | 0.11 | **0.002** |
|  | BG microclimate | Basal area | 5.8 | 1,71 | 0.07 | **0.009** |
|  | buffering | Elevation | 3.4 | 1,71 | 0.04 | **0.050** |
| 2021 | AG microclimate | Basal area | 3.4 | 1,71 | 0.18 | **0.001** |
|  | buffering | Northness | 15.5 | 1,71 | 0.04 | **0.052** |
|  | BG microclimate | Basal area | 3.6 | 1,71 | 0.05 | **0.039** |
|  | buffering | Northness | 4.6 | 1,71 | 0.06 | **0.018** |
| 2022 | AG microclimate | Basal area | 11.1 | 1,71 | 0.12 | **0.003** |
|  | buffering | Elevation | 13.2 | 1,71 | 0.14 | **0.001** |
|  | BG microclimate | Basal area | 4.6 | 1,71 | 0.06 | **0.024** |
|  | buffering | Elevation | 1.1 | 1,71 | 0.01 | 0.314 |
| 2022 | AG microclimate | Basal area | 5.3 | 1,71 | 0.06 | **0.028** |
|  | buffering | Northness | 17.8 | 1,71 | 0.19 | **0.001** |
|  | BG microclimate | Basal area | 2.2 | 1,71 | 0.03 | 0.131 |
|  | buffering | Northness | 7.6 | 1,71 | 0.10 | **0.005** |

Table S8. Topographic niches of 22 woody species along a continuous elevation gradient in the Niobrara plot. Probabilities (*p*-values) for the observed elevation means and standard deviations (SD) (m) are based on species’ ranks relative to 999 random distributions generated using conditional torus translations of trees within quadrats (see *Methods* section) and were assessed at the $\boldsymbol{\alpha}$ = 0.05 level, with significant values (in bold) indicating lower-elevation (one-tailed test for mean elevation) and narrower (two-tailed test for the elevation SD) niches than expected by chance. Abundance is the total number of individuals of each species in the Niobrara plot, for species with at least three individuals. CI = confidence interval.

| **Species** | **Abundance** | **Observed Mean (*p*-value)** | **Randomized Mean (CI)** | **Observed SD (*p*-value)** | **Randomized SD (CI)** |
| --- | --- | --- | --- | --- | --- |
| *Acer*  *negundo* | 4 | 662.5 (0.098) | 680.2 (658.2, 692.9) | 8.7 (0.112) | 15.8 (5.4, 20.7) |
| *Betula papyrifera* | 28 | 664.6 (0.061) | 679.5 (662.2, 694.7) | 5.6 (**0.002**) | 16.4 (10.1, 21.6) |
| *Celastrus scandens* | 8 | 669.5 (0.084) | 683.2 (661.8, 695) | 15.3 (0.414) | 15.8 (9, 20.5) |
| *Celtis occidentalis* | 277 | 662.2 (**0.003**) | 680.9 (669.8, 690.6) | 10.8 (**0.004**) | 18.2 (14.8, 21) |
| *Fraxinus pennsylvanica* | 410 | 657.2 (**0.002**) | 680.7 (658.7, 691.1) | 13.2 (**0.007**) | 16.6 (13.6, 20.7) |
| *Juglans*  *nigra* | 27 | 662.7 (**0.049**) | 681.5 (661.1, 693.5) | 8.8 (**0.001**) | 17 (11.3, 21.1) |
| *Juniperus virginiana* | 2830 | 674.6 (0.268) | 678.2 (668.1, 688) | 10.7 (**0.001**) | 16.9 (13, 19.9) |
| *Ostrya virginiana* | 1569 | 663.1 (**0.015**) | 676.6 (664, 687) | 7.5 (**0.002**) | 17.5 (13.5, 21.1) |
| *Parthenocissus quinquefolia* | 24 | 666.1 (**0.002**) | 685.2 (673.5, 693.1) | 17 (0.453) | 17.4 (14.3, 21) |
| *Pinus ponderosa* | 219 | 680 (0.488) | 679.2 (663.1, 691.5) | 7.3 (**0.002**) | 16 (11, 21.4) |
| *Populus deltoides* | 188 | 645.5 (**0.010**) | 674.2 (646.2, 695.5) | 3.9 (0.109) | 7.3 (3.1, 20.6) |
| *Prunus americana* | 47 | 688.4 (0.699) | 682.1 (661.9, 695.5) | 16.7 (0.671) | 14 (5.9, 20.2) |
| *Prunus virginiana* | 521 | 672.7 (0.251) | 677.8 (664, 690.2) | 9.7 (**0.003**) | 16.8 (12.1, 20.4) |
| *Quercus macrocarpa* | 939 | 675.3 (0.091) | 678.7 (673.5, 683.1) | 14.1 (**0.002**) | 18.2 (16.4, 19.8) |
| *Rhus*  *glabra* | 221 | 681.2 (0.594) | 678.7 (659.3, 691.6) | 13.5 (0.269) | 15.8 (9.8, 21.2) |
| *Salix amygdaloides* | 46 | 644.8 (**0.002**) | 675.3 (645.9, 695) | 0.3 (**0.002**) | 6.1 (0.8, 19.2) |
| *Salix eriocephala* | 12 | 644.8 (**0.009**) | 674.5 (645.4, 694.9) | 0.4 (**0.022**) | 5.8 (0.4, 19.6) |
| *Salix*  *interior* | 58 | 644.8 (**0.001**) | 675.7 (645.7, 696) | 0.3 (**0.001**) | 6.5 (0.8, 19.6) |
| *Tilia americana* | 463 | 663.5 (**0.046**) | 678 (662.8, 687.2) | 8.6 (**0.003**) | 17.9 (13.1, 21.2) |
| *Ulmus americana* | 25 | 658.4 (**0.002**) | 682 (661.5, 694) | 8.9 (**0.011**) | 17 (11.2, 21) |
| *Ulmus*  *rubra* | 140 | 663.7 (**0.008**) | 680.6 (667, 692) | 8.0 (**0.002**) | 17.6 (13, 21.2) |
| *Vitis*  *riparia* | 236 | 664 (**0.001**) | 681.8 (671.2, 690.6) | 10.8 (**0.002**) | 17.9 (15.1, 20.2) |

Table S9. Summary statistics for linear models testing variation in forest structure (tree density, basal area, and aboveground biomass) and diversity (species richness and Shannon’s diversity index) among habitat types in the Niobrara plot. DF = degrees of freedom; significant effects at the $\boldsymbol{\alpha}$ = 0.05 level shown in bold.

| **Response Variable** | ***F*-value** | **DF (Error, Total)** | ***R*^2^** | ***p*-value** |
| --- | --- | --- | --- | --- |
| Tree density | 37.3 | 4, 334 | 0.30 | **<0.001** |
| Basal area | 31.9 | 4, 334 | 0.27 | **<0.001** |
| Aboveground biomass | 28.9 | 4, 334 | 0.25 | **<0.001** |
| Species richness | 58.7 | 4, 334 | 0.41 | **<0.001** |
| Shannon’s diversity | 55.4 | 4, 334 | 0.39 | **<0.001** |

Table S10. Results from AIC model selection for the top two models, global model, and null model predicting forest structure (tree density, basal area, and aboveground biomass) and diversity (species richness) as a function of variation in topographic variables across the Niobrara plot. k: number of model parameters; weight = calculated Akaike weight; *R*^2^ = adjusted *R*^2^; ^2 indicates all two-way interactions between the variables inside the parentheses were included in the model; solar: cumulative annual solar radiation (Wh/m^2^); s(x,y): the two-dimensional thin-plate spline between x and y. Transformations applied to response variables to address non-normality are indicated in parentheses around each variable.

| **Response Variable** | **Model** | **AIC** | **k** | **∆AIC** | **Weight** | ***R*^2^** |
| --- | --- | --- | --- | --- | --- | --- |
| log  (tree | **Top**: ~ (elevation + eastness + slope)^2 + s(x,y) | 714.9 | 37 | 0 | 0.756 | 0.69 |
| density) | **2^nd^**: ~ (elevation + northness + eastness + slope)^2 + s(x,y) | 717.4 | 41 | 2.5 | 0.218 | 0.69 |
|  | **Global**: ~ (elevation + northness + eastness + solar + slope)^2 + s(x,y) | 751.2 | 24 | 36.3 | 0 | 0.54 |
|  | **Null**: ~ 1 | 1086 | 2 | 371.2 | 0 | 0 |
| square root (basal area) | **Top**: ~ (elevation + northness + slope)^2 + s(x,y) | 59.64 | 37 | 0 | 0.483 | 0.56 |
|  | **2^nd^**: ~ (elevation + northness + eastness + slope)^2 + s(x,y) | 61.74 | 41 | 2.1 | 0.169 | 0.56 |
|  | **Global**: ~ (elevation + northness + eastness + solar + slope)^2 + s(x,y) | 73.86 | 27 | 14.2 | 0 | 0.53 |
|  | **Null**: ~ 1 | 307.3 | 2 | 247.6 | 0 | 0 |
| square root (above - | **Top**: ~ (elevation + northness + slope)^2 + s(x,y) | 3103 | 37 | 0 | 0.227 | 0.52 |
| ground | **2^nd^**: ~ northness * slope + s(x,y) | 3103 | 34 | 0.05 | 0.221 | 0.52 |
| biomass) | **Global**: ~ (elevation + northness + eastness + solar + slope)^2 + s(x,y) | 3114 | 27 | 11.3 | 0.001 | 0.49 |
|  | **Null**: ~ 1 | 3324 | 2 | 221.2 | 0 | 0 |
| cubed root (species | **Top**: ~ (elevation + northness + slope)^2 + s(x,y) | -211.8 | 37 | 0 | 0.442 | 0.76 |
| richness) | **2^nd^**: ~ elevation + slope + s(x,y) | -209.2 | 33 | 2.6 | 0.121 | 0.75 |
|  | **Global**: ~ (elevation + northness + eastness + solar + slope)^2 + s(x,y) | -178.9 | 27 | 32.8 | 0 | 0.72 |
|  | **Null**: ~ 1 | 238.8 | 2 | 450.5 | 0 | 0 |

Table S11. Summary statistics for the top candidate models of forest structure and diversity as a function of topography across the Niobrara plot. Parameter estimates, standard errors, *t*-values, and *p*-values for the top candidate models predicting tree density (adjusted *R*^2^ = 0.69), basal area (adjusted *R*^2^ = 0.56), aboveground biomass (adjusted *R*^2^ = 0.52), and species richness (adjusted *R*^2^ = 0.76) as a function of variation in topographic variables. Interactions between variables are indicated by “*”; significant effects at the $\boldsymbol{\alpha}$ = 0.05 level shown in bold. Parameters have not been back transformed.

| **Response Variable** | **Model Term** | **Estimate** | **Standard Error** | ***t*-value** | ***p*-value** |
| --- | --- | --- | --- | --- | --- |
| Tree density | Intercept | 7.215 | 0.490 | 14.717 | **<0.001** |
|  | elevation | -0.108 | 0.015 | -7.091 | **<0.001** |
|  | eastness | 0.234 | 0.182 | 1.284 | 0.200 |
|  | slope | -0.0005 | 0.015 | -0.035 | 0.972 |
|  | elevation*eastness | 0.007 | 0.004 | 1.732 | 0.084 |
|  | elevation*slope | 0.003 | 0.0004 | 6.309 | **<0.001** |
|  | eastness*slope | -0.019 | 0.007 | -2.909 | **0.003** |
| Basal area | Intercept | 3.983 | 0.982 | 4.054 | **<0.001** |
|  | elevation | -0.080 | 0.031 | -2.596 | **0.010** |
|  | northness | -0.455 | 0.437 | -1.041 | 0.299 |
|  | slope | 0.052 | 0.029 | 1.798 | 0.073 |
|  | elevation*northness | -0.003 | 0.010 | -0.300 | 0.765 |
|  | elevation*slope | 0.002 | 0.0008 | 2.375 | **0.018** |
|  | northness*slope | 0.042 | 0.016 | 2.695 | **0.007** |
| Aboveground | Intercept | 10.190 | 2.752 | 3.703 | **<0.001** |
| biomass | elevation | -0.196 | 0.087 | -2.263 | **0.024** |
|  | northness | -1.096 | 1.228 | -0.892 | 0.373 |
|  | slope | 0.136 | 0.082 | 1.663 | 0.097 |
|  | elevation*northness | -0.006 | 0.027 | -0.232 | 0.816 |
|  | elevation*slope | 0.005 | 0.002 | 1.959 | **0.051** |
|  | northness*slope | 0.098 | 0.044 | 2.233 | **0.027** |
| Species | Intercept | 1.630 | 0.126 | 12.984 | **<0.001** |
| richness | elevation | -0.019 | 0.004 | -4.872 | **<0.001** |
|  | northness | -0.117 | 0.058 | -2.012 | **0.045** |
|  | slope | 0.021 | 0.004 | 5.506 | **<0.001** |
|  | elevation*northness | 0.004 | 0.001 | 2.966 | **0.003** |
|  | elevation*slope | 0.00003 | 0.0001 | 0.247 | 0.805 |
|  | northness*slope | -0.0006 | 0.002 | -0.278 | 0.781 |

Table S12. Summary statistics for the linear models testing the influence of 2021 and 2022 microclimate means and coefficients of variation (CVs) on forest structure (tree density, basal area, and aboveground biomass) and diversity (species richness) in the Niobrara plot. Metrics of forest structure and diversity were calculated in a 10-m radius around each microclimate station. Due to limited station-level replication (9 stations), analyses only included the following microclimate variables: vapor pressure deficit (VPD), soil temperature, and surface soil moisture (volumetric water content, VWC). SE = standard error; significant effects at the $\boldsymbol{\alpha}$ = 0.05 level are shown in bold.

|  | **Microclimate Means** | | | | | |
| --- | --- | --- | --- | --- | --- | --- |
| **Year** | **Response Variable (overall *R*^2^)** | **Model Term** | **Estimate** | **SE** | ***t*-value** | ***p*-value** |
| 2021 | Tree density | Intercept | 39.761 | 257.791 | 0.154 | 0.883 |
|  | (*R*^2^ = -0.29) | VWC | -1.845 | 13.767 | -0.134 | 0.899 |
|  |  | Soil temperature | 3.412 | 11.966 | 0.285 | 0.787 |
|  |  | VPD | -11.744 | 11.537 | -1.018 | 0.355 |
| 2022 | Tree density | Intercept | 173.223 | 209.025 | 0.829 | 0.445 |
|  | (*R*^2^ = -0.21) | VWC | -6.784 | 14.651 | -0.463 | 0.663 |
|  |  | Soil temperature | -2.334 | 13.581 | -0.172 | 0.870 |
|  |  | VPD | -13.315 | 10.637 | -1.252 | 0.266 |
| 2021 | Basal area | Intercept | 6.774 | 3.132 | 2.162 | 0.083 |
|  | (*R*^2^ = 0.75) | VWC | -0.272 | 0.167 | -1.629 | 0.164 |
|  |  | Soil temperature | -0.347 | 0.145 | -2.390 | 0.062 |
|  |  | VPD | 0.489 | 0.140 | 3.490 | **0.017** |
| 2022 | Basal area | Intercept | -0.112 | 4.068 | -0.028 | 0.979 |
|  | (*R*^2^ = 0.40) | VWC | -0.104 | 0.285 | -0.365 | 0.730 |
|  |  | Soil temperature | -0.055 | 0.264 | -0.208 | 0.843 |
|  |  | VPD | 0.477 | 0.207 | 2.304 | 0.069 |
| 2021 | AGB | Intercept | 38.174 | 25.507 | 1.497 | 0.195 |
|  | (*R*^2^ = 0.68) | VWC | -1.450 | 1.362 | -1.064 | 0.336 |
|  |  | Soil temperature | -2.126 | 1.184 | -1.796 | 0.132 |
|  |  | VPD | 3.667 | 1.142 | 3.213 | **0.024** |
| 2022 | AGB | Intercept | 1.623 | 26.453 | 0.061 | 0.953 |
|  | (*R*^2^ = 0.50) | VWC | -0.806 | 1.854 | -0.435 | 0.682 |
|  |  | Soil temperature | -0.864 | 1.719 | -0.503 | 0.636 |
|  |  | VPD | 3.712 | 1.346 | 2.757 | **0.040** |
| 2021 | Species | Intercept | 32.510 | 16.971 | 1.916 | 0.114 |
|  | richness | VWC | -1.046 | 0.906 | -1.154 | 0.300 |
|  | (*R*^2^ = 0.62) | Soil temperature | -0.666 | 0.788 | -0.846 | 0.436 |
|  |  | VPD | -2.781 | 0.759 | -3.662 | **0.015** |
| 2022 | Species | Intercept | 39.305 | 12.616 | 3.115 | **0.026** |
|  | richness | VWC | -1.586 | 0.884 | -1.793 | 0.133 |
|  | (*R*^2^ = 0.70) | Soil temperature | -1.104 | 0.819 | -1.347 | 0.236 |
|  |  | VPD | -2.793 | 0.642 | -4.350 | **0.007** |
|  | **Microclimate CVs** | | | | | |
|  | **Response Variable (overall *R*^2^)** | **Model Term** | **Estimate** | **SE** | ***t*-value** | ***p*-value** |
| 2021 | Tree density | Intercept | 331.588 | 158.122 | 2.097 | 0.090 |
|  | (*R*^2^ = 0.41) | VWC | -1.599 | 8.218 | -0.195 | 0.853 |
|  |  | Soil temperature | -23.983 | 9.087 | -2.639 | **0.046** |
|  |  | VPD | -10.655 | 9.995 | -1.066 | 0.335 |
| 2022 | Tree density | Intercept | 465.060 | 181.721 | 2.559 | 0.051 |
|  | (*R*^2^ = 0.47) | VWC | -4.135 | 6.198 | -0.667 | 0.534 |
|  |  | Soil temperature | -21.529 | 7.018 | -3.067 | **0.028** |
|  |  | VPD | -10.151 | 7.108 | -1.428 | 0.213 |
| 2021 | Basal area | Intercept | -8.150 | 3.880 | -2.100 | 0.090 |
|  | (*R*^2^ = 0.53) | VWC | -0.033 | 0.202 | -0.166 | 0.874 |
|  |  | Soil temperature | 0.754 | 0.223 | 3.381 | **0.020** |
|  |  | VPD | 0.387 | 0.245 | 1.579 | 0.175 |
| 2022 | Basal area | Intercept | -2.168 | 7.448 | -0.291 | 0.783 |
|  | (*R*^2^ = -0.17) | VWC | -0.138 | 0.254 | -0.543 | 0.611 |
|  |  | Soil temperature | 0.335 | 0.288 | 1.163 | 0.297 |
|  |  | VPD | 0.011 | 0.291 | 0.039 | 0.970 |
| 2021 | AGB | Intercept | -54.422 | 22.514 | -2.417 | 0.060 |
|  | (*R*^2^ = 0.69) | VWC | 8.109 | 1.170 | 0.007 | 0.995 |
|  |  | Soil temperature | 5.429 | 1.294 | 4.196 | **0.008** |
|  |  | VPD | 2.257 | 1.423 | 1.586 | 0.174 |
| 2022 | AGB | Intercept | -26.096 | 48.285 | -0.540 | 0.612 |
|  | (*R*^2^ = 0.04) | VWC | -0.474 | 1.647 | -0.288 | 0.785 |
|  |  | Soil temperature | 3.043 | 1.865 | 1.632 | 0.164 |
|  |  | VPD | 0.191 | 1.889 | 0.101 | 0.923 |
| 2021 | Species | Intercept | 53.379 | 17.149 | 3.113 | **0.026** |
|  | richness | VWC | -1.013 | 0.891 | -1.137 | 0.307 |
|  | (*R*^2^ = 0.53) | Soil temperature | -3.006 | 0.985 | -3.050 | **0.028** |
|  |  | VPD | -2.253 | 1.084 | -2.078 | 0.092 |
| 2022 | Species | Intercept | 67.298 | 26.554 | 2.534 | **0.052** |
|  | richness | VWC | -0.507 | 0.906 | -0.559 | 0.600 |
|  | (*R*^2^ = 0.24) | Soil temperature | -2.081 | 1.026 | -2.029 | 0.098 |
|  |  | VPD | -2.052 | 1.039 | -1.975 | 0.105 |

Table S13. Summary statistics for permutational analyses of variance testing the influence of 2021 and 2022 microclimate means and coefficients of variation (CVs) on tree species composition in the Niobrara plot. Due to limited station-level replication (9 microclimate stations), analyses only included the following microclimate variables: vapor pressure deficit (VPD), soil temperature, and soil moisture (volumetric water content, VWC). DF = degrees of freedom; significant effects at the $\boldsymbol{\alpha}$ = 0.05 level shown in bold.

|  | **Microclimate Means** | | | | | |
| --- | --- | --- | --- | --- | --- | --- |
| **Year** | **Response Variable (overall *R*^2^)** | **Model Term** | ***F*-value** | **DF (Error, Total)** | ***R*^2^** | ***p*-value** |
| 2021 | Composition | VWC | 0.916 | 1,8 | 0.10 | 0.500 |
|  | (*R*^2^ = 0.53) | Soil temperature | 1.707 | 1,8 | 0.18 | 0.156 |
|  |  | VPD | 2.400 | 1,8 | 0.25 | **0.035** |
| 2022 | Composition | VWC | 1.527 | 1,8 | 0.16 | 0.183 |
|  | (*R*^2^ = 0.60) | Soil temperature | 1.785 | 1,8 | 0.19 | 0.118 |
|  |  | VPD | 2.402 | 1,8 | 0.25 | **0.026** |
|  | **Microclimate CVs** | | | | | |
| **Year** | **Response Variable (overall *R*^2^)** | **Model Term** | ***F*-value** | **DF (Error, Total)** | ***R*^2^** | ***p*-value** |
| 2021 | Composition | VWC | 0.349 | 1,8 | 0.04 | 0.925 |
|  | (*R*^2^ = 0.59) | Soil temperature | 2.869 | 1,8 | 0.30 | **0.012** |
|  |  | VPD | 2.430 | 1,8 | 0.25 | **0.052** |
| 2022 | Composition | VWC | 0.843 | 1,8 | 0.10 | 0.600 |
|  | (*R*^2^ = 0.36) | Soil temperature | 1.024 | 1,8 | 0.12 | 0.452 |
|  |  | VPD | 1.198 | 1,8 | 0.14 | 0.336 |


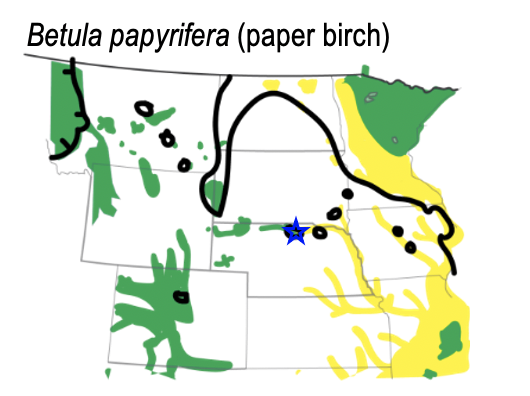

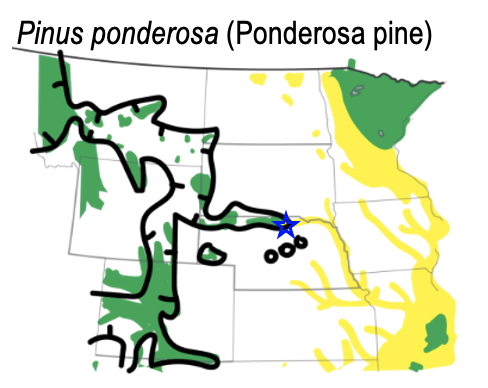

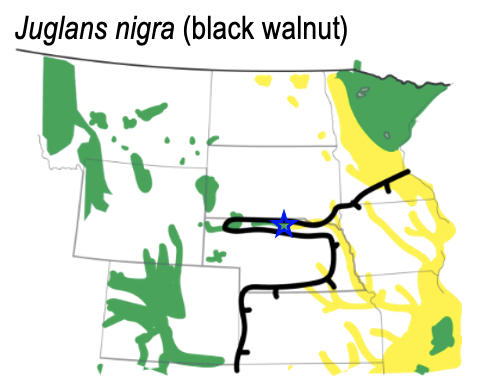


**a**

**b**

**c**

**Figure S1.** **Distributions of three woody species co-occurring along topographic gradients in a refugial forest in the North American Great Plains.** Three examples are shown of some of the many woody species with (a) northern, (b) western, and (c) eastern biogeographic origins in North America that are near their range limits in the Niobrara plot (blue star). Green and yellow areas represent the approximate extents of evergreen conifer and deciduous broad-leaved forests, respectively. Recreated from: Kaul et al. 1988.

Canyon Bottoms

Upland Forest

Upper Canyon

Lower (1) – 656.2 m, 35.6%, 228°

Upper (2) – 672.4 m, 34.2%, 206°

Lower (3) – 659.5 m, 26.4%, 319°

Upper (4) – 679.4 m, 28.9%, 324°

Lower Northeast (5) – 678.3 m, 27.3%, 266°

Lower Southwest (6) – 660.2 m, 38.6%, 194°

Upper Northeast (7) – 680.6 m, 36.3%, 312°

Upper Southwest (8) – 686.2 m, 30.4%, 174°

Floodplain (9)

– 645.1 m, 7.2%, 234°

Grassland (10)

– 696.8 m, 14.9%, 249°


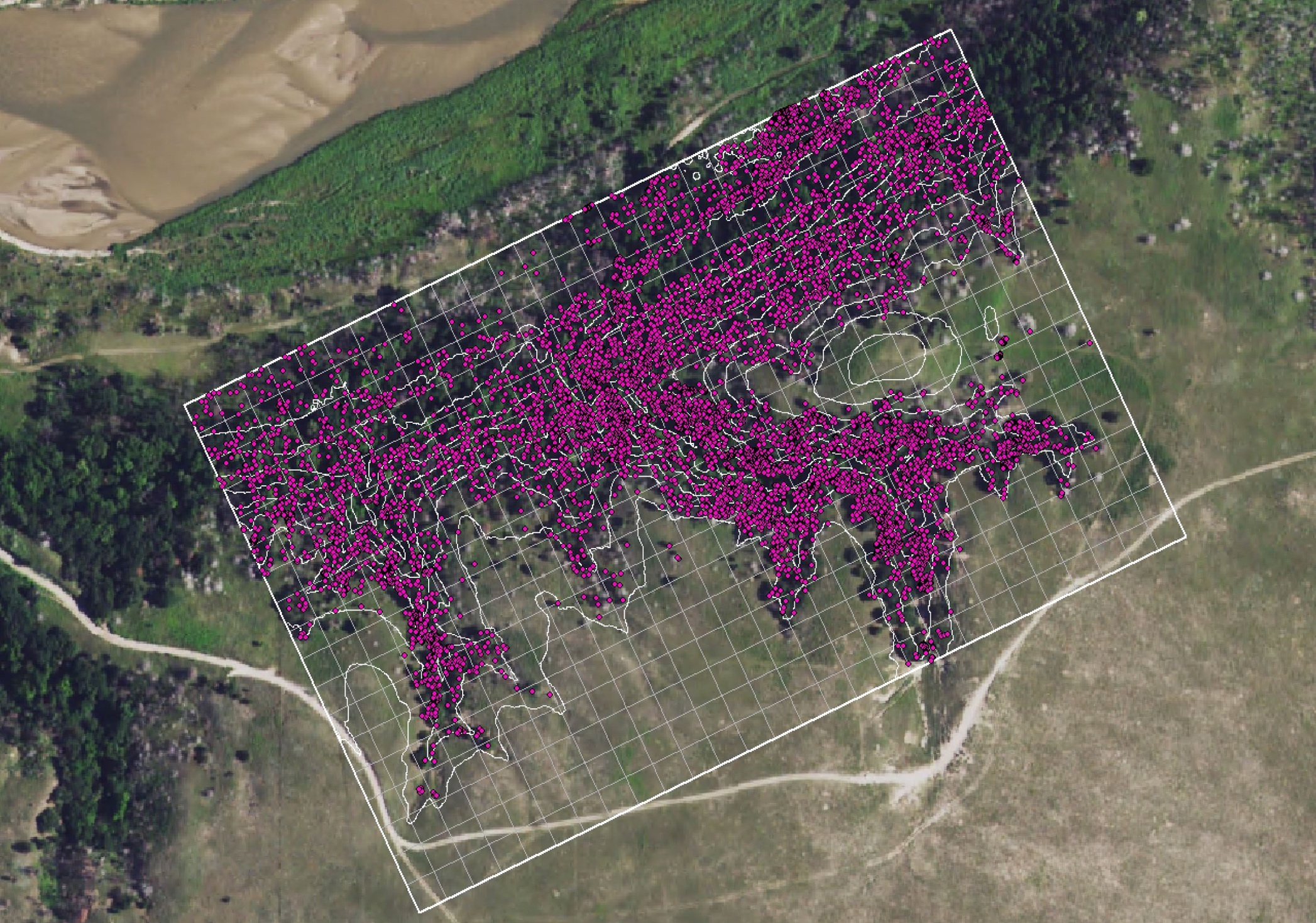


Canyon Bottoms

Upland Forest

Upper Canyon

Floodplain

Grassland


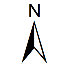


0

400

100

200

300

**1**

**2**

**3**

**4**

**6**

**5**

**8**

**7**

**9**

**10**

Meters

Figure S2. Locations of ten microclimate monitoring stations in the Niobrara plot. A contour plot (5-m elevation change between contours) overlays the Niobrara plot (560 m x 360 m, rectangular boundary indicated by thicker white line), gridlines (finer white lines) indicate the 20 m x 20 m quadrats, and each pink dot represents a mapped woody individual (DBH $\boldsymbol{\geq}$ 1 cm), all overlain on an aerial image. Numbered stars indicate the locations of microclimate stations and are color-coded by the habitat (Fig. 5a) in which they are located, except for Station 10, in the unforested grassland. The mean quadrat elevation (m), topographic slope (%), and aspect (°) for each monitoring station is indicated below the map. Sampling efforts were more intensive along the upper canyon slopes to capture the variation in conditions on the Northeast-facing (Stations 5 and 7) versus Southwest-facing (Stations 6 and 8) slopes, and from the high to low end of the main canyon (see map of cumulative annual solar radiation and thermal image of plot; Fig. 1d,e). Stations 1 and 2 were near the stream at the canyon bottom, and Station 5 was near a spring. The aerial image is a high-resolution RGBN image obtained on 18 September 2019 with an UltraCam Eagle camera (Vexcel Imaging).


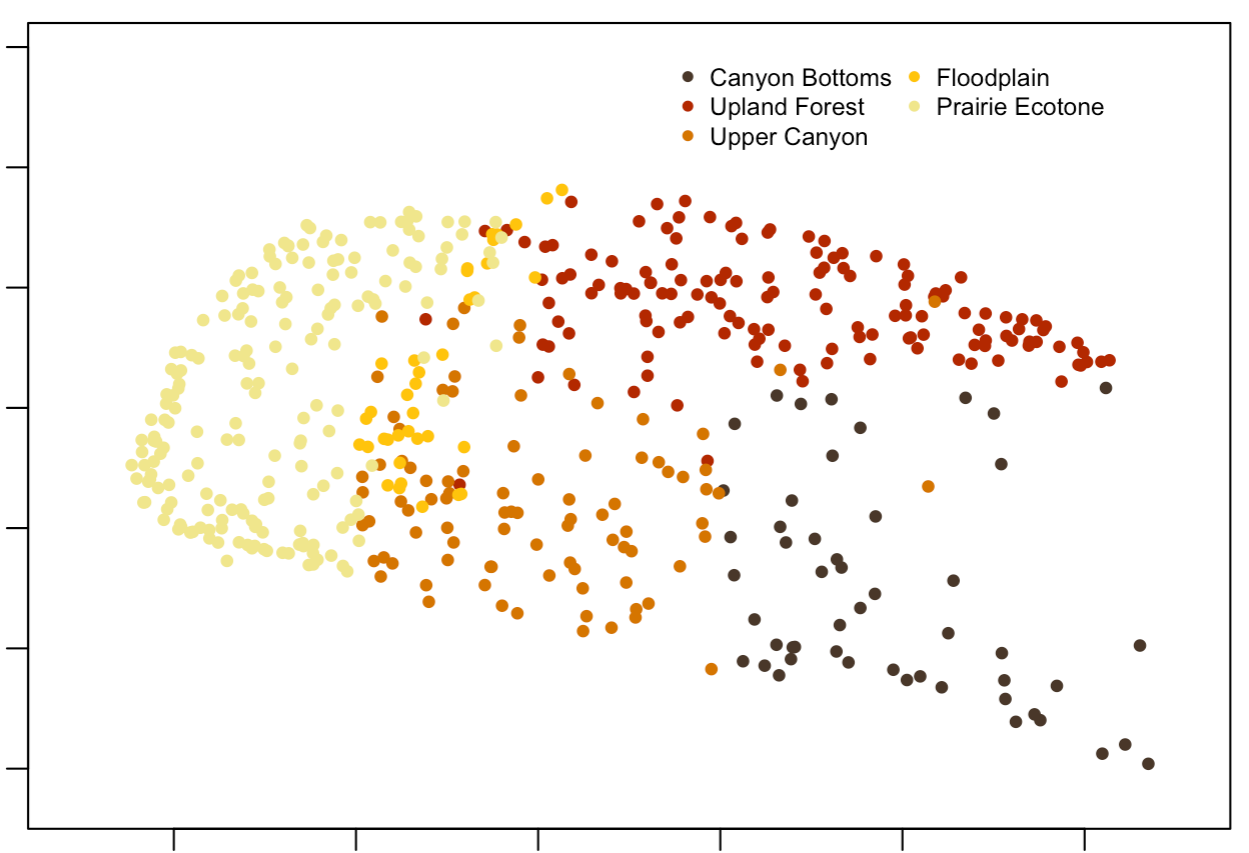


solar radiation

elevation

eastness

slope

northness


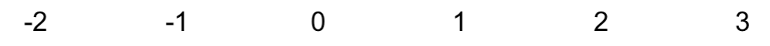


PC 1 (42.7% of variation)


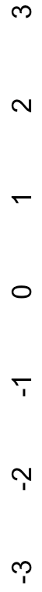


PC 2 (22.6% of variation)

**a**


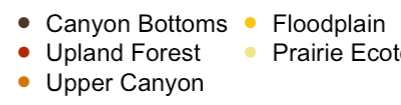

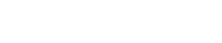

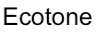

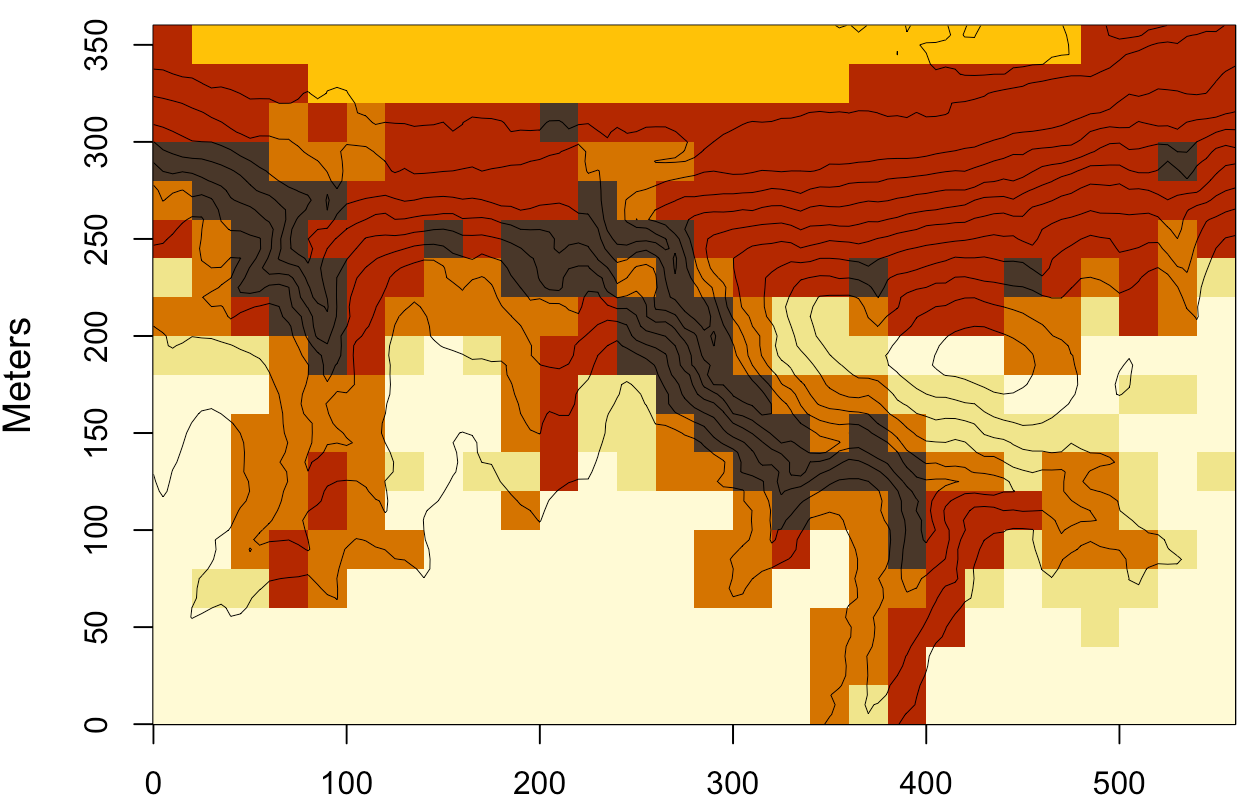

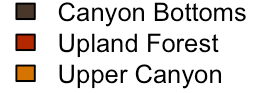

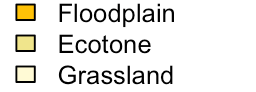

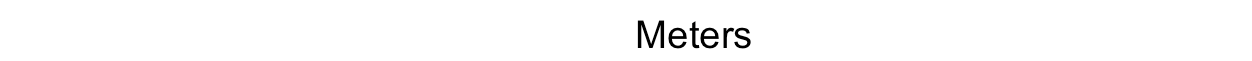


**b**

Figure S3. Derivation of categorical habitats based on topographic variables obtained from a digital elevation model (DEM) of the Niobrara plot. (a) Biplot of the first two principal components with points (20 x 20 m quadrats) colored by habitat, with arrows depicting the loadings of the topographic variables onto the PC axes; (b) a map of habitats in the Niobrara plot, overlaid with 5-m elevation contour lines. See Table S3 for the loadings of each variable onto PC1 and PC2, and Materials and methods: *Statistical analysis* for additional details on habitat classification method.

Grassland (10)

Floodplain (9)

Canyon Bottoms

Upland Forest

Upper Canyon


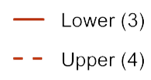

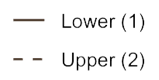

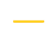

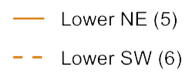

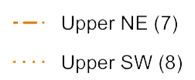

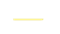

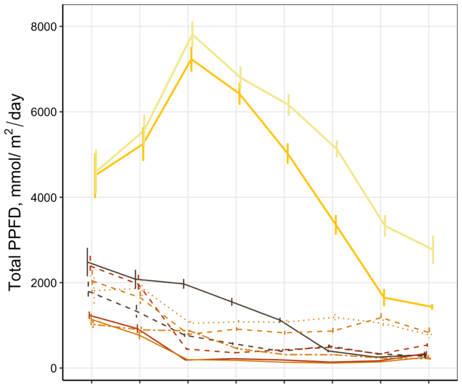

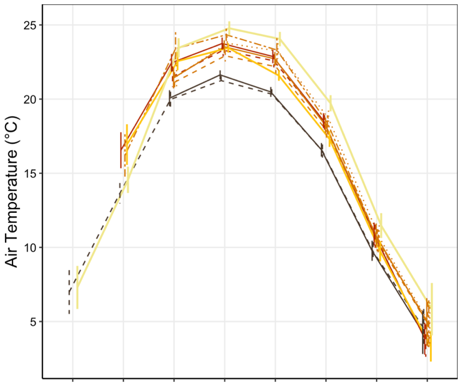

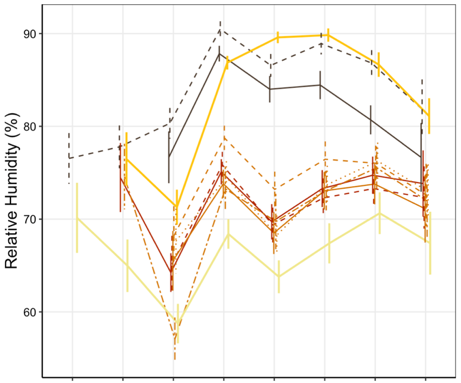

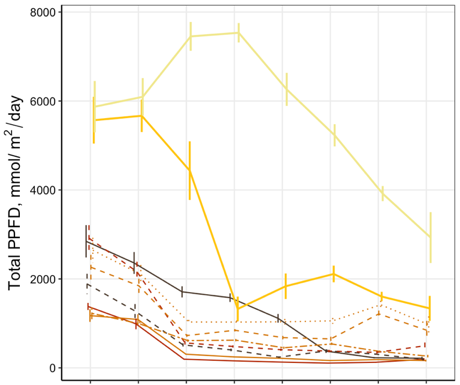


**g. 2022**


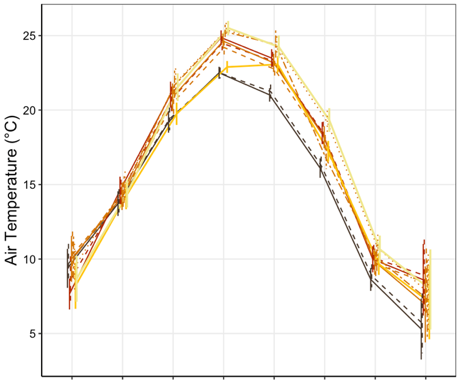

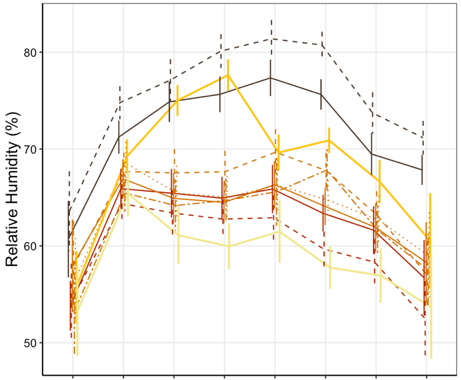


**a. 2021**

**b. 2021**

**c. 2021**

**h. 2022**

**i. 2022**


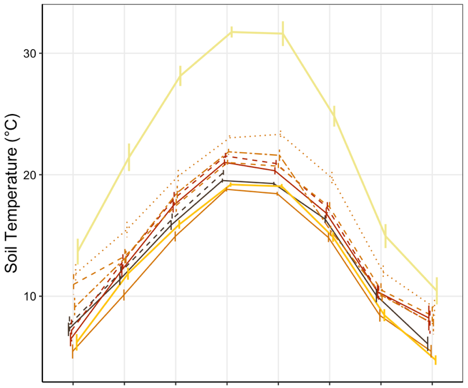

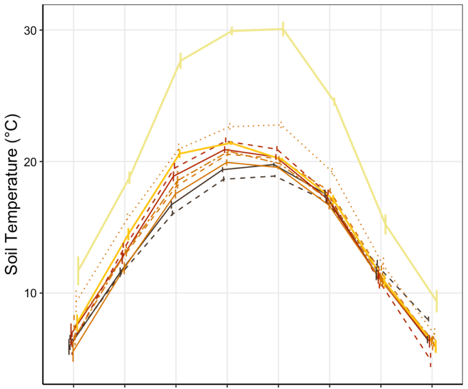

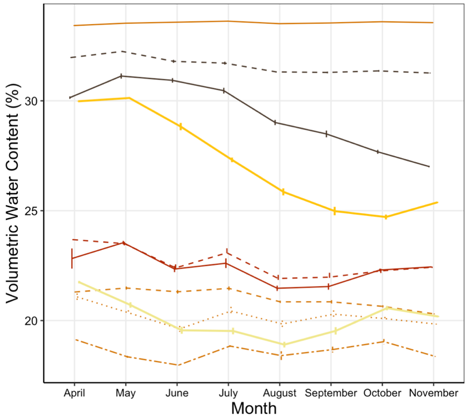

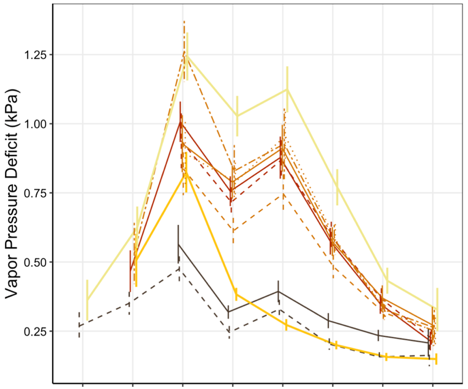

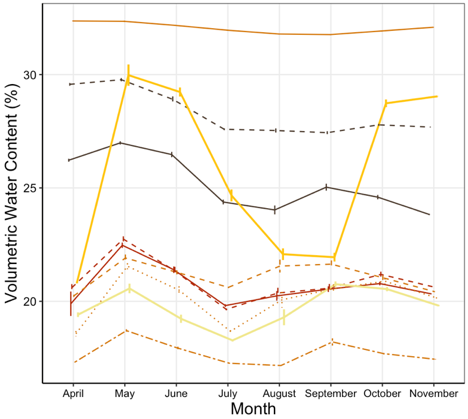

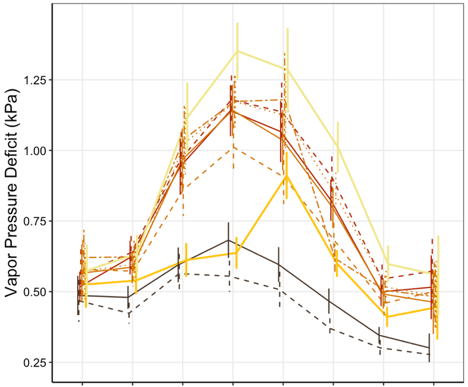


**d. 2021**

**e. 2021**

**f. 2021**

**j. 2022**

**k. 2022**

**l. 2022**


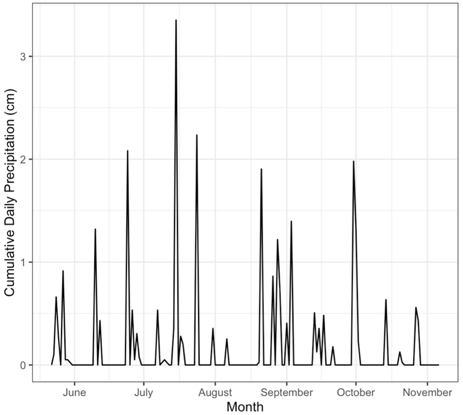

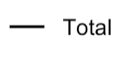

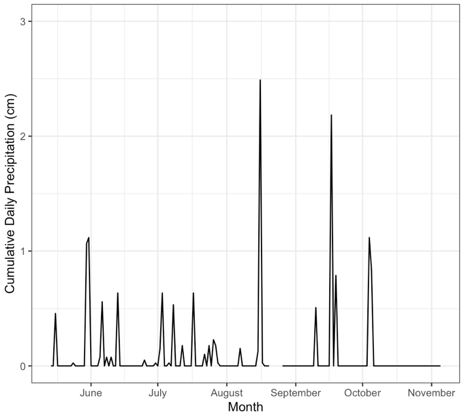


**m. 2021**

**n. 2022**

Figure S4. Habitat-related variation in microclimate and cumulative daily precipitation in the Niobrara plot. (a-l) Microclimate data collected at ten monitoring stations from April-November in 2021 (a-f) and 2022 (g-l). Monthly mean (± SE) values for (a,g) total daily photosynthetic photon flux density, (b,h) daily mean air temperature, (c,i) daily mean relative humidity, (d,j) daily mean vapor pressure deficit, (e,k) daily mean soil temperature, and (f,l) daily mean volumetric water content. In 2021, air temperature and relative humidity sensors were not deployed until 21 May for Stations 3, 7, and 9, and until 12 June for Stations 1, 4, 5, 6, and 8. In 2022, soil temperature data for Station 2 from August-November were excluded due to sensor error. (m-n) Daily precipitation (nearly all falling as rain) from 21 May-5 November in (m) 2021 and (n) 2022, collected at Station 10 (Fig. S2). Totals of 28.1 cm of precipitation fell in 2021 (daily average, 0.17 ± 0.46 cm (SD)) and 14.8 cm in 2022 (daily average, 0.09 ± 0.32 cm). Precipitation mostly occurred in large rainstorm events in both years, but for these, soil moisture only briefly increased before returning to drier conditions in all habitats but the canyon bottoms (f,1), suggesting benefits of these rainfall pulses for trees may have been short-lived. For (a-1), colors in the legend indicate habitats (Fig. S3b), and different line types of the same color indicate data from stations within that habitat, with station numbers indicated in the legend and corresponding to Fig. S2. In legend: NE = Northeast; SW = Southwest.


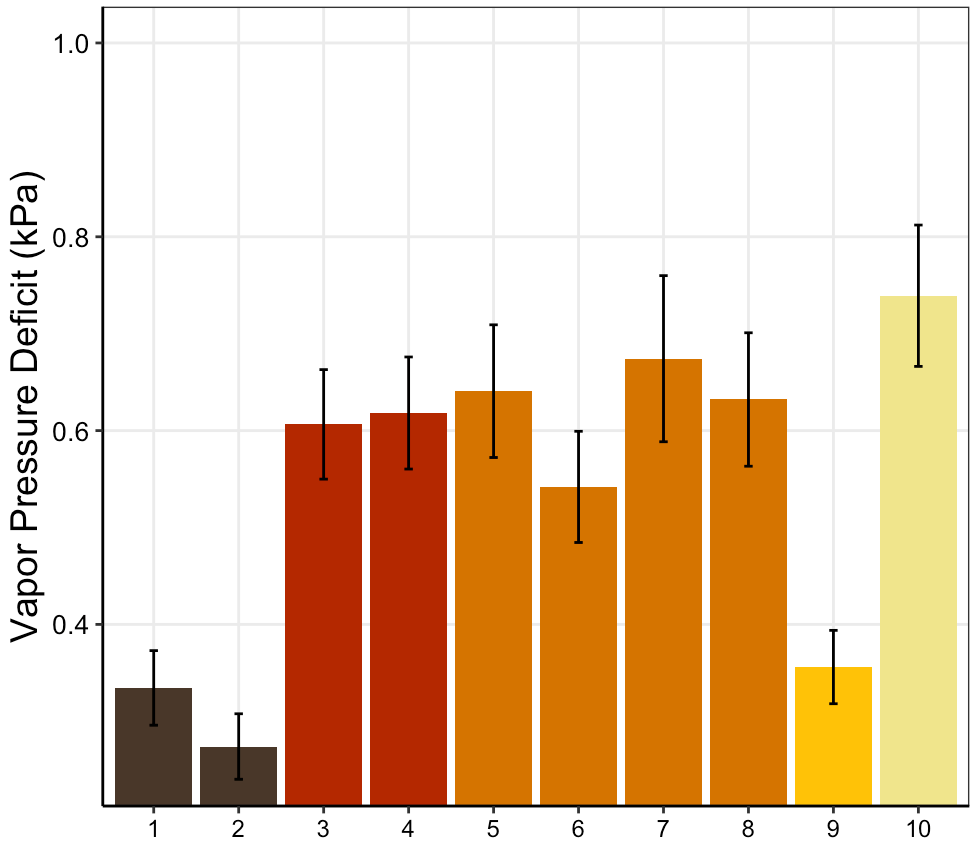

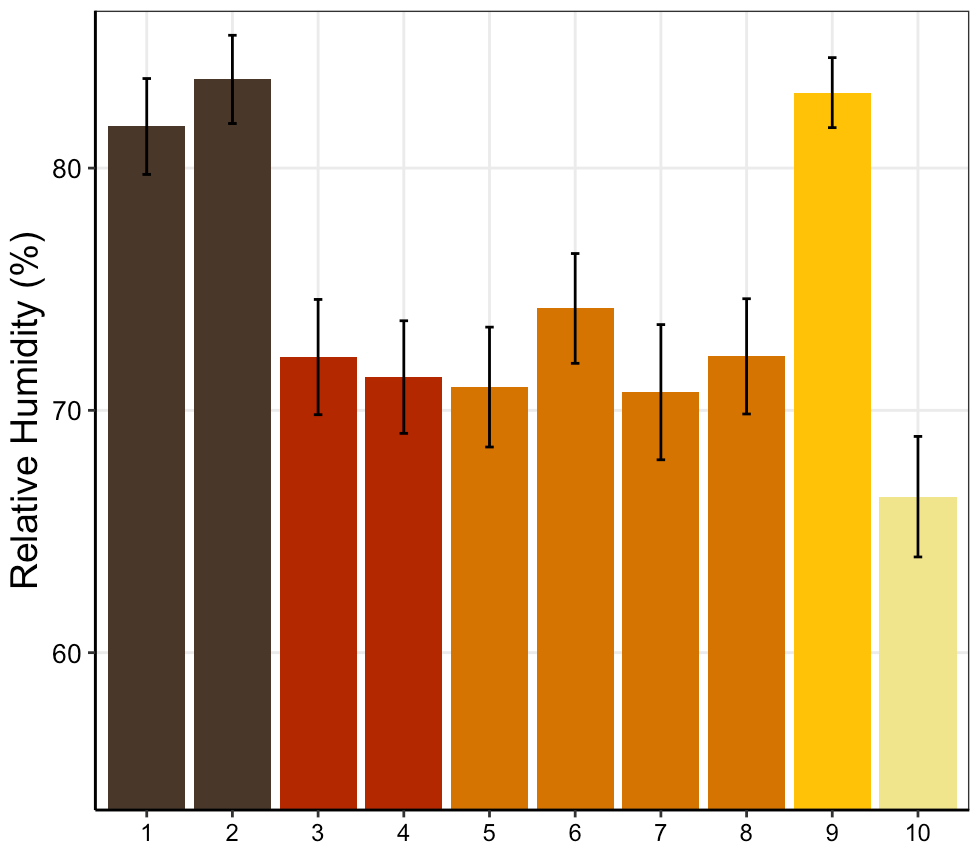

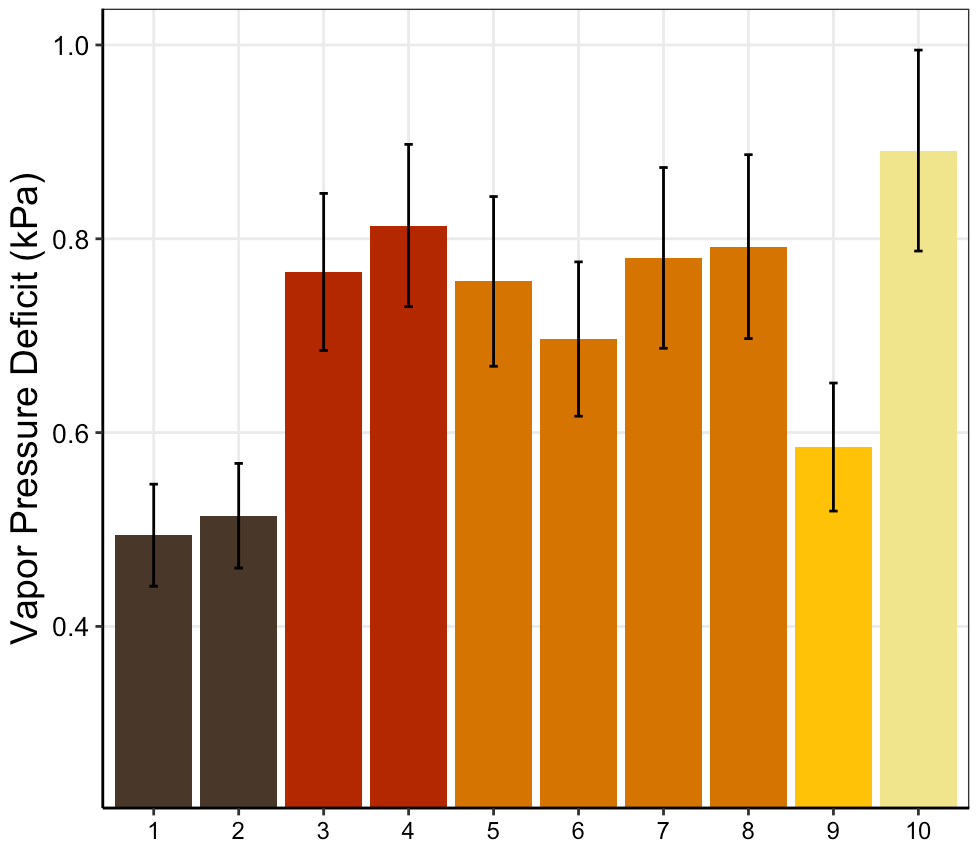

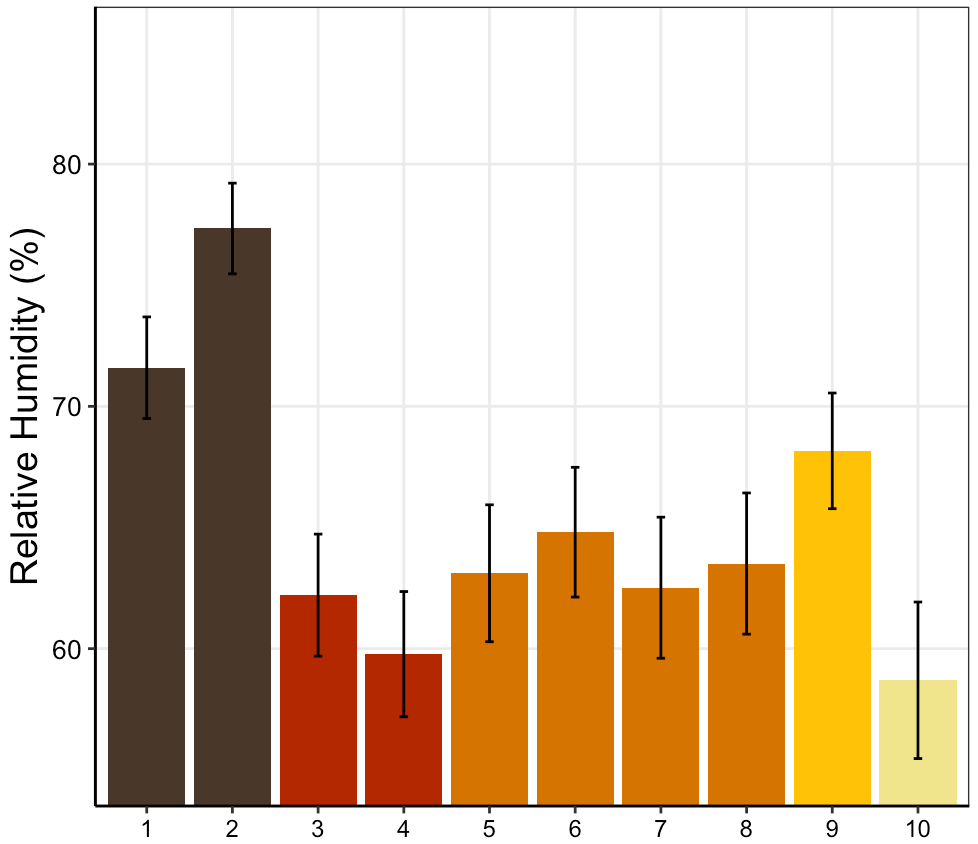

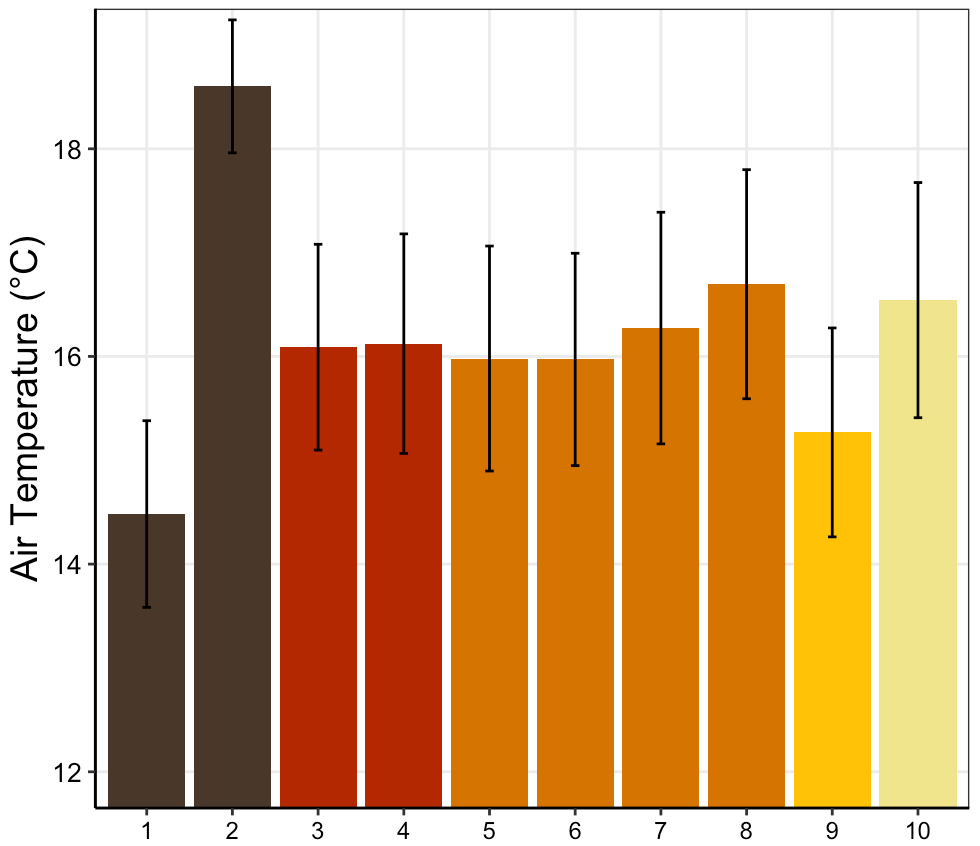

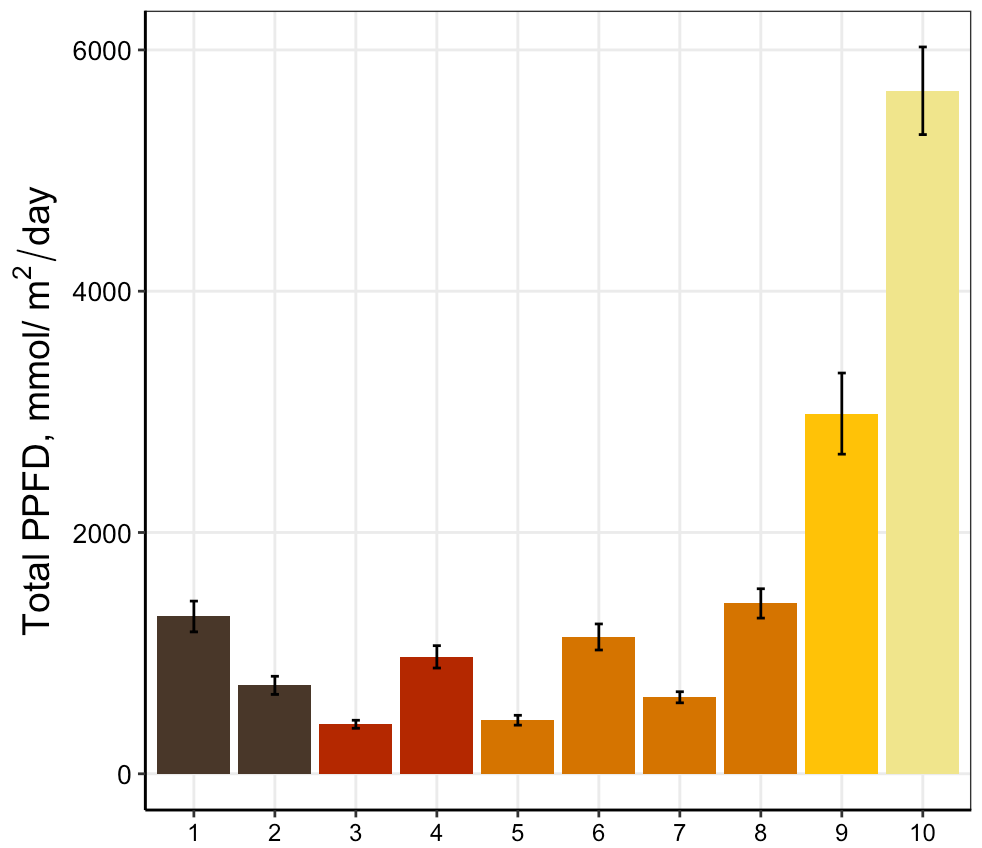

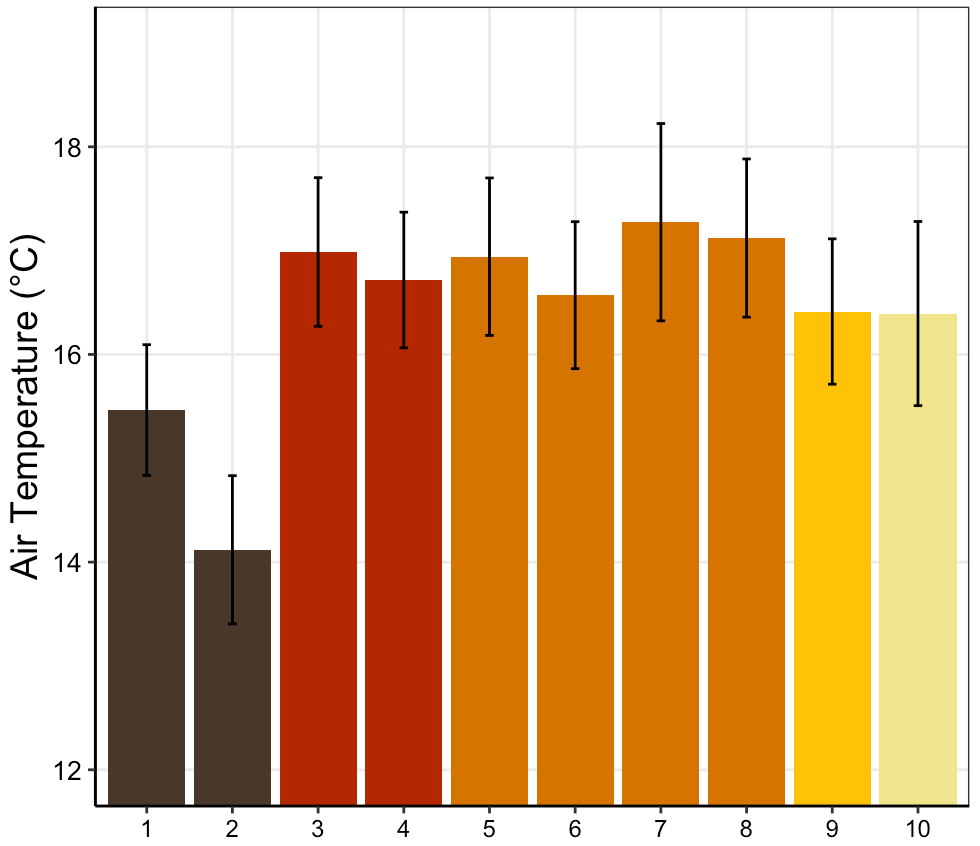

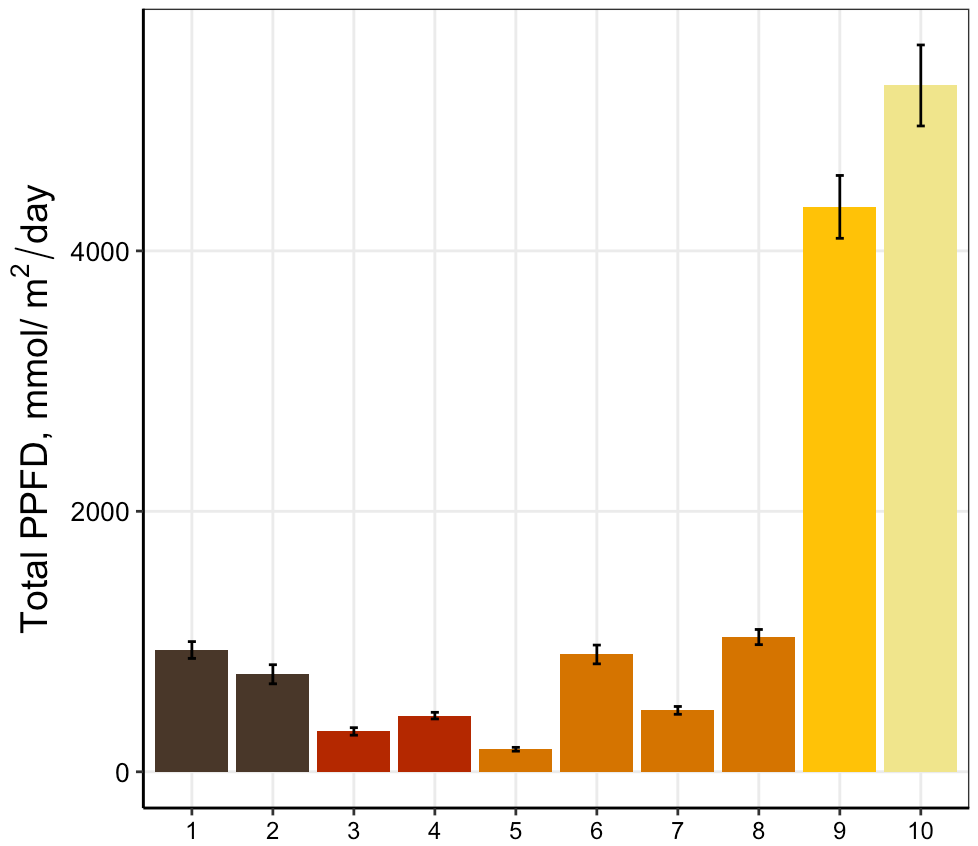


**d. 2021**

**j. 2022**

**g. 2022**

**a. 2021**

**b. 2021**

**c. 2021**

**h. 2022**

**i. 2022**


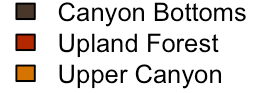

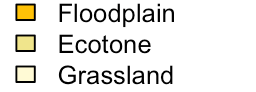


Canyon Bottoms

Upland Forest

Upper Canyon

Floodplain

Prairie

*****

*****

*****

*****

*****

*****

*****

*****

*****

*****

*****

*****

*****

*****

*****

*****

*****

*****

*****

*****

*****

*****

*****

*****


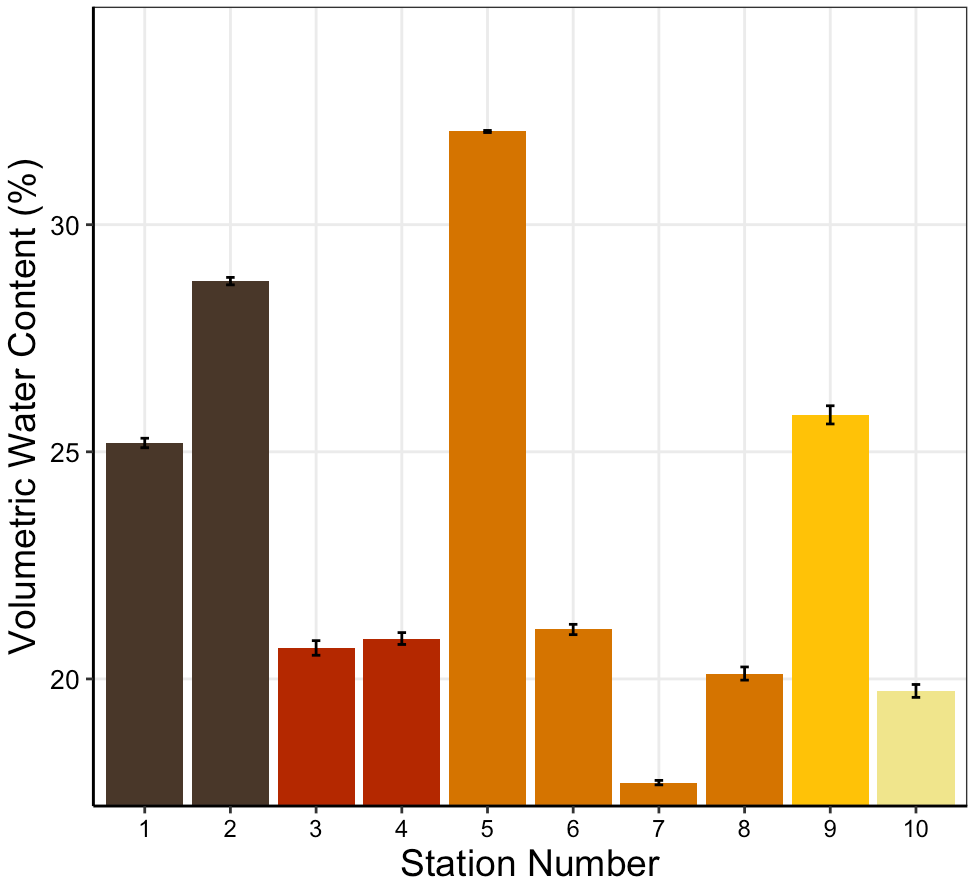

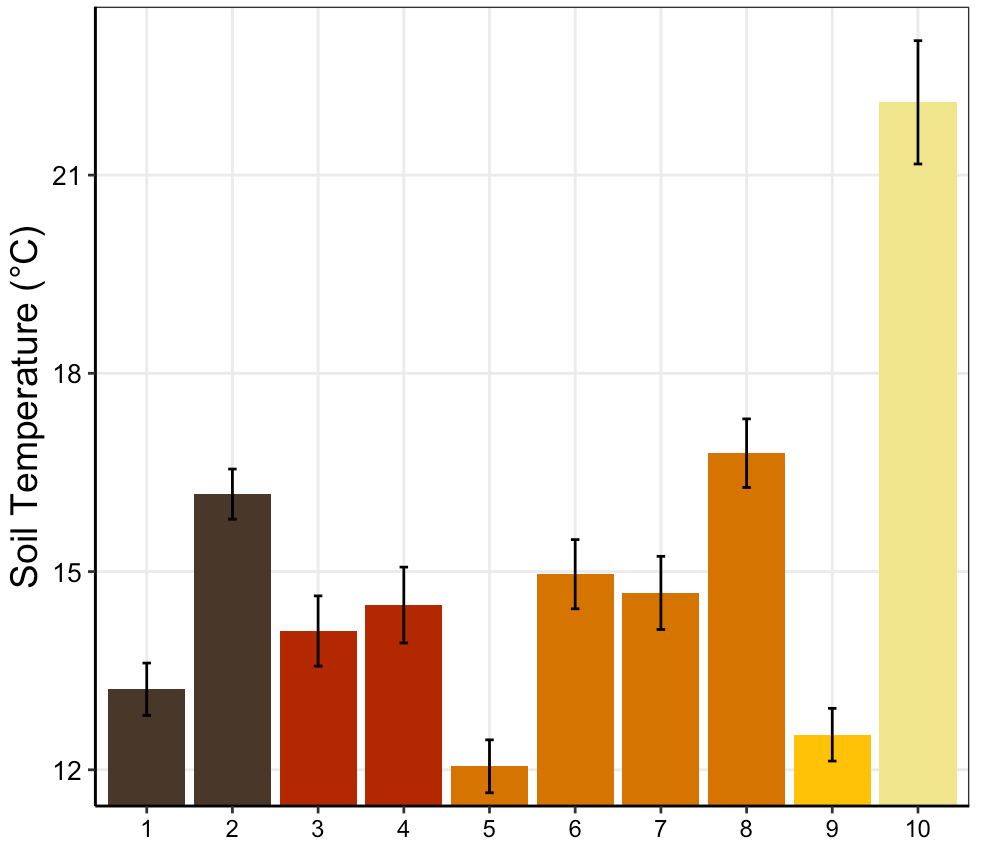

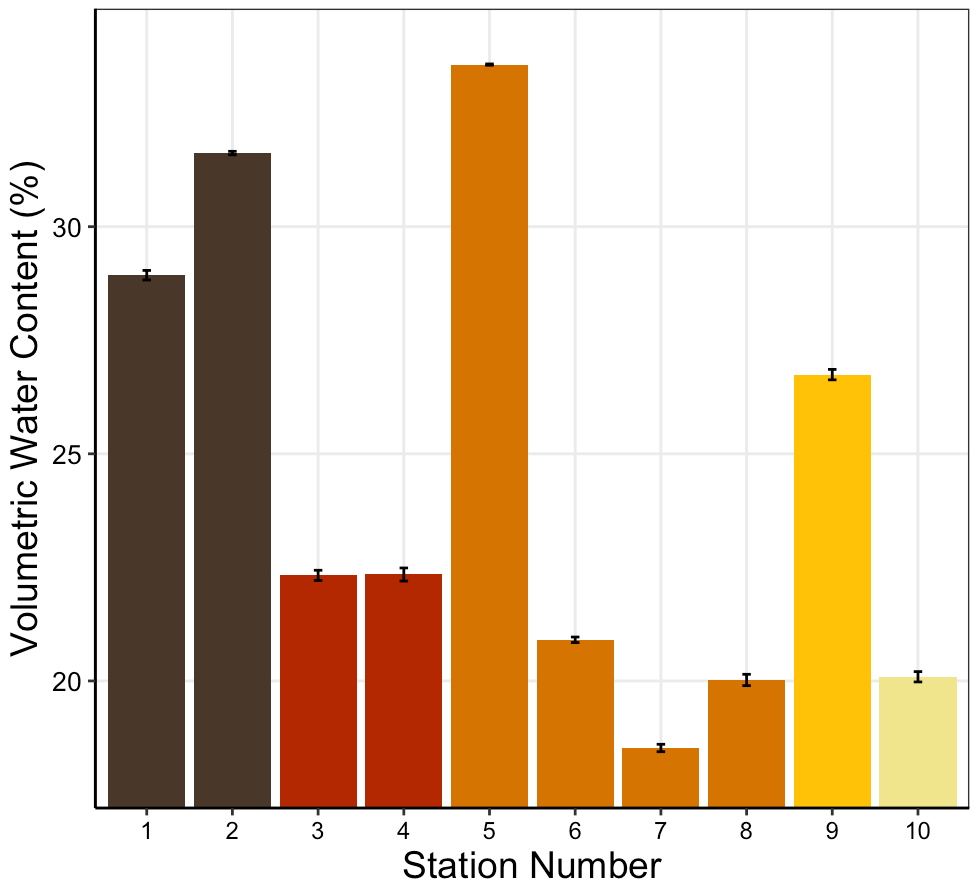

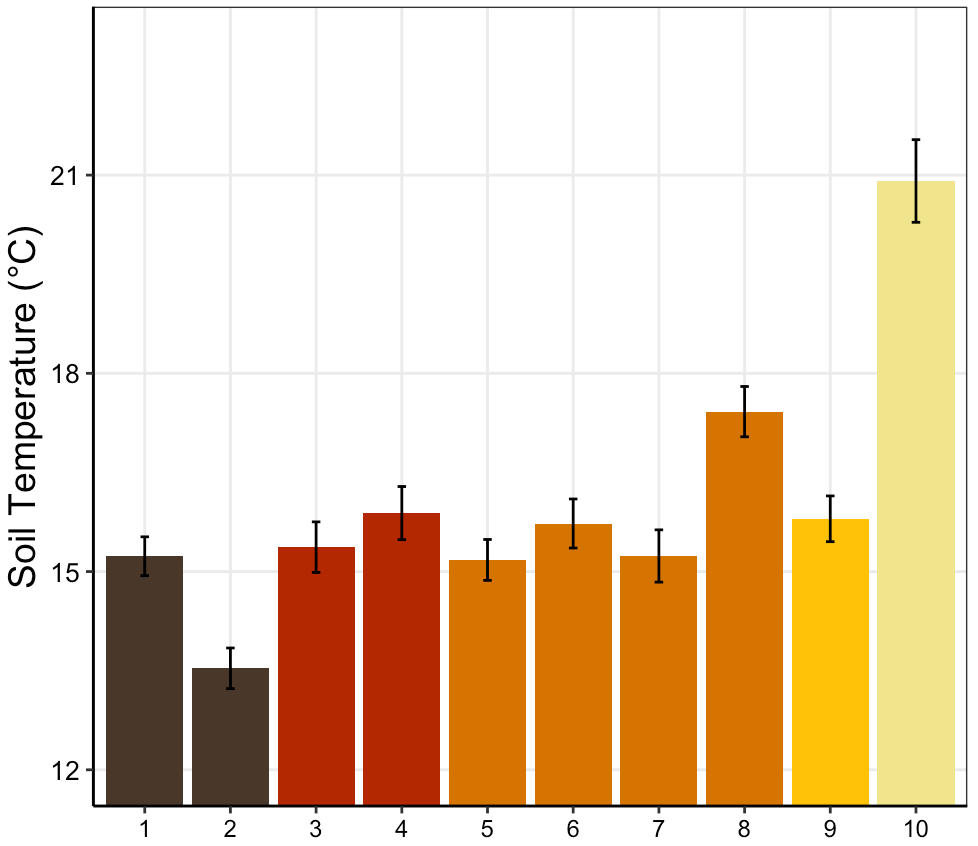


**e. 2021**

**f. 2021**

**k. 2022**

**l. 2022**

*****

*****

*****

*****

*****

*****

*****

*****

*****

*****

*****

*****

**Figure S5. Mean habitat-related microclimate variation in the Niobrara plot.** (a-l) Microclimate data collected at ten monitoring stations from April-November in 2021 (a-f) and 2022 (g-l). Mean growing season (± SE) values for (a,g) total daily photosynthetic photon flux density, (b,h) daily mean air temperature, (c,i) daily mean relative humidity, (d,j) daily mean vapor pressure deficit, (e,k) daily mean soil temperature, and (f,l) daily mean volumetric water content. In 2021, air temperature and relative humidity sensors were not deployed until 21 May for Stations 3, 7, and 9, and until 12 June for Stations 1, 4, 5, 6, and 8. In 2022, soil temperature data for Station 2 from August-November were excluded due to sensor error. Bars indicate data from the ten numbered stations within habitats, color-coded by habitat type as indicated in the legend. Asterisks near station numbers indicate monitoring stations located near a stream (Stations 1 and 2) or a spring (Station 5).

Grassland


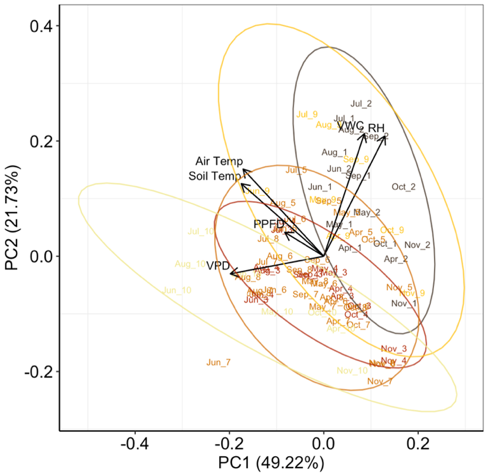

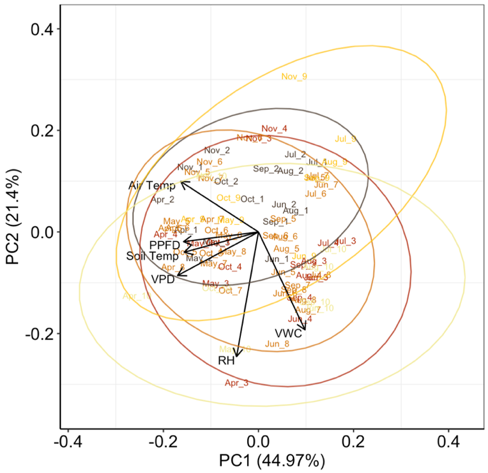

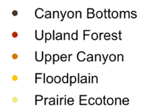

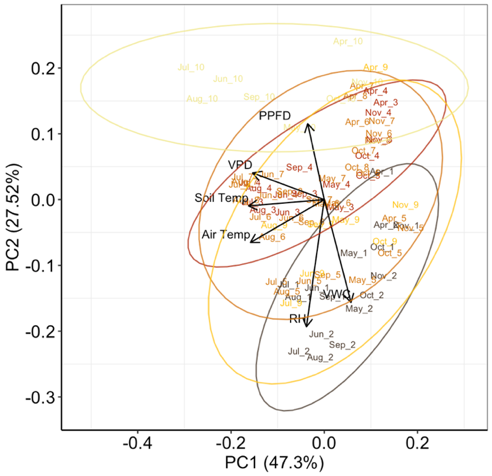

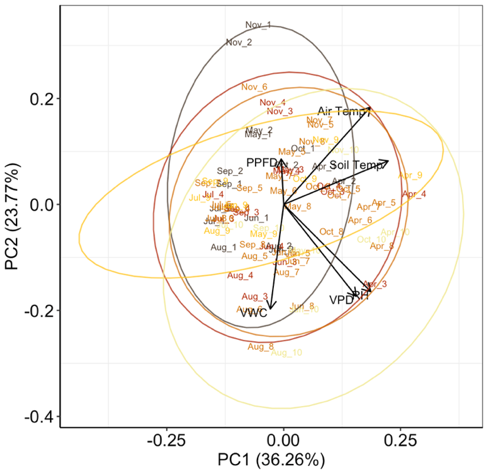


**a. 2021**

**b. 2022**

**c. 2021**

**d. 2022**

Figure S6. Multivariate habitat-related microclimate variation in the Niobrara plot, based on data collected at ten monitoring stations from April-November in 2021 and 2022. Principal Component 1 versus 2 (percent variance explained) from separate Principal Components Analyses on the 2021 (a,c) and 2022 (b,d) monthly (a,b) means and (c,d) coefficients of variation (CVs) of the microclimatic variables shown in Fig. S4. Each point represents a Month-Station (numbers are station numbers), colors indicate habitats, and ellipses are the 95% confidence intervals based on the standard error for each habitat. For 2021, means and CVs were imputed for the missing air temperature, relative humidity, and vapor pressure deficit values in April and May, as sensors measuring these variables were not deployed until 21 May for Stations 3, 7, and 9, and until 12 June for Stations 1, 4, 5, 6, and 8. For 2022, means and CVs were imputed for missing soil temperature data for Station 2 from August-November due to sensor error. PPFD = photosynthetic photon flux density (mmol/m^2^/day); air temperature = air temperature (°C) (c) daily mean); RH = relative humidity (%); VPD = vapor pressure deficit (kPa); soil temp = soil temperature (°C); VWC = volumetric water content (%). Both the effect of habitat type and the interaction between habitat type and month were significant in the perMANOVA on the microclimate means in 2021 (habitat: *F*_4,40_ = 52.7, *R^2^* = 0.39, *p* = 0.001; habitat × month: *F*_35,40_ = 8.4, *R^2^* = 0.54, *p* = 0.001) and 2022 (habitat: *F*_4,40_ = 39.5, *R^2^* = 0.32, *p* = 0.001; habitat × month: *F*_35,40_ = 8.2, *R^2^* = 0.59, *p* = 0.001), and in the perMANOVA on the microclimate CVs in 2021 (habitat: *F*_4,40_ = 11.2, *R^2^* = 0.11, *p* = 0.001; habitat × month: *F*_35,40_ = 9.4, *R^2^* = 0.80, *p* = 0.001) and 2022 (habitat: *F*_4,40_ = 13.1, *R^2^* = 0.10, *p* = 0.001; habitat × month: *F*_35,40_ = 12.9, *R^2^* = 0.83, *p* = 0.001).


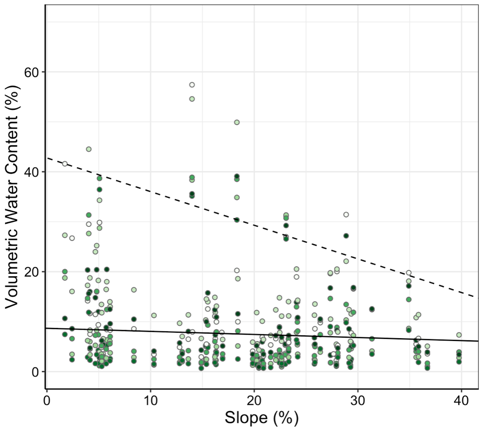


**a**

*pR^2^* = 0.18, slope = -0.06

*pR^2^* = 0.07, slope = -0.67


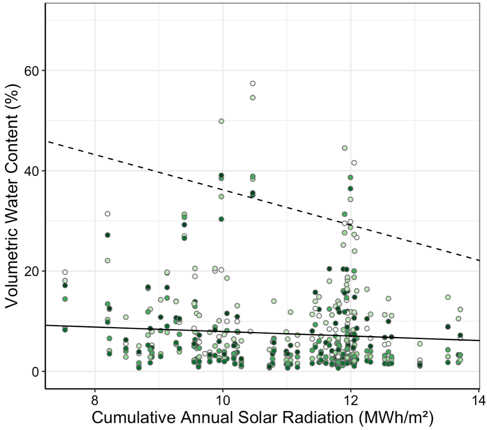


**b**

*pR^2^* = 0.18, slope = -0.45

*pR^2^* = 0.03, slope = -3.51


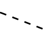


Median

95^th^


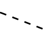


Median

95^th^

Figure S7. Variation in soil moisture across the 2021 growing season with respect to topographic variables. Scatterplots of VWC versus (a) slope and (b) cumulative annual solar radiation, where lines are predicted values for the median (solid line) and 95^th^ quantile (dashed line) relationships based on quantile regression models. *pR^2^* values in each panel are pseudo-*R^2^*. VWC declined with increasing slope, more so for moderate VWC values (median: *F*_6,420_ = 58.2, *p* < 0.001) than for higher values (95^th^ quantile: *F*_6,420_ = 1.1, *p* = 0.37). VWC declined with increasing solar radiation for moderate VWC values (median: *F*_6,420_ = 50.0, *p* < 0.001), but not for higher VWC values (95^th^ quantile: *F*_6,420_ = 1.0, *p* = 0.42).
